# Recommendations for the management of GnRH‐ant for ovarian stimulation in the assistant reproductive process: Benefits and harms for different ovarian response population

**DOI:** 10.1002/gin2.70022

**Published:** 2025-05-21

**Authors:** Rong Li, Rui Yang, Junhao Yan, Yang Song, Yunxia Cao, Zijiang Chen, Chenchen Xu, Zhan Zhao, Yichun Guan, Fei Gong, Guimin Hao, Hefeng Huang, Li Jin, Fenghua Liu, Jiayin Liu, Xiaoyan Liang, Xiaolin La, Yun Sun, Xiaohong Wang, Yanwen Xu, Cuilian Zhang, Jie Qiao

**Affiliations:** ^1^ Department of Obstetrics and Gynecology, Center for Reproductive Medicine Peking University Third Hospital Beijing China; ^2^ State Key Laboratory of Reproductive Medicine and Offspring Health, Center for Reproductive Medicine, Institute of Women, Children and Reproductive Health Shandong University Jinan China; ^3^ Institute of Digestive Disease and Department of Medicine and Therapeutics, State Key Laboratory of Digestive Disease, Li Ka Shing Institute of Health Sciences, CUHK Shenzhen Research Institute The Chinese University of Hong Kong Hong Kong China; ^4^ Department of Obstetrics and Gynecology The First Affiliated Hospital of Anhui Medical University Anhui China; ^5^ Tianjin Suyuan Evidence Based Technology Co., Ltd. China; ^6^ Center for Reproductive Medicine The Third Affiliated Hospital of Zhengzhou University Zhengzhou China; ^7^ Institute of Reproductive and Stem Cell Engineering, School of Basic Medical Science Central South University Changsha China; ^8^ Department of Reproductive Medicine, Hebei Key Laboratory of Infertility and Genetics, Hebei Clinical Research Center for Birth Defects, Hebei Medical Key Discipline of Reproductive Medicine, Hebei Collaborative Innovation Center of Integrated Traditional and Western Medicine on Reproductive Disease The Second Hospital of Hebei Medical University Shijiazhuang China; ^9^ Obstetrics and Gynecology Hospital, State Key Laboratory of Genetic Engineering and School of Life Sciences Fudan University Shanghai China; ^10^ Department of Reproductive Health and Infertility Guangdong Women and Children Hospital, Guangzhou Guangdong China; ^11^ Department of Reproductive Medicine First Affiliated Hospital of Nanjing Medical University/Jiangsu Province Hospital Nanjing China; ^12^ Reproductive Centre, The Sixth Affiliated Hospital Guangzhou Sun Yat‐Sen University Guangdong China; ^13^ Center for Reproductive Medicine First Affiliated Hospital of Xinjiang Medical University Urumqi China; ^14^ Department of Reproductive Medicine Ren Ji Hospital, Shanghai Jiao Tong University School of Medicine Shanghai China; ^15^ Reproductive Medical Center Tangdu Hospital, Air Force Medical University Shaanxi China; ^16^ Reproductive Medicine Center the First Affiliated Hospital of Sun Yat‐sen University, Guangzhou Guangdong China; ^17^ Department of Reproductive Medicine Center People's Hospital of Zhengzhou University Zhengzhou Henan China

**Keywords:** controlled ovarian stimulation, GnRH‐ant, guideline, in vitro fertilization, pretreatment, trigger, uteal support

## Abstract

**Introduction:** Gonadotropin‐releasing hormone antagonists (GnRH‐ant) are commonly used during controlled ovarian stimulation (COS) in the in vitro fertilization (IVF) process to prevent premature luteinization and to ensure follicles mature synchronously. The 2020 guideline from European Society of Human Reproduction and Embryology (ESHRE) recommends the use of GnRH‐ant for patients with varying levels of ovarian response. However, the question of how to manage the protocol pathway for these patients requires further investigation.

**Methods:** The current clinical practice guideline (CPG) adheres to the World Health Organization's (WHO) recommended development process, and covers eight clinical questions, all focusing on the application of GnRH antagonists in COS. We conducted Cochrane‐standard systematic reviews, utilized GRADE for evidence certainty assessment, and employed GRADE Evidence‐to‐Decision Framework to derive recommendations. We pre‐defined clinically important outcomes, and the common critical outcomes shared by all clinical questions as follows: live birth rate, implantation rate, clinical pregnancy rate, and moderate to severe ovarian hyperstimulation syndrome (OHSS). We strictly followed the RIGHT guideline and AGREE‐II criteria throughout the CPG development.

**Recommendations:** The guideline development group (GDG) agreed on 14 recommendations on the application of GnRH‐ant during COS. In summary, low certainty of evidence supported the benefits of using GnRH‐ant protocol in IVF patients with high ovarian response (HOR), following with very low certainty of evidence to use Oral Contraceptive Pill (OCP) or estrogen as pretreatment in normal ovarian response (NOR) patients, low certainty of evidence to use fixed GnRH‐ant protocol and very low certainty of evidence to use GnRH agonist (GnRH‐a) add on trigger. For patients using GnRH‐ant protocol, very low certainty of evidence in HOR patients and low certainty of evidence in NOR patients supported the “freeze‐all” strategy considering the potential risk associated with fresh embryo transfer. However, multiple fresh embryo transfer may still provide benefits in some cases.

## Key points

**Objective & Scope:** This clinical practice guideline (CPG) provides evidence‐based recommendations for managing GnRH antagonist (GnRH‐ant) protocols during controlled ovarian stimulation (COS) in IVF/ICSI, focusing on optimizing outcomes for patients with high (HOR), normal (NOR), and poor ovarian response (POR).

**Key Recommendations:** HOR People: Prioritize GnRH‐ant over agonist protocols to reduce moderate to severe OHSS risk. Strongly recommend a “freeze‐all” strategy to avoid fresh embryo transfer due to higher OHSS risk and lower live birth rates. NOR People: Do not prioritize the use of GnRH‐ant over agonists. Pretreatment (e.g., oral contraceptives/estrogen) and using GnRH agonist (GnRHa) trigger alone are not recommended. Tentatively suggest fixed GnRH‐ant protocols, dual trigger (GnRHat + hCG) and freeze‐all strategies to balance risks. Immediate luteal support after oocyte retrieval in fresh cycles with GnRH agonist trigger may benefit NOR people. POR People: Does not prioritize the use of the antagonist protocol. Cautious use of pretreatment (e.g., androgens/antagonists) may improve outcomes.

**Evidence & Methodology:** The systematic review team conducted a thorough and comprehensive search of the relevant research evidence. We used the GRADE (Grading of Recommendations, Assessment, Development, and Evaluation) methodology to systematically assess the certainty of evidence and grade our recommendations. Utilized Cochrane‐standard systematic reviews, GRADE framework and employed the GRADE Evidence to Decision Framework (EtD) to facilitate the formulation of the recommendation.

**Practical Considerations:** Emphasizes individualized decision‐making, considering patient preferences and resource availability. Highlights gaps in evidence for D3 versus D5 embryo transfer, and economic benefits and patient preferences. Comparison to ESHRE 2020: This guideline offers more granular recommendations tailored to ovarian response subgroups, particularly advocating GnRH‐ant for HOR patients to mitigate OHSS.

## 1 INTRODUCTION AND SCOPE

The scope of the current clinical practice guideline (CPG) focuses on the clinical management of GnRH antagonist protocols in assisted reproductive therapy. The CPG covers eight clinical questions, all focusing on the application of GnRH antagonists in controlled ovarian stimulation (COS) (Table 1).

**Table 1:** Recommendations summary.

Recommendations	Strength of evidence and recommendation
1.1 For people with normal or low ovarian response, compared to the agonist protocol, the GDG does not prioritize the use of the antagonist protocol.	Conditional recommendation⨁⨁**◯◯**
1.2 For people with high ovarian response, the GDG suggests prioritizing the use of the antagonist protocol.	Conditional recommendation⨁⨁**◯◯**
2.1 For people with normal ovarian response, the GDG does not prioritize Pretreatment before ovarian stimulation.	Conditional recommendation⨁**◯◯◯**
2.2 For people with low ovarian response, the GDG recommends cautious use of Pretreatment before ovarian stimulation.	Conditional recommendation⨁⨁**◯◯**
3.1 For people with normal ovarian response, the GDG tentatively suggests the fixed antagonist protocol over flexible protocol, with the provision that adjustment is considered following discussion and full consideration of the individuals' specific situation.	Conditional recommendation⨁⨁**◯◯**
3.2 For people with high ovarian response, there is insufficient evidence to either support or refute the use of fixed antagonist protocol compared to the flexible protocol. The GDG suggests collaborative decisions with the stakeholders, exercising caution, and with full consideration of each individual's specific situation.	Conditional recommendation for either the intervention or the comparison
4. For people with varying ovarian responses, the GDG notes there is insufficient evidence to demonstrate the advantage of early initiation of GnRH‐ant, and therefore, refrains from making a recommendation. Early initiation of the GnRH‐ant protocol should only be considered if conventional protocol has failed, and this decision should be made with reference to follicle size consistency. Clinicians are encouraged to adjust practice based on the specific situations of the individual.	Conditional recommendation for either the intervention or the comparison
5.1 For people with normal ovarian response, compared to using HCG trigger alone, the GDG tentatively suggests adding GnRHa trigger. Clinicians may adjust clinical practice based on individual preferences and previous assisted reproduction outcomes.	Conditional recommendatio⨁**◯◯◯**
5.2 For people with normal ovarian response, the GDG recommends against using GnRHa trigger alone.	Strong recommendation⨁**◯◯◯**
6.1 Regarding the need for enhanced luteal support after using the GnRH agonist trigger in antagonist protocol, there is a lack of evidence, and the GDG makes conditional recommendation for either the intervention or the comparison on enhanced luteal support.	Conditional recommendation for either the intervention or the comparison
6.2 For people with normal ovarian response, compared to starting luteal support 2‐5 days after oocyte retrieval, the GDG suggests immediate luteal support after oocyte retrieval in fresh cycles with GnRH agonist trigger to improve pregnancy success rates and reduce miscarriage rates.	Conditional recommendation⨁**◯◯◯**
7.1 For people with high ovarian response undergoing COS with the GnRH antagonist protocol, the GDG recommends freeze‐all regimen and cancellation of fresh embryo transfer (strong recommendation, low‐certainty evidence). In the subgroup of single blastocyst transfer, while benefit of fresh single blastocyst transfer is unclear, the harm is significant (reduced live birth and clinical pregnancy rates), with a tendency towards frozen‐thawed single blastocyst transfer. In the subgroup of multiple cleavage stage embryo transfers, the clinical benefit of fresh embryo transfer is significant (reduced risk of pre‐eclampsia and pregnancy‐induced hypertension). However, the disadvantages are also evident (increased moderate to severe OHSS, reduced live birth and clinical pregnancy rates). Clinicians may adjust clinical practice based on individual preferences and previous assisted reproduction outcomes.	Strong recommendation⨁**◯◯◯**
7.2 For people with normal ovarian response undergoing COS with the GnRH antagonist protocol, the GDG tentatively suggests freeze‐all regimen. In the subgroup of single blastocyst transfer, the benefit of fresh embryo transfer is moderate (reduced risk of pre‐eclampsia), but as the disadvantages are significant (greatly reduced implantation and clinical pregnancy rates), there may be a preference for frozen embryo transfer. In the subgroup of double/multiple cleavage stage transfers, the benefit of fresh embryo transfer is moderate (increased live birth, clinical pregnancy, implantation rates, and reduced risk of pre‐eclampsia), accompanied by moderate disadvantages (increased moderate to severe OHSS). Overall, there may be a preference for frozen embryo transfer. Clinicians may adjust clinical practice based on patient preferences and previous assisted reproduction outcomes.	Conditional recommendation⨁⨁**◯◯**
8. For people undergoing COS with the GnRH antagonist protocol, there is limited evidence regarding the choice between D3 and D5 embryo transfer. After thorough discussion, the GDG unanimously agreed not to make preferential recommendation between D3 and D5 single embryo transfer.	Conditional recommendation for either the intervention or the comparison

*Note*: Very low certainty evidence = ⨁◯◯◯; Low certainty evidence = ⨁⨁◯◯.

### 1.1 Health problem description

Ovarian stimulation, or controlled ovarian stimulation (COS), is a crucial step in the in vitro fertilization (IVF) process. This procedure involves administering hormone injections to stimulate the ovaries to produce multiple eggs simultaneously, thereby increasing the chances of retrieving more eggs for fertilization.^1^ The goal is to optimize the number of mature eggs retrieved in a single cycle, mimicking the natural ovulation process, but with enhanced egg production. This approach helps maximize the chances of creating viable embryos for implantation.

During COS in Assisted Reproductive Technology (ART), Gonadotropin‐releasing hormone (GnRH) antagonists are commonly used to prevent premature luteinization by inhibiting the release of endogenous FSH and LH from the pituitary gland.^2^ This suppression ensures the follicles mature synchronously, avoiding early luteinization which could lead to follicle maturation arrest or asynchrony in oocyte development. Despite their advantages, the use of GnRH antagonists also presents certain challenges,^3^ particularly regarding the optimal dosing regimens and their effects on clinical outcomes, which are still subjects of ongoing research.^4^

In 2020, the European Society of Human Reproduction and Embryology provided similar recommendations without specific protocols.^5^ To address this gap, the Chinese Medical Doctor Association's Reproductive Medicine Sub‐Association developed a clinical practice guideline with detailed recommendations tailored to different ovarian response groups, aimed at guiding clinical practice.

## 2 GUIDELINE'S OBJECTIVES AND PURPOSE

### 2.1 Objective

We aim to develop evidence‐based CPGs for the use of GnRH‐ant in COS during IVF, focusing on optimal protocols for varying ovarian responses. The guidelines aim to improve patient outcomes by providing clear recommendations for clinicians and healthcare providers.

### 2.2 Target population

The target population is female patients undergoing in vitro fertilization (IVF) or intracytoplasmic sperm injection (ICSI).

### 2.3 Target users

The target guideline users are physicians, including reproductive medicine specialists; gynecologists; family physicians; nurses, including registered nurses and nurse practitioners; medical trainees, including medical students, residents, and fellows; and all other health care providers.

## 3 METHODS

### 3.1 Prospective registration and guideline report

The CPG is registered at the Guideline International Network (GIN), and registration can be accessed via the following link: https://guidelines.ebmportal.com. We followed the AGREE checklist to conduct, and the RIGHT checklist to report this guideline.^6^

### 3.2 Description of panel composition

The guideline development group (GDG) consist of specialists from reproductive medicine and obstetrics/gynecology across mainland China, recommended by the clinical chair of the guideline. Please refer to Appendix 1 for further information on their clinical specialty and geographical location.

### 3.3 Questions and outcomes

Please refer to Appendix 2 for a full list of clinical questions and the PICO breakdown.

For each clinical question, the GDG prioritized outcomes based on clinical importance through online survey (http://www.wenjuanxing.cn), using a 9‐point scale. The critical outcomes shared by 8 PICOs are live birth rate, implantation rate, clinical pregnancy rate, moderate to severe ovarian hyperstimulation syndrome (OHSS). Furthermore, the GDG pre‐defined the magnitude of effect through consensus upon the recommendation formulation. For live birth rate, implantation rate, and clinical pregnancy rate, the panel considered < 25 cases per 1000 people as a minimal effect, 25 to 50 cases per 1000 people as moderate effect, and > 50 cases per 1000 as large effect. Whereas, for moderate to severe ovarian hyperstimulation syndrome (OHSS), the panel considered < 10 cases per 1000 people as a minimal effect, 10 to 20 cases per 1000 people as moderate effect, and > 50 cases per 1000 as large effect.

### 3.4 Literature searches and evidence eligibility

The systematic review team conducted a thorough and comprehensive search of the relevant research evidence. It evaluated benefits/risks of interventions or other contextual factors (e.g., costs, cost‐effectiveness, values and preferences, equity) for each clinical question addressed in the CPG, with a particular emphasis on evidence from China. The databases searched included China National Knowledge Infrastructure (CNKI), Wanfang Data, Chinese Biomedical Literature Database (CBM), VIP Database, PubMed, Embase, Web of Science, and the Cochrane Library. The initial search covered literature from the inception of each database up to July 1, 2021. This was followed by an updated search, extending the coverage date to December 31, 2022. The full search strategy and report is available in Appendix 3. In addition to database searches, the team examined studies included in relevant systematic reviews. It also consulted with the GDG members to identify additional key studies (both published and unpublished) potentially eligible for inclusion.

For each clinical question, inclusion and exclusion criteria were established before screening the literature, and dataextraction forms were designed using Excel.^7^ Randomized controlled trials (RCTs) were prioritized for inclusion. When RCT evidence was lacking, indirect, or of very low certainty, observational or noncontrolled studies were included as supplementary evidence. Two reviewers (ZZ, CCX) independently screened the literature (title, abstract, and full text) and extracted data. In case of disagreement, a third reviewer was consulted. The procedure of study selection was presented in the PRISMA flow diagram (Appendix 4). The certainty of the included RCTs was assessed using the Cochrane risk of bias (RoB) tool.^8^ The RoB tool was developed by the Cochrane Collaboration to assess the risk of bias in randomized trials. It is a domain‐based evaluation tool that focuses on specific aspects of bias such as: selection, performance, detection, attrition, and reporting.

### 3.5 Evidence synthesis methods

For comparative controlled studies, meta‐analyses were conducted using a random effects model in RevMan 5.3 software.^9^ We calculated relative risks (RR) and their 95% confidence intervals (CI) for binary outcomes, and mean differences (MD) with 95% CI for continuous outcomes. Before performing pooled analyses, we thoroughly assessed both clinical and methodological heterogeneity between studies to ensure inconsistency. Studies that could not be pooled due to significant differences were analyzed descriptively. Given the notable variations in reproductive and health outcomes among different ovarian response groups, we established predefined subgroups based on ovarian response levels, including normal ovarian response (NOR), poor ovarian response (POR), high ovarian response (HOR).

To evaluate statistical heterogeneity, we employed the Chi‐squared test and the I² statistic. Heterogeneity was considered significant if the Chi‐squared test yielded a *p*‐value of less than 0.1 and the I² statistic exceeded 50%, indicating substantial variability among the study results.^9^ The result of meta‐analysis is available in Appendix 5.

### 3.6 Certainty of evidence assessment

We used the GRADE (Grading of Recommendations, Assessment, Development, and Evaluation) methodology to systematically assess the certainty of evidence and grade our recommendations.^10^ The GRADE approach is a comprehensive framework that involves two approaches. The first approach focuses on rating the certainty of evidence. This process entails a detailed evaluation of several factors that can affect the reliability and validity of the evidence, including risk of bias, inconsistency, indirectness, imprecision, and publication bias. These factors help categorize the certainty of evidence into four levels: high, moderate, low, and very low. High‐certainty evidence indicates a high level of confidence that the true effect is close to the estimate of the effect, while very low‐certainty evidence suggests that any estimate of effect is very uncertain. The summary of the evidence is presented in summary of findings (SoF) tables in Appendix 6, which report the relative and absolute effect estimates per outcome and the certainty of the evidence.

### 3.7 Recommendation grading

We employed the GRADE Evidence to Decision Framework (EtD) in Appendix 7, to facilitate the formulation of the recommendation process.^11^ The EtD framework integrates several critical factors to develop robust and actionable recommendations, make the decision‐making process explicit and transparent, providing clarity on the judgments made and the supporting evidence. According to GRADE,^12^ Recommendations are categorized as either strong or weak (conditional). A strong recommendation is made when the desirable effects of an intervention clearly outweigh the undesirable effects, or vice versa, and there is high confidence in the evidence. A weak or conditional recommendation is given when the balance between benefits and harms is less certain, or there is lower confidence in the evidence.

### 3.8 Methods for formulating recommendations (Decision‐making process)

Nineteen GDG members reviewed and discussed the evidence summaries, evidence grading, and evidence interpretations through three video conferences on September 1, September 25, and November 29, 2023. Methodologist and the systematic review team collaborated with the clinical experts to discuss the relevant evidence in detail, considering factors such as the balance of benefits and harms of the interventions and control measures, the certainty of evidence, patient values and preferences, resources use, cost‐effectiveness, the impact of interventions on equity and fairness in social and healthcare resources, and the acceptability and feasibility of the interventions. Through these discussions, the participating GDG members reached consensus on the direction and strength of the recommendations for each clinical question.

Consensus on the cut‐off points (e.g. cut‐offs of the magnitude of effect on critical outcomes) was reached through panel discussions and open dialogue, primarily informed by clinical experience and insight, as no existing literature directly addressed these cut‐offs. The consensus for formulating recommendations was set at >80% agreement and achieved through open discussion and deliberation.

### 3.9 Conflicts of interest management

The management of conflicts of interest (COIs) for members of the guideline working group and the GDG, including clinical experts and methodologists, follows the principles outlined by the World Health Organization (WHO) and the Guidelines International Network (GIN).^13^ Members of the guideline working group and the GDG disclosed their potential COIs to the guideline commissioner, including any financial, intellectual property, or other interests related to the guideline recommendations. Financial interests included employment, consultancy roles, research funding, or investments in entities connected to the project. Intellectual property interests, such as patents, trademarks, or affiliations with competing products or organizations, had to be declared. Additionally, other potential conflicts, such as work with competitors, third‐party support (e.g., travel expenses), and payments for public speaking, with the potential to influence judgment, needed to be disclosed. The exact threshold for what constitutes a significant COI varies by organization, and in the current CPG we adopted a monetary value of over 5000 USD or direct involvement with relevant product as a cut‐off for substantial COI.

The COI was collected and assessed at the beginning of the guideline process, once the panel members were selected, and again at the end of the process after the external peer review. Panel members were asked to update the project group of any changes to their COI status during the project. None of the working group members, nor the GDG members had any substantial COIs and were therefore approved to participate fully in the guideline development process.

### 3.10 Funding source and funder role

The funding for the development of this guideline was provided by the China Primary Health Care Foundation, with no additional funds or commercial sponsorship. The funder had no influence on the content of this guideline.

## 4 RECOMMENDATIONS

### 4.1 PICO 1. Should the GnRH‐ant protocol be recommended over the GnRH agonist (GnRH‐a) protocols for people undergoing COS?

#### 4.1.1 Recommendation 1

- For people with NOR or POR, the GDG does not suggest prioritizing the use of the antagonist protocol compared to the agonist protocol (conditional recommendation, low‐certainty evidence).
- For people with HOR, the GDG suggests prioritizing the use of the antagonist protocol (conditional recommendation, low‐certainty evidence).

#### 4.1.2 Implementation suggestions

GnRH antagonists:

Ganirelix acetate: 0.25 mg, administered subcutaneously, starting from the 6th day of stimulation until the day of egg retrieval.

Cetrorelix acetate: 0.25 mg, administered subcutaneously, starting from the 6th day of stimulation or when the leading follicle reaches 12–14 mm in size, continuing until the day of egg retrieval.

GnRH agonists:

Buserelin: 0.25 mg, once daily, administered subcutaneously, from the stimulation cycle to the day of egg retrieval；Leuprorelin: 0.5 mg, once daily, administered subcutaneously, from the stimulation cycle to the day of egg retrieval; Nafarelin: 0.2 mg, three times daily, administered intranasally, from the stimulation cycle to the day of egg retrieval; Triptorelin/Leuprorelin: 3.75 mg, administered subcutaneously, from the 2nd day of the menstrual cycle to the day of egg retrieval.

#### 4.1.3 Evidence summary

We included a total of 62 RCTs in our analysis. Among these, 34 studies provided relevant data for the NOR population,^1^, ^14^, ^15^, ^16^, ^17^, ^18^, ^19^, ^20^, ^21^, ^22^, ^23^, ^24^, ^25^, ^26^, ^27^, ^28^, ^29^, ^30^, ^31^, ^32^, ^33^, ^34^, ^35^, ^36^, ^37^, ^38^, ^39^, ^40^, ^41^, ^42^, ^43^, ^44^, ^45^, ^46^ 17 for POR population,^47^, ^48^, ^49^, ^50^, ^51^, ^52^, ^53^, ^54^, ^55^, ^56^, ^57^, ^58^, ^59^, ^60^, ^61^, ^62^, ^63^ and 11 for HOR population.^64^, ^65^, ^66^, ^67^, ^68^, ^69^, ^70^, ^71^, ^72^, ^73^, ^74^

In the population with NOR, using GnRH antagonists compared to agonists is likely to reduce the incidence of moderate to severe ovarian hyperstimulation syndrome (OHSS) (RR = 0.65, 95% CI 0.32–1.30, moderate certainty evidence, Figure S1.3). However, this benefit is offset by several harms, including reduction in live birth rate (Figure S1.1), cumulative live birth rate (Figure S1.2), clinical pregnancy rate (Figure S1.4), and the implantation rate (Figure S1.5). Furthermore, the number of M II oocytes retrieved is fewer, and the endometrial thickness may be decreased. (Figure S1.6–1.7). The overall certainty of evidence for these outcomes is low (SoF table 1.1).

In people with POR, relative to agonists, the benefit of using GnRH antagonists is a potential increase in the cumulative live birth rate (RR = 1.08, 95% CI 0.60–1.95, moderate certainty). However, the harms include a probable reduction in the clinical pregnancy rate (RR = 0.89, 95% CI 0.71–1.11, moderate certainty) and implantation rate (RR = 0.93, 95% CI 0.71–1.22, moderate certainty). The overall difference in benefits and harms is minimal, and certainty of evidence is low (SoF table 1.2).

In people with HOR, the relative benefits include a probable reduction in the incidence of moderate to severe OHSS (RR = 0.48, 95% CI 0.27–0.86, high certainty), a potentially increased clinical pregnancy rate (RR = 1.01, 95% CI 0.87–1.17, low certainty), and number of M II oocytes retrieved (Figure S1.6). However, we may see a reduction in the live birth rate (Figure S1.1), implantation rate (Figure S1.5), a probable decrease of endometrial thickness (Figure S1.7), and miscarriage rate (Figure S1.8). The certainty of evidence ranges from high to low, and the overall certainty is low (SoF table 1.3).

#### 4.1.4 Rational for recommendation

GnRH antagonists slightly reduce moderate to severe OHSS in people with NOR but show no significant benefit or difference in other critically important outcomes. The GDG believed the harms outweigh the benefits for this group. For people with POR, antagonists slightly increase cumulative live birth rates, but evidence is of low certainty. Considering very poor ovarian function patients can't use agonist schemes, the GDG suggested non‐preferential use of antagonists. For people with HOR, antagonists significantly reduce OHSS, slightly increase clinical pregnancy rates, and MII oocytes. Although more resources may be needed, there's no economic evidence showing significant differences between antagonists and agonists. Please refer to evidence for further details of other considerations (EtD Table 1). The GDG conditionally recommend antagonists for this group.

### 4.2 PICO 2. Should pretreatment be recommended before implementing the antagonist protocol in people undergoing COS with GnRH antagonist treatment in IVF?

#### 4.2.1 Recommendation 2

For people with NOR, the GDG does not suggest prioritizing Pretreatment (conditional recommendation, very low‐certainty evidence). Clinicians may adjust clinical practice based on patient conditions.

For people with POR, the GDG tentatively suggests Pretreatment (conditional recommendation, low‐certainty evidence). Clinicians may adjust clinical practice based on patient conditions and preferences.

#### 4.2.2 Implementation suggestions

Pretreatment methods may include the following:

1. Oral Contraceptives: Ethinylestradiol/Levonorgestrel containing 30 μg Ethinylestradiol and 150 μg Levonorgestrel; taken from day 2–3 of the cycle for a variable duration of 10–28 days.
2. Estrogens:
  ‐ Estradiol/estradiol valerate, with a daily dose of 2×2 mg (2 mg in the morning and 2 mg in the evening), taken orally from day 25 of the menstrual cycle for 6–10 days.
  ‐ Estraderm TTS 100, releasing 100 mg/d of estradiol, applied twice weekly.
  ‐ 17β‐Estradiol, 4 mg/d, from day 20 of the same cycle to day 2 of the next cycle.
3. Antagonists: 0.25 mg Cetrorelix, starting from day 2 of the cycle for 3–7 days.
4. Androgens:
  ‐ hCG/anastrozole, with 1250 IU hCG subcutaneous injections and 1 mg anastrozole orally, three times daily.
  ‐ Transdermal testosterone gel, 50 mg/d for 4 weeks; or 12.5 mg TTG (Testosterone gel 1%), with a standard testosterone dose of 1.25 mg/d; applied once daily from the sixth day of E‐P pretreatment for 21 days.
  ‐ Dehydroepiandrosterone: 25 mg orally, three times daily for 3 months before IVF/ICSI.
5. Progestins: Micronized progesterone/dydrogesterone, 10 mg/d, from day 15 of the cycle for 10–15 days.

#### 4.2.3 Evidence summary

We included 1 systematic review (SR) and 22 additional RCTs. In the fresh embryo transfer cycles, nine studies used oral contraceptives (OCs) as pretreatment,^33^, ^63^, ^75^, ^76^, ^77^, ^78^, ^79^, ^80^, ^81^ two focused on POR patients and seven on NOR patients. Five studies used estrogen as pretreatment all involving NOR patients.^82^, ^83^, ^84^, ^85^, ^86^ Seven studies used antagonists as pretreatment,^87^, ^88^, ^89^, ^90^, ^91^, ^92^, ^93^ four of these focused on POR patients, two on NOR patients, and one on HOR patients. Lastly four studies used androgens as pretreatment,^94^, ^95^, ^96^, ^97^ three focused on POR patients and one on NOR patients.

In people with NOR, the relative benefit of pretreatment with OCs appears to be an increase in the number of MII oocytes (MD = 1.56, 95% CI 0.14–2.99, Figure S2.1.1), while simultaneously reducing the incidence of moderate OHSS (RR = 0.77, 95% CI 0.13–4.70, Figure S2.1.2). For all other critical outcomes, the harm appears greater than gain (Figure S2.1.3–2.1.7). Similarly, pretreatment with estrogen only brings benefit to the number of MII oocytes (MD = 0.53, 95% CI −0.23–1.29, Figure S2.2.1), and there is no evidence of benefit to any other outcomes (Figure S2.2.2–2.2.7). The benefit of using antagonists as pretreatment is potentially an increase in implantation rate (RR = 1.01, 95% CI 0.75–1.36, Figure S2.3.1); reduction in moderate to severe OHSS (RR = 0.50, 95% CI 0.05–5.39, Figure S2.3.2), but there is no other apparent benefit in other critical outcomes (Figure S2.3.3–2.3.7). The overall certainty of evidence on GnRH‐ant is very low, while for the other pretreatments, they are low (SoF table 2.1).

In people with POR, the relative benefit of pretreatment with OCs appears to be an increase in live birth rate (RR = 1.54, 95% CI 0.84–2.83), implantation rate (RR = 1.57, 95% CI 0.90–2.74), and clinical pregnancy rate (RR = 1.53, 95% CI 0.88–2.67), and reduction in miscarriage rate (RR = 0.97, 95% CI 0.18–5.26). Similarly with Estrogen pretreatment, the implantation rate (RR = 2.06, 95% CI 0.54–7.83), and endometrial thickness may be improved (MD = 0.82, 95% CI 0.50–1.14), the number of MII oocytes may be increased (MD = 0.65, 95% CI −0.46–1.75), as well as clinical pregnancy rate (RR = 2.68, 95% CI 1.41–5.09), while the miscarriage rate may decrease (RR = 0.90, 95% CI 0.30–2.70). Similar direction of benefit is also observed with the antagonists as pretreatment group (Figure S2.1–2.5). The GDG concluded that the benefits of using antagonists for pretreatment outweigh the minimal risks. The overall certainty of the above evidence is low (SoF table 2.2).

The benefit in people with HOR including, a potential increase in implantation rate (MD = 8.7, 95% CI −1.69–19.09), M II oocyte retrievals (MD = 0.55, 95% CI −3.04–4.14), clinical pregnancy rate (RR = 4.61, 95% CI 1.01–20.93), and decrease in miscarriage rate (RR = 0.86, 95% CI 0.17–4.37). There is no clear benefit on any other pre‐specified outcomes (Figure S2.1–2.5, SoF table 2.3).

#### 4.2.4 Rationale for recommendation

For NOR patients, evidence shows pretreatment causes more harms than benefits. Given minimal clinical benefits, the GDG believed factors like resources, feasibility, and equity won't affect guideline recommendations. For POR patients, the benefits of pretreatment may outweigh the harms, but due to low evidence certainty, the recommendation is conditional. Resource differences for pretreatment in China are likely small and should not impact equity, acceptability, or feasibility (EtD table 2). For HOR patients, small sample sizes and low‐certainty evidence not aligning with clinical practice led the GDG to refrain from making recommendations.

### 4.3 PICO 3. Should fixed regimen or flexible regimen be preferred under GnRH‐ant protocol for people undergoing COS?

#### 4.3.1 Recommendation 3

- For people with NOR, the GDG tentatively suggests the fixed antagonist protocol over flexible protocol, with the provision that adjustment is considered following discussion and full consideration of the individuals' specific situation (conditional recommendation, low‐certainty evidence).
- For people with HOR, there is insufficient evidence to either support or refute the use of fixed antagonist protocol compared to the flexible protocol. The GDG suggests collaborative decisions with the patients, exercising caution, and with full consideration of each individual's specific situation (Conditional recommendation for either the intervention or the comparison, very low‐certainty evidence).

#### 4.3.2 Implementation suggestion

- Fixed Protocol: Start administering the antagonist at 0.25 mg daily from the 5th–6th day of ovarian stimulation.
- Flexible Protocol: Administer the antagonist when at least one follicle reaches a diameter of ≥14 mm, each dominant follicle has a serum E_2_ level of ≥200 pg/mL, and LH is ≥10 IU/L.

#### 4.3.3 Evidence summary

We included 9 RCTs under this PICO,^98^, ^99^, ^100^, ^101^, ^102^, ^103^, ^104^, ^105^, ^106^ among which 1 RCT is conducted with HOR population, and 8 RCTs with NOR population. There is a lack of evidence in POR population.

In people with NOR, the fixed protocol offers several benefits over the flexible protocol. It may increase the cumulative live birth rate (RR = 1.04, 95% CI 0.89–1.21), implantation rate (RR = 1.03, 95% CI 0.68–1.57), clinical pregnancy rate (RR = 1.16, 95% CI 0.89–1.51), and it may reduce early miscarriage (RR = 0.50, 95% CI 0.09–2.65). However, it also may increase moderate OHSS (RR = 1.26, 95% CI 0.35–4.57), it reduces MII oocytes (MD = −0.08, 95% CI −0.77–0.60), and decreases endometrial thickness (MD = −0.30, 95% CI −0.85–0.25) (Figure S3.1–3.4). The GDG concluded that the benefits of the fixed protocol have the potential to outweigh the harms compared to the flexible protocol.

The certainty of evidence among the studies conducted in NOR population is low, and among the HOR population is very low (SoF table 3).

#### 4.3.4 Rational for recommendation

For people with NOR, evidence shows that the benefits of a fixed protocol may outweigh the harms. For people with HOR, the absence of critical outcomes like OHSS leads to high uncertainty. Considering resources, the early addition of an antagonist could prolong stimulation (by 2–3 days), increasing the usage of antagonists and gonadotropins. Therefore, the resource requirements for high responders are relatively higher. In summary, the GDG suggested cautious consideration or non‐use of the fixed protocol (EtD table 3).

### 4.4 PICO 4. Should early initiation of GnRH‐ant be recommended during stimulation for people undergoing COS?

#### 4.4.1 Recommendation 4

- For people with varying ovarian responses, the GDG notes there is insufficient evidence to demonstrate the advantage of early initiation of GnRH‐ant, and therefore, refrains from making a recommendation (Conditional recommendation for either the intervention or the comparison, very low‐certainty evidence). Early initiation of the GnRH‐ant protocol should only be considered if the conventional protocol has failed, and this decision should be made with reference to follicle size consistency. Clinicians are encouraged to adjust practice based on the specific circumstances of the individual.

#### 4.4.2 Implementation suggestions

- Early initiation: Defined as starting the antagonist from day 1 or 2 of the stimulation cycle; follicle diameter less than 12 mm; E2 ≤ 200 pg/ml; and the antagonist is started before day 5 of stimulation (excluding day 5).
- Late initiation: GnRH antagonist is started on day 6 of stimulation, which is the conventional start of the fixed protocol.

#### 4.4.3 Evidence summary

We included 6 RCTs under this PICO question.^102^, ^107^, ^108^, ^109^, ^110^, ^111^ The overall certainty of evidence under this PICO is very low (SoF table 4).

In people with NORearly initiation shows probable benefits such as a 99 per 1000 increase in clinical pregnancy rate (RR = 1.33, 95% CI 0.66–2.69, Figure S4.1) and a 1.11% increase in implantation rate (Figure S4.2, 4.3). However, it may also reduce the live birth rate by 26 per 1000 (Figure S4.4). The GDG concluded that, due to limited evidence, the benefits and harms of early initiation compared to late initiation remain uncertain.

In people with POR early initiation may increase the embryo implantation rate (RR = 0.25, 95% CI 0.13–0.37) and the number of oocytes retrieved (MD = 0.97, 95% CI −0.01–1.95). However, it may also reduce the number of MII oocytes (MD = −0.89, 95% CI 0.02–1.76) and high‐certainty embryos (MD = −0.31, 95% CI −0.24–0.86) (Figure S4.1–4.8). The GDG is uncertain if early initiation offers more benefits than harms compared to late initiation, as the evidence is of very low certainty.

In people with HOR early initiation may increase live birth rate (RR = 1.25, 95% CI 0.44–3.55), clinical pregnancy rate (RR = 1.21, 95% CI 0.93–1.58), endometrial thickness (MD = 0.20, 95% CI −0.49–0.89), oocytes retrieved (MD = 2.20, 95% CI −0.21–4.61), and implantation rate (RR = 2.00, 95% CI 0.45–8.94), Figure S4.1–4.8. There is a lack of evidence of harm. The GDG has limited confidence in the observed effect due to the small sample size and missing outcomes such as OHSS, hence conditional recommendation is made for either the intervention or the comparison.

Overall, for people with varying ovarian responses, early initiation of antagonists shows more benefits than harms compared to late initiation. However, due to very low evidence certainty, rare clinical use, and increased resource consumption, the GDG advises cautious application of early initiation (EtD table 4). If conventional protocols fail, early initiation can be considered, while ensuring follicle size consistency. The relative effectiveness of early versus late initiation appears consistent across different ovarian response groups, albeit the certainty of evidence is very low.

### 4.5 PICO 5. Should the GnRHa add‐on trigger be recommended over the HCG trigger alone for people undergoing COS?

#### 4.5.1 Recommendation 5

- For people with normal ovarian response, the GDG tentatively suggests adding GnRHa trigger compared to using HCG trigger alone (conditional recommendation, very low‐certainty evidence). Clinicians may adjust practice based on individual preferences and previous assisted reproduction outcomes.
- For people with normal ovarian response, the GDG recommends against using GnRHa trigger alone compared to using HCG trigger alone. (strong recommendation, very low‐certainty evidence).

#### 4.5.2 Implementation suggestion

The reported dosage of GnRHa as a trigger in the literature is 0.2 mg. The conventional dosage of HCG is 10,000 IU.

#### 4.5.3 Evidence summary

Eleven studies compared adding GnRHa trigger to HCG by using HCG alone in people with NOR.^112^, ^113^, ^114^, ^115^, ^116^, ^117^, ^118^, ^119^, ^120^, ^121^, ^122^ Very low certainty evidence indicates the benefits of using a GnRH‐a trigger with HCG including probable increase in: live birth rates (RR = 1.37, 95% CI 0.97–1.95), cumulative live birth rates (RR = 1.39, 95% CI 0.92–2.11), implantation rates (RR = 1.25, 95% CI 0.98–1.61), clinical pregnancy rates (RR = 1.20, 95% CI 1.03–1.39), and the number of MII oocytes (MD = 0.82, 95% CI 0.20–1.44). It may also reduce miscarriage rates (RR = 0.73, 95% CI 0.37–1.42) (Figure S5.1.1 and 5.1.7). The only harm noted was a slight reduction in endometrial thickness (MD = −0.04, 95% CI −0.48–0.39). Overall, the GDG suggests the current evidence may indicate more benefits than harms, albeit the certainty of evidence under this comparison is very low (SoF table 5).

In people with POR,^123^ the intervention may increase clinical pregnancy rate (RR = 1.86 95% CI 0.83–4.18) and reduce miscarriage rate (RR = 0.56 95% CI 0.20–1.52). There appears to be no obvious benefit of the intervention in people with HOR.

When comparing GnRHa and HCG alone, in people with NOR,^121^, ^124^, ^125^, ^126^, ^127^, ^128^ the number of MII oocytes may increase (MD = 0.35, 95% CI −0.19–0.88, Figure S5.1.5), and endometrial thickness may increase (MD = 0.01, 95% CI 0.00–0.02). The potential harm is reflected in reduced implantation rate (Figure S5.1.3), a reduced clinical pregnancy (Figure S5.1.4), and an increased miscarriage rate (Figure S5.1.6). Overall, the harm of using GnRHa alone in people with NOR probably outweighs benefits (very low evidence, SoF table 5). The evidence in HOR patients is lacking.

#### 4.5.4 Rational for recommendation

In women with NOR, using an additional GnRH‐a trigger might increase live birth rates, cumulative live birth rates, implantation rates, clinical pregnancy rates, and the number of MII oocytes, while possibly reducing miscarriage rates. However, the evidence certainty is very low, and the results remain highly ambiguous. Considering the slight increase in GnRHa resource usage, clinical experts recommend this approach with caution (EtD table 5).

For women with NOR, replacing the HCG trigger with a GnRH‐a trigger significantly reduces implantation rates and clinical pregnancy rates while increasing miscarriage rates. Although there is a slight improvement in live birth rates, the evidence certainty is extremely low, making the results variable and inconsistent with other outcomes. Given the higher cost of the GnRHa trigger compared to the HCG trigger, experts unanimously do not recommend using the GnRHa trigger as a replacement for the HCG trigger. Strong recommendations are made against using the GnRHa trigger to avoid misleading clinical practice. For other populations, the evidence is either highly uncertain or lacking, preventing clear recommendations (EtD table 5).

### 4.6 PICO 6. Should reinforced luteal support be recommended for individuals undergoing fresh embryo transfer with GnRHa trigger?

#### 4.6.1 Recommendation 6

- Regarding the need for enhanced luteal support after using the GnRH agonist trigger in antagonist protocol, there is a lack of evidence, and the GDG makes conditional recommendation for either the intervention or the comparison on enhanced luteal support (conditional recommendation for either the intervention or the comparison, very low‐certainty evidence).
- For people with HOR, the GDG suggests immediate luteal support after oocyte retrieval in fresh cycles with GnRH agonist trigger compared to starting luteal support 2–5 days after oocyte retrieval, to improve pregnancy success rates and reduce miscarriage rates (conditional recommendation, very low‐certainty evidence).

#### 4.6.2 Implementation suggestion

The luteal support medications used in the included study were intramuscular progesterone injections (100 mg daily) and vaginal progesterone suppositories (400 mg, twice daily), as well as vaginal progesterone gel (90 mg), administered immediately after oocyte retrieval. The control group started luteal support 2–5 days after oocyte retrieval.

#### 4.6.3 Evidence summary

There lacks evidence regarding enhanced luteal support after an antagonist protocol with an agonist trigger, so GDG cannot provide recommendations. For people with HOR, immediate luteal support post‐retrieval may increase ongoing pregnancy rates (RR = 1.98, 95% CI 1.16–3.37) and reduce early miscarriage rates (RR = 0.36, 95% CI 0.12–1.06). The intervention appears to have slightly lowered the number of oocytes retrieved (MD = −1.70, 95% CI −6.42–3.02) (Figure S6.1–6.3), but is unrelated to luteal support timing. The overall certainty of evidence is very low (SoF table 6).

For HOR patients, the benefits of immediate luteal support outweigh the harms.

#### 4.6.4 Rational for recommendation

In HOR patients, initiating luteal support immediately after oocyte retrieval compared to starting 2–5 days post‐retrieval increases ongoing pregnancy rates and decreases early miscarriage rates, indicating more benefit than harm. However, the low certainty of evidence, derived from observational study, along with the potential increase in progesterone usage and unclear economic benefits, leads the GDG to conditionally recommend immediate luteal support (EtD table 6). Clinicians should adjust their practice based on patient preferences and clinical situations.

### 4.7 PICO 7. Should fresh or frozen embryo transfer be recommended in individuals receiving COS under GnRH‐ant protocol?

#### 4.7.1 Recommendations

- For people with HOR undergoing COS with the GnRH antagonist protocol, the GDG recommends freeze‐all regimen and cancellation of fresh embryo transfer (strong recommendation, very low‐certainty evidence). In the subgroup of single blastocyst transfer, while benefit of fresh single blastocyst transfer is unclear, the harm is significant (reduced live birth and clinical pregnancy rates), with a tendency towards frozen‐thawed single blastocyst transfer. In the subgroup of multiple cleavage stage embryo transfers, the clinical benefit of fresh embryo transfer is significant (reduced risk of pre‐eclampsia and pregnancy‐induced hypertension). However, the disadvantages are also evident (increased moderate to severe OHSS, reduced live birth and clinical pregnancy rates). Clinicians may adjust clinical practice based on individual preferences and previous assisted reproduction outcomes.
- For people with normal ovarian response undergoing COS with the GnRH antagonist protocol, the GDG tentatively suggests freeze‐all regimen (conditional recommendation, low‐certainty evidence). In the subgroup of single blastocyst transfer, the benefit of fresh embryo transfer is moderate (reduced risk of pre‐eclampsia), but as the disadvantages are significant (greatly reduced implantation and clinical pregnancy rates), there may be a preference for frozen embryo transfer. In the subgroup of double/multiple cleavage stage transfers, the benefit of fresh embryo transfer is moderate (increased live birth, clinical pregnancy, implantation rates, and reduced risk of pre‐eclampsia), accompanied by moderate disadvantages (increased moderate to severe OHSS). Overall, there may be a preference for frozen embryo transfer. Clinicians may adjust clinical practice based on patient preferences and previous assisted reproduction outcomes.

#### 4.7.2 Evidence summary

In people with HOR,^129^, ^130^, ^131^ receiving COS antagonist treatment for IVF, evidence shows fresh transfer increases OHSS risk (RR = 3.45, 95% CI 0.80–14.80) and decreases live birth (RR = 0.87, 95% CI 0.78–0.96) and clinical pregnancy rates (RR = 0.96, 95% CI 0.88–1.04). In blastocyst single embryo transfer, fresh transfer reduces live birth (RR = 0.95, 95% CI 0.68–1.32) and clinical pregnancy rates (RR = 0.89, 95% CI 0.68–1.15), with a potential OHSS risk increase. For cleavage‐stage multiple embryo transfer, fresh transfer reduces pre‐eclampsia (RR = 0.32, 95% CI 0.13–0.80) and pregnancy‐induced hypertension (RR = 0.51, 95% CI 0.18–1.47), but increases OHSS (RR = 2.43, 95% CI 0.48–12.34), and reduces live birth (RR = 0.86, 95% CI 0.77–0.96) and clinical pregnancy rates (RR = 0.96, 95% CI 0.88–1.05) (Figure S7.1.1–7.1.5).

In people with NOR, fresh embryo transfer compared to frozen embryo transfer, moderately reduces the risk of pre‐eclampsia (RR = 0.64, 95% CI 0.37–1.11) and slightly reduces pregnancy‐induced hypertension (RR = 0.96, 95% CI 0.50–1.85). However, it slightly increases the risk of OHSS (RR = 2.80, 95% CI 1.41–5.56), moderately reduces live birth rates (RR = 0.92, 95% CI 0.87–0.98), as well as embryo implantation rates (RR = 0.90, 95% CI 0.68–1.18), and clinical pregnancy rates (RR = 0.94, 95% CI 0.73–1.21). Overall, while fresh embryo transfer may offer some benefits in reducing specific pregnancy‐related complications, it also presents significant risks, particularly concerning OHSS and reduced success rates in terms of live births and pregnancies (Figure S7.2.1–7.2.6).

The certainty of above evidence is low (SoF table 7).

#### 4.7.3 Rational for recommendation

In HOR patients undergoing COS with the antagonist protocol in IVF, the benefits of fresh embryo transfer versus full freezing are unclear for critical outcomes. Fresh embryo transfer increased the risk of OHSS and decreased live birth and clinical pregnancy rates. The balance favors full freezing due to these risks. Since there is no reporting of harm from embryo freezing, clinicians should adjust their approach based on individual patient conditions. Subgroup analysis indicated that in HOR patients receiving blastocyst single embryo transfer, fresh transfer shows unclear benefits and significant harm (lower live birth and clinical pregnancy rates), results leaning favorably towards frozen transfer. In cleavage stage multiple embryo transfers, fresh transfer showed benefits (reduced pre‐eclampsia and gestational hypertension) but also significant harm (increased moderate‐to‐severe OHSS, lower live birth, and clinical pregnancy rates), suggesting a preference for frozen transfer. Without evidence comparing single blastocyst and other transfer strategies (e.g., double cleavage stage or repeated transfers) in frozen cycles, the GDG cannot recommend specific strategies for frozen embryo transfer.

In NOR patients undergoing IVF with a GnRH antagonist protocol, fresh embryo transfer compared to full freezing reduces pre‐eclampsia but increases OHSS, lowers live birth, implantation, and clinical pregnancy rates, and slightly raises preterm birth rates. The GDG concluded that overall outcomes might favor full freeze transfers. Clinicians need to make adjustments based on patient circumstances since there is the lack of evidence of potential harm from embryo freezing. For single blastocyst transfer, fresh embryo transfer moderately reduces pre‐eclampsia but significantly lowers implantation and clinical pregnancy rates. Overall, the effect favors full freezing transfer. For multiple cleavage‐stage embryo transfers, fresh transfer moderately increases live birth, clinical pregnancy, and implantation rates, reduces pre‐eclampsia, but moderately increases moderate‐severe OHSS, also favoring full freezing transfer. Due to the lack of evidence comparing single blastocyst freeze‐all cycles to other strategies (double cleavage‐stage or staged transfers), the GDG could not recommend a specific freeze‐all embryo transfer strategy.

### 4.8 PICO 8. Should day 3 or day 5 be recommended as the preferred timing to perform the embryo transfer in people undergoing COS?

#### 4.8.1 Recommendation 8

- For people undergoing COS with the GnRH antagonist protocol, there is limited evidence regarding the choice between D3 and D5 embryo transfer. After thorough discussion, the GDG unanimously agreed not to make preferential recommendation between D3 and D5 single embryo transfer (Conditional recommendation for either the intervention or the comparison, moderate‐certainty evidence).

#### 4.8.2 Evidence summary

In IVF patients undergoing COS with an antagonist protocol,^132^ the benefits of D3 embryo transfer compared to D5 embryo transfer are unknown. However, D3 embryo transfer appears to reduce the clinical pregnancy rate by 99 per 1000 people (RCT = 1, *n* = 351, RR = 0.70, 95% CI 0.50–0.99) and slightly increases the risk of ectopic pregnancy by 3 per 1000 people (RCT = 1, *n* = 132, RR = 1.24, 95% CI 0.08–19.36) (Figure S8.1–8.3). The certainty of evidence is moderate (SoF table 8). The multiple pregnancy rate is unclear. The evidence is limited and the benefits and harms of embryo transfer on day 3 (D3) compared to day 5 (D5) are unclear, so conditional recommendation for either the intervention or the comparison are made at this time (EtD table 8).

## 5 DISCUSSION

We developed the Clinical Practice Guidelines (CPG) using rigorous methods to establish the usage and management of GnRH antagonists in ovarian stimulation for assisted reproductive technology. Evidence shows the clinical effects of GnRH antagonists are comparable to GnRH agonists. Considering the impact on critical outcomes, especially reducing moderate to severe OHSS in high responders, the GDG supports their use in this group. The CPG also analyzed full freezing embryo transfer versus fresh embryo transfer, finding significant benefits for full freezing, including reduced OHSS, increased live birth rates, and clinical pregnancy rates. Therefore, the GDG suggests considering full freezing embryo transfer.

The recommendations of this guideline are more specific compared to the 2020 European Society of Human Reproduction and Embryology (ESHRE) guidelines,^133^ particularly in evaluating the clinical efficacy for different ovarian response populations. Unlike the ESHRE guideline, which generally recommends GnRH antagonists, our guideline provides detailed assessments of benefits and harms, finding significant benefits in high ovarian responders, leading to a clear recommendation.

Developed in strict accordance with international standards, this guideline is based on comprehensive evidence and thorough discussions, resulting in recommendations suitable for China. The evidence analysis also offers a reference for international clinical practice. Future research should address the gaps remaining in evidence for most clinical issues, especially those regarding economic benefits and patient preferences, to support improved outcomes in assisted reproductive technology.

## AUTHOR CONTRIBUTIONS

**Rong Li:** Conceptualization; funding acquisition; resources; supervision; validation; writing original draft. **Rui Yang:** Data curation; formal anlaysis; project administration; validation; writing original draft. **Junhao Yan:** Data curation; validation; review and editing manuscript. **Yang Song:** Methodology. **Yunxia Cao:** review and editing manuscript. **Zijiang Chen:** Review and editing manuscript. **Chenchen Xu:** Data curation; formal anlaysis. **Zhan Zhao:** Data curation; formal anlaysis. **Yichun Guan:** Review and editing manuscript. **Fei Gong:** Review and editing manuscript. **Guimin Hao:** Review and editing manuscript. **Hefeng Huang:** Review and editing manuscript. **Li Jin:** Review and editing manuscript. **Fenghua Liu:** Review and editing manuscript. **Jiayin Liu:** Review and editing manuscript. **Xiaoyan Liang:** Review and editing manuscript. **Xiaolin La:** Review and editing manuscript. **Yun Sun:** Review and editing manuscript. **Xiaohong Wang:** Review and editing manuscript. **Yanwen Xu:** Review and editing manuscript. **Cuilian Zhang:** Review and editing manuscript. **Jie Qiao**: Conceptualisation, Review and editing.

## CONFLICT OF INTEREST STATEMENT

The management of conflicts of interest (COIs) for members of the guideline working group and the GDG, including clinical experts and methodologists, follows the principles outlined by the World Health Organization (WHO) and the Guidelines International Network (GIN). Members of the guideline working group and the GDG disclosed their potential COIs to the guideline commissioner, including any financial, intellectual property, or other interests related to the guideline recommendations. Financial interests included employment, consultancy roles, research funding, or investments in entities connected to the project. Intellectual property interests, such as patents, trademarks, or affiliations with competing products or organizations, also had to be declared. Additionally, any other potential conflicts, such as work with competitors, third‐party support (e.g., travel expenses), and payments for public speaking, likely to influence judgment, needed to be disclosed. None of the working group members, nor the GDG members had any substantial COIs; they were therefore approved to participate fully in the guideline development process.

## ETHICS STATEMENT

This clinical practice guideline was developed in alignment with globally accepted standards for evidence synthesis and guideline formulation. The authors confirmed, through a standardized disclosure procedure, that no competing interests influenced the work. The recommendations derive from a rigorous analysis of publicly accessible evidence, with no direct involvement of human or animal participants. The process respected principles of equity, transparency, and accountability.

## Supporting information

Appendix 1 Clinical Specialty.

Appendix 2 Clinical Questions and PICOs.

Appendix 3 Search strategy.

Appendix 4 PRISMA flow diagram.

Appendix 5 Meta analysis forest plots.

Appendix 6 Summary of findings tables‐clean.

Appendix 7 Evidence to decision clean.

## Data Availability

The data that support the findings of this study are available from the corresponding author upon reasonable request.
