# Supplementary material for: Recommendations for the management of GnRH‐ant for ovarian stimulation in the assistant reproductive process: Benefits and harms for different ovarian response population
Source: Clin Public Health Guidel. 2025 May 21;2(3):e70022. doi: 10.1002/gin2.70022 (PMC13563367; doi:10.1002/gin2.70022)
Supplement: Supplementary file 1 [file GIN2-2-e70022-s002.docx]

**Table of Contents**

[PICO 1 Whether the GnRH-ant protocol is recommended over the GnRH agonist (GnRH-a) protocols?	2](#_Toc172041855)

[PICO 2 Among patients undergoing IVF assisted reproduction with COS using GnRH-ant, is it recommended to implement pretreatments?	14](#_Toc172041856)

[PICO 3 Should fixed regimen or flexible regimen be preferred under GnRH-ant protocol?	36](#_Toc172041857)

[PICO 4 Is early initiation of GnRH-ant during stimulation recommended?	44](#_Toc172041858)

[PICO 5 Is the GnRHa add-on trigger recommended over the HCG trigger alone?	54](#_Toc172041859)

[PICO 6 For individuals undergoing fresh embryo transfer with GnRHa trigger, is reinforced luteal support recommended?	64](#_Toc172041860)

[PICO 7 Fresh embryo transfer compared with frozen-all strategy for GnRH-ant protocol?	72](#_Toc172041861)

[PICO 8 Which is the preferred timing to perform the embryo transfer: Day 3 or Day 5?	87](#_Toc172041862)

# PICO 1 Whether the GnRH-ant protocol is recommended over the GnRH agonist (GnRH-a) protocols?

## **Table 1.** **Recommedations**

| Problem  Is the problem a priority? | | |
| --- | --- | --- |
| Judgement | Research evidence | Additional considerations |
| ○ No ○ Probably no ○ Probably yes ● Yes ○ Varies ○ Don't know | GnRH antagonists were introduced into the protocols for ovarian stimulation in assisted reproductive technology (ART) in the 1990s. These antagonists reversibly bind to GnRH receptors in the pituitary gland, rapidly suppressing the release of endogenous GnRH. This allows for a quick inhibition of pituitary function within a short time frame (1-2 hours), preventing premature LH surges while maintaining pituitary responsiveness. Due to these advantages, there has been a gradual increase in the clinical demand for GnRH antagonists in assisted reproductive therapies.  In response to these benefits, expert consensus guidelines for the clinical use of GnRH antagonists were established by the Chinese Medical Association's Reproductive Medicine Branch in 2015, and evidence-based guidelines on ovarian stimulation protocols were issued by the European Society of Reproductive Medicine in 2020. However, specific guidance on the use and management of GnRH antagonists in IVF treatment was lacking in existing guidelines. Consequently, the Reproductive Medicine Specialty Committee of the Chinese Medical Doctor Association initiated the development of clinical management guidelines for the use of GnRH antagonists in IVF treatment. These guidelines provide tailored recommendations for different ovarian response populations.  Poor ovarian reserve/response (POR): Currently, the internationally recognized criteria for POR are defined by the Bologna criteria. According to these criteria, a diagnosis of POR requires meeting at least two out of the following three conditions: ① Advanced maternal age (≥ 40 years) or other risk factors for poor ovarian response; ② Previous history of poor ovarian response (≤ 3 oocytes retrieved with conventional stimulation); ③ Abnormal results in ovarian reserve tests such as AFC < 5-7, AMH < 0.5-1.1 ng/ml, or FSH > 8.5 mIU/mL (Kim 2021).  If a patient does not meet the criteria for advanced age or has normal results in ovarian reserve tests, those who experience poor ovarian response in at least two previous maximum stimulation cycles can also be classified as poor responders. Additional criteria identified in literature searches before 2015 include: fewer than 4 mature follicles in the first IVF cycle, FSH levels of 10-15 mIU/ml, and estradiol levels below 1500/600 pg/ml. | |
| Desirable Effects  How substantial are the desirable anticipated effects? | | |
| Judgement | Research evidence | Additional considerations |
| ○ Trivial ● Small ○ Moderate ○ Large ○ Varies ○ Don't know | Table 1: Normal Ovarian Responses Outcomes 1 **Outcomes** \| **№ of participants (studies) Follow-up** \| **Certainty of the evidence (GRADE)** \| **Relative effect (95% CI)** \| **Anticipated absolute effects^*^ (95% CI)** \|; \| \| \| \| **Risk with GnRH-a** \| **Risk difference with GnRH-ant**; Moderate and severe OHSS \| 1298 (7 RCTs) \| ⨁⨁⨁◯ Moderate^a,b,c^ \| **RR 0.65** (0.32 to 1.30) \| Study population \|; \| \| \| \| 81 per 1,000 \| **28 fewer per 1,000** (55 fewer to 24 more) a. Although included studies have a high risk of performance bias, the outcome is unlikely to be affected. b. One of the included studies also has a high/unclear risk of selection bias, but the study is unlikely to affect the final estimates (weight <20%). c. Downgraded by one level due to imprecision: the effect could be both beneficial and harmful. Table 2: Poor Ovarian Responses Outcomes 1 **Outcomes** \| **№ of participants (studies) Follow-up** \| **Certainty of the evidence (GRADE)** \| **Relative effect (95% CI)** \| **Anticipated absolute effects^*^ (95% CI)** \|; \| \| \| \| **Risk with GnRH-a** \| **Risk difference with GnRH-ant**; Cumulative live birth rate \| 191 (1 RCT)^1^ \| ⨁⨁⨁◯ Moderate^a,b^ \| **RR 1.08** (0.60 to 1.95) \| Study population \|; \| \| \| \| 181 per 1,000 \| **14 more per 1,000** (72 fewer to 172 more) a. Although included studies have high risk performance bias, it is unlikely to influence the final outcome. b. Downgraded by one level due to imprecision: low event rate and the effect could be both beneficial and harmful. Table3: High Ovarian Responses Outcomes 3 **Outcomes** \| **№ of participants (studies) Follow-up** \| **Certainty of the evidence (GRADE)** \| **Relative effect (95% CI)** \| **Anticipated absolute effects^*^ (95% CI)** \|; \| \| \| \| **Risk with GnRH-a** \| **Risk difference with GnRH-ant**; Moderate and severe OHSS \| 522 (5 RCTs) \| ⨁⨁⨁⨁ High^a^ \| **RR 0.48** (0.27 to 0.86) \| Study population \|; \| \| \| \| 443 per 1,000 \| **230 fewer per 1,000** (324 fewer to 62 fewer); Clinical pregnancy rates assessed with: Number of events \| 1041 (10 RCTs) \| ⨁⨁◯◯ Low^b,c^ \| **RR 1.01** (0.87 to 1.17) \| Study population \|; \| \| \| \| 436 per 1,000 \| **4 more per 1,000** (57 fewer to 74 more); Metaphase II oocytes assessed with: number of MII oocytes \| 460 (3 RCTs) \| ⨁⨁⨁◯ Moderate^c^ \| - \| The mean metaphase II oocytes was **0** \| MD **0.72 more** (0.91 fewer to 2.36 more) a. Included studies have an unclear risk of bias (weight >20%). However, it is unlikely to affect the overall estimates. b. Downgraded by one level due to inconsistency: the point estimates are in different directions across included studies (weight>20%). I^2^ is low, but we consider it is because of the wide CI and small sample size. c. Downgraded by one level due to imprecision: the effect could be both beneficial and harmful. | |
| Undesirable Effects  How substantial are the undesirable anticipated effects? | | |
| Judgement | Research evidence | Additional considerations |
| ○ Large ○ Moderate ● Small ○ Trivial ○ Varies ○ Don't know | Table 1: Normal Ovarian Responses Outcomes 6 **Outcomes** \| **№ of participants (studies) Follow-up** \| **Certainty of the evidence (GRADE)** \| **Relative effect (95% CI)** \| **Anticipated absolute effects^*^ (95% CI)** \|; \| \| \| \| **Risk with GnRH-a** \| **Risk difference with GnRH-ant**; Live birth rate assessed with: Number of events \| 436 (2 RCTs) \| ⨁⨁⨁◯ Moderate^a,b^ \| **RR 0.93** (0.75 to 1.17) \| Study population \|; \| \| \| \| 422 per 1,000 \| **30 fewer per 1,000** (106 fewer to 72 more); Cumulative live birth rate \| 1596 (2 RCTs) \| ⨁⨁◯◯ Low^a,b,c^ \| **RR 1.00** (0.86 to 1.17) \| Study population \|; \| \| \| \| 465 per 1,000 \| **0 fewer per 1,000** (65 fewer to 79 more); Clinical pregnancy rates assessed with: Number of events \| 7597 (32 RCTs) \| ⨁⨁⨁⨁ High^a,d^ \| **RR 0.92** (0.87 to 0.97) \| Study population \|; \| \| \| \| 360 per 1,000 \| **29 fewer per 1,000** (47 fewer to 11 fewer); Implantation rate assessed with: Number of events \| 3037 (8 RCTs) \| ⨁⨁⨁⨁ High^a,d^ \| **RR 0.80** (0.66 to 0.95) \| Study population \|; \| \| \| \| 216 per 1,000 \| **43 fewer per 1,000** (73 fewer to 11 fewer); Metaphase II oocytes assessed with: number of MII oocytes \| 2066 (12 RCTs) \| ⨁◯◯◯ Very low^a,b,d,e^ \| - \| The mean metaphase II oocytes was **0** \| MD **0.77 fewer** (1.61 fewer to 0.07 more); Endometrial thickness assessed with: mm \| 1953 (11 RCTs) \| ⨁⨁⨁◯ Moderate^a,d,f^ \| - \| The mean endometrial thickness was **0** \| MD **0.35 lower** (0.64 lower to 0.06 lower) a. Although included studies have a high risk of performance bias, the outcome is unlikely to be affected. b. Downgraded by one level due to imprecision: the effect could be both beneficial and harmful. c. Downgraded by one level due to inconsistency: the I^2^ value is high (I^2^=58%). We explored the clinical, methdological and statistical characteristics of the studies, but the unexplained heterogeneity remained. This potentially reduces our certainty on the observed magnitude of effect. d. One of the included studies also has a high/unclear risk of selection bias, but the study is unlikely to affect the final estimates (weight <20%). e. Downgraded by two levels due to inconsistency: the I^2^ value is high (I^2^>80%). We explored the clinical, methdological and statistical characteristics of the studies, but the unexplained heterogeneity remained. This potentially reduces our certainty on the observed magnitude of effect. f. Downgraded by one level due to inconsistency: the I^2^ value is high (I^2^=75%). We explored the clinical, methdological and statistical characteristics of the studies, but the unexplained heterogeneity remained. This potentially reduces our certainty on the observed magnitude of effect. Table 2: Poor Ovarian Responses Outcomes 4 **Outcomes** \| **№ of participants (studies) Follow-up** \| **Certainty of the evidence (GRADE)** \| **Relative effect (95% CI)** \| **Anticipated absolute effects^*^ (95% CI)** \|; \| \| \| \| **Risk with GnRH-a** \| **Risk difference with GnRH-ant**; Live birth rate assessed with: Number of events \| 386 (3 RCTs)^1,2^ \| ⨁⨁◯◯ Low^a,b^ \| **RR 0.73** (0.43 to 1.24) \| Study population \|; \| \| \| \| 137 per 1,000 \| **37 fewer per 1,000** (78 fewer to 33 more); Clinical pregnancy rates assessed with: Number of events \| 2039 (15 RCTs) \| ⨁⨁⨁◯ Moderate^c,d^ \| **RR 0.96** (0.75 to 1.23) \| Study population \|; \| \| \| \| 208 per 1,000 \| **8 fewer per 1,000** (52 fewer to 48 more); Implantation rate assessed with: Number of events \| 1481 (7 RCTs) \| ⨁⨁⨁◯ Moderate^c,d^ \| **RR 0.98** (0.73 to 1.31) \| Study population \|; \| \| \| \| 176 per 1,000 \| **4 fewer per 1,000** (48 fewer to 55 more); Endometrial thickness assessed with: mm \| 1621 (11 RCTs) \| ⨁◯◯◯ Very low^a,e^ \| - \| The mean endometrial thickness was **0** \| MD **0.71 fewer** (1.34 fewer to 0.074 fewer) a. Downgraded by one level due to high/unclear risk of selection bias (weight>20%) b. Downgraded by one level due to imprecision: low event rate and the effect could be both beneficial and harmful. c. Although included studies have high risk performance bias, it is unlikely to influence the final outcome. d. Downgraded by one level due to imprecision: the effect could be both beneficial and harmful. e. Downgraded by one level due to inconsistency: the I^2^ value is high (I^2^=80%). We explored the clinical, methdological and statistical characteristics of the studies, but the unexplained heterogeneity remained. This potentially reduces our certainty on the observed magnitude of effect Table 3: High Ovarian Responses Outcomes 4 **Outcomes** \| **№ of participants (studies) Follow-up** \| **Certainty of the evidence (GRADE)** \| **Relative effect (95% CI)** \| **Anticipated absolute effects^*^ (95% CI)** \|; \| \| \| \| **Risk with GnRH-a** \| **Risk difference with GnRH-ant**; Live birth rate assessed with: Number of events \| 208 (1 RCT)^1^ \| ⨁⨁⨁◯ Moderate^a^ \| **RR 0.98** (0.68 to 1.43) \| Study population \|; \| \| \| \| 350 per 1,000 \| **7 fewer per 1,000** (112 fewer to 150 more); Implantation rate assessed with: Number of events \| 395 (2 RCTs) \| ⨁⨁⨁◯ Moderate^b^ \| **RR 0.93** (0.66 to 1.29) \| Study population \|; \| \| \| \| 270 per 1,000 \| **19 fewer per 1,000** (92 fewer to 78 more); Endometrial thickness assessed with: mm \| 723 (5 RCTs) \| ⨁⨁◯◯ Low^b,c^ \| - \| The mean endometrial thickness was **0** \| MD **0.57 lower** (1.19 lower to 0.05 higher); Miscarriage rate assessed with: number of events \| 447 (5 RCTs) \| ⨁◯◯◯ Very low^b,d,e^ \| **RR 1.02** (0.64 to 1.63) \| Study population \|; \| \| \| \| 123 per 1,000 \| **2 more per 1,000** (44 fewer to 77 more) a. Downgraded by two levels due to imprecision: low event rate and the effect could be both beneficial and harmful. b. Downgraded by one level due to imprecision: the effect could be both beneficial and harmful. c. Downgraded by one level due to inconsistency:the I^2^ value is high (I^2^=70%). We explored the clinical, methdological and statistical characteristics of the studies, but the unexplained heterogeneity remained. This potentially reduces our certainty on the observed magnitude of effect. d. Downgraded by one level due to unclear risk of selection bias (weight>20%). e. Downgraded by one level due to inconsistency: the point estimates are in different directions across included studies (weight>20%). I^2^ is low, but we consider it is because of the wide CI and small sample size. | |
| Certainty of evidence  What is the overall certainty of the evidence of effects? | | |
| Judgement | Research evidence | Additional considerations |
| ○ Very low ● Low ○ Moderate ○ High ○ No included studies | See the evidence table.  The certainty of evidence of the critical outcomes are a mixture of high, moderate and low certainty. The overall certainty of the body of evidence under this PICO question is low. | |
| Values  Is there important uncertainty about or variability in how much people value the main outcomes? | | |
| Judgement | Research evidence | Additional considerations |
| ○ Important uncertainty or variability ○ Possibly important uncertainty or variability ● Probably no important uncertainty or variability ○ No important uncertainty or variability | No relevant patient values were identified. The importance of outcome measures in this guideline was determined through voting and discussion by the clinical expert group, considering the clinical significance of outcomes. | |
| Balance of effects  Does the balance between desirable and undesirable effects favor the intervention or the comparison? | | |
| Judgement | Research evidence | Additional considerations |
| ○ Favors the comparison ○ Probably favors the comparison ○ Does not favor either the intervention or the comparison ○ Probably favors the intervention ○ Favors the intervention ● Varies ○ Don't know | Summary of desirable and undesirable effects:  In the population with NOR, using GnRH antagonists compared to agonists can probably reduce the incidence of moderate to high-risk ovarian hyperstimulation syndrome (OHSS), with 28 fewer cases per 1000 people (RCT=7, n=1298, RR=0.65, 95% CI 0.32–1.30). However, this benefit is offset by several harms. There may be a lower live birth rate, with 30 fewer cases per 1000 people (RCT=2, n=436, RR=0.93, 95% CI 0.75–1.17), and a reduced cumulative live birth rate (RCT=2, n=1596, RR=1.00, 95% CI 0.86–1.17). Additionally, the clinical pregnancy rate is probably lower, with 29 fewer cases per 1000 people (RCT=32, n=7597, RR=0.92, 95% CI 0.87–0.97), and the implantation rate is potentially reduced, with 43 fewer cases per 1000 people (RCT=32, n=7597, RR=0.80, 95% CI 0.66–0.95). Furthermore, the number of M II oocytes retrieved is fewer, with a reduction of 0.77 (RCT=12, n=2066, MD=-0.77, 95% CI -1.61–0.07), and the endometrial thickness may be decreased by 0.35 mm (RCT=11, n=1953, MD=-0.35, 95% CI -0.64– -0.06).  In people with POR, relative to agonists, the benefit of using GnRH antagonists is a potential increase in the cumulative live birth rate by 14 more per 1,000 patients (RCT=1, n=191, RR=1.08, 95% CI 0.60–1.95). However, the harms include a probable reduction in the clinical pregnancy rate by 26 per 1,000 patients (RCT=15, n=2039, RR=0.96, 95% CI 0.71–1.23) and a probable reduction in the implantation rate by 13 per 1,000 patients (RCT=6, n=1331, RR=0.93, 95% CI 0.71–1.22).  In people with HOR, the relative benefits include a probable reduction in the incidence of moderate to high-risk OHSS by 230 fewer cases per 1000 people (RCT=5, n=522, RR=0.48, 95% CI 0.27–0.86), a potentially increased clinical pregnancy rate (RCT=10, n=1041, RR=1.01, 95% CI 0.87–1.17), and number of M II oocytes retrieved (RCT=3, n=460, MD=0.72, 95% CI -0.91–2.36). However, the live birth rate is probably lowered by 7 fewer cases per 1000 people (RCT=1, n=208, RR=0.98, 95% CI 0.68–1.43), and implantation rate reduced by 19 fewer cases per 1000 people (RCT=2, n=395, RR=0.93, 95% CI 0.66–1.29), a probable decrease of endometrial thickness by 0.57 mm (RCT=5, n=723, MD=-0.57, 95% CI -1.19–0.05), and a probable increase in miscarriage rate (RCT=5, n=447, RR=1.02, 95% CI 0.64–1.63). | |
| Resources required  How large are the resource requirements (costs)? | | |
| Judgement | Research evidence | Additional considerations |
| ○ Large costs ● Moderate costs ○ Negligible costs and savings ○ Moderate savings ○ Large savings ○ Varies ○ Don't know | This guideline did not systematically search for resource requirements. Practical searches revealed that compared to agonists, the use of antagonists may increase moderate resource needs. Considering the overall cost of pituitary suppression, agonists are lower than antagonists, but in average cases per conception, agonists may be higher or lower than antagonists.  Evidence:  Retrieved evidence: a cross-sectional study from the USA found that when the out-of-pocket cost for GnRH-agonist and GnRH-antagonist were identical, preference for GnRH-antagonist was registered by 50.7% of patients (Sills 2013).  Another RCT in the USA found that the total pituitary suppression cost was significantly lower in group GnRHag as compared to GnRHant ($125.4 ± 14.0 vs 606.5 ± 102.6, p < 0.0001) (Maldonado 2011); however, the mean cost per pregnancy in the GnRHa was higher than in the GnRHant group ($19,671.80 ± 1,430.00 vs. $11,328.70 ± 907.20) (Maldonado 2013)  One study in Egypt found that the total cost per pregnancy was $6531 (40166 EP) in the hMG/antagonist protocol and $5008 (30800 EP) in the GnRH-a/hMG protocol which is statistically significant (Badrawi 2005). | |
| Certainty of evidence of required resources  What is the certainty of the evidence of resource requirements (costs)? | | |
| Judgement | Research evidence | Additional considerations |
| ○ Very low ○ Low ○ Moderate ○ High ● No included studies | Not applicable. | |
| Cost effectiveness  Does the cost-effectiveness of the intervention favor the intervention or the comparison? | | |
| Judgement | Research evidence | Additional considerations |
| ○ Favors the comparison ● Probably favors the comparison ○ Does not favor either the intervention or the comparison ○ Probably favors the intervention ○ Favors the intervention ○ Varies ○ No included studies | According to a Chinese study, the use of antagonists increases costs by 28,000 RMB per unit improvement in cumulative ongoing pregnancy rate compared to agonist protocols. Therefore, experts unanimously agree that the cost required to achieve a live birth is the key metric. However, relevant research is limited, and there is uncertainty regarding cost-effectiveness.  Evidence:  A master's thesis from Zhejiang University in 2020 found in fresh embryo cycles, the average cost per ongoing pregnancy was 130,160.66 RMB for short-acting GnRH-a long protocol versus 110,989.38 RMB for antagonist protocol. When including frozen embryo cycles to calculate cumulative ongoing pregnancy rates, the average cost per ongoing pregnancy obtained was 49,673.16 RMB for short-acting GnRH-a long protocol versus 53,476.01 RMB for antagonist protocol. The incremental cost-effectiveness ratio was 28,618.73 RMB (interpreted as an increase of 28,000 RMB per unit improvement in health). If only considering fresh embryo cycles, the antagonist protocol is less costly, showing economic advantages. However, when including total cumulative ongoing pregnancy rates, the short-acting GnRH-a long protocol demonstrates higher clinical and economic benefits (Jing, 2020).  Another Chinese study shows that the total costs per cycle in the long agonist protocol group are higher than in the antagonist group: 2,729.11 RMB per retrieved oocyte (agonist vs antagonist: 3,038.60 RMB), 8,867.19 RMB per good-quality embryo (8,867.19 vs. 9,644.85 RMB), and 77,598.06 RMB per clinical pregnancy (77,598.06 vs. 96,139.85 RMB). Therefore, the cost-effectiveness ratio (CER) is lower for the antagonist protocol (Chen, 2014). | |
| Equity  What would be the impact on health equity? | | |
| Judgement | Research evidence | Additional considerations |
| ○ Reduced ○ Probably reduced ● Probably no impact ○ Probably increased ○ Increased ○ Varies ○ Don't know | This guideline did not conduct a systematic search on resource requirements. Practical searches have shown that relative to agonists, the use of antagonists may increase moderate resource demands. Considering the overall cost of pituitary suppression, agonists are lower than antagonists, but in average cases per conception, agonists may be higher or lower than antagonists.  The absence of avoidable and unfair differences in a particular condition or state between different groups of people. For example, health equity is the absence of avoidable and unfair differences in health outcomes. | |
| Acceptability  Is the intervention acceptable to key stakeholders? | | |
| Judgement | Research evidence | Additional considerations |
| ○ No ○ Probably no ● Probably yes ○ Yes ○ Varies ○ Don't know | The experts discussed acceptability, primarily focusing on whether gonadotropins and antagonists are acceptable for use in China, considering factors such as pricing and administration methods. | |
| Feasibility  Is the intervention feasible to implement? | | |
| Judgement | Research evidence | Additional considerations |
| ○ No ○ Probably no ● Probably yes ○ Yes ○ Varies ○ Don't know | The experts discussed feasibility, primarily focusing on whether the use of gonadotropins and antagonists in China is reasonable and feasible, whether specific technical personnel are needed for medical interventions, and the availability of required technologies. | |

# PICO 2 Among patients undergoing IVF assisted reproduction with COS using GnRH-ant, is it recommended to implement pretreatments?

## **Table 2.** **Recommendations**

| Problem  Is the problem a priority? | | |
| --- | --- | --- |
| Judgement | Research evidence | Additional considerations |
| ○ No ○ Probably no ○ Probably yes ● Yes ○ Varies ○ Don't know | GnRH antagonists were introduced into the protocols for ovarian stimulation in assisted reproductive technology (ART) in the 1990s. These antagonists reversibly bind to GnRH receptors in the pituitary gland, rapidly suppressing the release of endogenous GnRH. This allows for a quick inhibition of pituitary function within a short time frame (1-2 hours), preventing premature LH surges while maintaining pituitary responsiveness. Due to these advantages, there has been a gradual increase in the clinical demand for GnRH antagonists in assisted reproductive therapies.  In response to these benefits, expert consensus guidelines for the clinical use of GnRH antagonists were established by the Chinese Medical Association's Reproductive Medicine Branch in 2015, and evidence-based guidelines on ovarian stimulation protocols were issued by the European Society of Reproductive Medicine in 2020. However, specific guidance on the use and management of GnRH antagonists in IVF treatment was lacking in existing guidelines. Consequently, the Reproductive Medicine Specialty Committee of the Chinese Medical Doctor Association initiated the development of clinical management guidelines for the use of GnRH antagonists in IVF treatment. These guidelines provide tailored recommendations for different ovarian response populations.  Poor ovarian reserve/response (POR): Currently, the internationally recognized criteria for POR are defined by the Bologna criteria. According to these criteria, a diagnosis of POR requires meeting at least two out of the following three conditions: ① Advanced maternal age (≥ 40 years) or other risk factors for poor ovarian response; ② Previous history of poor ovarian response (≤ 3 oocytes retrieved with conventional stimulation); ③ Abnormal results in ovarian reserve tests such as AFC < 5-7, AMH < 0.5-1.1 ng/ml, or FSH > 8.5 mIU/mL (Kim 2021).  If a patient does not meet the criteria for advanced age or has normal results in ovarian reserve tests, those who experience poor ovarian response in at least two previous maximum stimulation cycles can also be classified as poor responders. Additional criteria identified in literature searches before 2015 include: fewer than 4 mature follicles in the first IVF cycle, FSH levels of 10-15 mIU/ml, and estradiol levels below 1500/600 pg/ml. | |
| Desirable Effects  How substantial are the desirable anticipated effects? | | |
| Judgement | Research evidence | Additional considerations |
| ○ Trivial ○ Small ○ Moderate ○ Large ● Varies ○ Don't know | Table 1: Normal Ovarian Responses 1. Pretreatment with OCP Outcome 2 Outcomes \| № of participants (studies) Follow-up \| Certainty of the evidence (GRADE) \| Relative effect (95% CI) \| Anticipated absolute effects^*^ (95% CI) \|; \| \| \| \| Risk with Control \| Risk difference with OCP; Metaphase II oocytes- NOR \| 284 (2 RCTs) \| ⨁⨁⨁◯ Moderate^a,b^ \| - \| The mean metaphase II oocytes- NOR was 0 \| MD 1.56 higher (0.14 higher to 2.99 higher); Moderate risk of OHSS- NOR \| 629 (2 RCTs) \| ⨁◯◯◯ Very low^c,d^ \| RR 0.77 (0.13 to 4.70) \| Study population \|; \| \| \| \| 13 per 1,000 \| 3 fewer per 1,000 (11 fewer to 48 more) a. Included studies have the potential risk of selection bias (weight >20%). However, it did not affect the overall estimates. b. Downgraded by one level due to imprecision: low sample size. c. Downgraded by one level due to inconsistency: I^2^ is low but the point estimates are in different directions across included studies. d. Downgraded by two levels due to imprecision: low sample size and the effect could be both beneficial and harmful. 2. Pretreatment with Estrogen Outcome2 Outcomes \| № of participants (studies) Follow-up \| Certainty of the evidence (GRADE) \| Relative effect (95% CI) \| Anticipated absolute effects^*^ (95% CI) \|; \| \| \| \| Risk with Control \| Risk difference with Estrogen; Endometrial thickness (mm) - NOR \| 586 (2 RCTs) \| ⨁⨁⨁◯ Moderate^a^ \| - \| The mean endometrial thickness (mm) - NOR was 0 \| MD 0.01 higher (0.3 lower to 0.31 higher); Metaphase II oocytes-Start stimulation - NOR \| 588 (3 RCTs) \| ⨁⨁⨁◯ Moderate^a,b^ \| - \| The mean metaphase II oocytes-Start stimulation - NOR was 0 \| MD 0.53 higher (0.23 lower to 1.29 higher) a. Downgraded by one level due to imprecision: wide confidence interval. b. Although included studies have a high risk of performance bias, the outcome is unlikely to be affected. 3. Pretreatment with GnRH-ant Outcome 2 Outcomes \| № of participants (studies) Follow-up \| Certainty of the evidence (GRADE) \| Relative effect (95% CI) \| Anticipated absolute effects^*^ (95% CI) \|; \| \| \| \| Risk with Control \| Risk difference with GnRH-ant; Implantaion rate-NOR \| 289 (2 RCTs) \| ⨁◯◯◯ Very low^a,b,c^ \| RR 1.01 (0.75 to 1.36) \| Study population \|; \| \| \| \| 375 per 1,000 \| 4 more per 1,000 (94 fewer to 135 more); Moderate, severe risk of OHSS-Random - NOR \| 136 (1 RCT) \| ⨁⨁◯◯ Low^a,c^ \| RR 0.50 (0.05 to 5.39) \| Study population \|; \| \| \| \| 29 per 1,000 \| 15 fewer per 1,000 (28 fewer to 129 more) a. Although included studies have a high risk of performance bias, the outcome is unlikely to be affected. b. Downgraded by one level due to inconsistency: I^2^ is low and we consider it is because of the wide CI and small sample size. The point estimates are in different directions across included studies (weight>20%). c. Downgraded by two levels due to imprecision: low event rate and effect could be both beneficial or harmful. 4. Pretreatment with Testosterone Outcome 1 Outcomes \| № of participants (studies) Follow-up \| Certainty of the evidence (GRADE) \| Relative effect (95% CI) \| Anticipated absolute effects^*^ (95% CI) \|; \| \| \| \| Risk with Control \| Risk difference with Testosterone; Endometrial thickness on hCG day(mm)-Random - NOR \| 103 (1 RCT) \| ⨁⨁⨁◯ Moderate^a^ \| - \| The mean endometrial thickness on hCG day(mm)-Random - NOR was 0 \| MD 1 higher (0.14 higher to 1.86 higher) a. Downgraded by one level due to imprecision: low sample size. 5. Pretreatment with Progestogen Outcome 1 Outcomes \| № of participants (studies) Follow-up \| Certainty of the evidence (GRADE) \| Relative effect (95% CI) \| Anticipated absolute effects^*^ (95% CI) \|; \| \| \| \| Risk with Control \| Risk difference with Progestogen; Oocytes retrieved-NOR \| 47 (1 RCT) \| ⨁⨁◯◯ Low^a,b^ \| - \| The mean oocytes retrieved-NOR was 0 \| MD 2.7 higher (0.98 lower to 6.38 higher) a. a. Although included studies have a high risk of performance bias, the outcome is unlikely to be affected. b. Downgraded by two levels due to imprecision: low sample size and event rate, and the effect could be both beneficial and harmful. Table 2: Poor Ovarian Responses 1. Pretreatment with OCP Outcome 4 Outcomes \| № of participants (studies) Follow-up \| Certainty of the evidence (GRADE) \| Relative effect (95% CI) \| Anticipated absolute effects^*^ (95% CI) \|; \| \| \| \| Risk with Control \| Risk difference with OCP; Live birth rate - POR \| 134 (2 RCTs) \| ⨁⨁◯◯ Low^a,b^ \| RR 1.54 (0.84 to 2.83) \| Study population \|; \| \| \| \| 194 per 1,000 \| 105 more per 1,000 (31 fewer to 355 more); Implantation rate, % - POR \| 395 (2 RCTs) \| ⨁⨁◯◯ Low^a,b^ \| RR 1.57 (0.90 to 2.74) \| Study population \|; \| \| \| \| 92 per 1,000 \| 53 more per 1,000 (9 fewer to 161 more); Clinical pregnancy rate - POR \| 134 (2 RCTs) \| ⨁⨁◯◯ Low^a,b^ \| RR 1.53 (0.88 to 2.67) \| Study population \|; \| \| \| \| 224 per 1,000 \| 119 more per 1,000 (27 fewer to 374 more); Miscarriage rate - POR \| 38 (2 RCTs) \| ⨁⨁◯◯ Low^a,b^ \| RR 0.97 (0.18 to 5.26) \| Study population \|; \| \| \| \| 133 per 1,000 \| 4 fewer per 1,000 (109 fewer to 568 more) a. One study has a potential risk of selection bias. However, the effect is unlikely to be affected. b. Downgraded by two levels due to imprecision: low event rate and effect could be both beneficial or harmful. 2. Pretreatment with GnRH-ant Outcome 5 Outcomes \| № of participants (studies) Follow-up \| Certainty of the evidence (GRADE) \| Relative effect (95% CI) \| Anticipated absolute effects^*^ (95% CI) \|; \| \| \| \| Risk with Control \| Risk difference with GnRH-ant; Implantaion rate, %-POR \| 100 (2 RCTs) \| ⨁⨁◯◯ Low^a^ \| RR 2.06 (0.54 to 7.83) \| Study population \|; \| \| \| \| 59 per 1,000 \| 62 more per 1,000 (27 fewer to 402 more); Endometrial thickness at trigger (mm)-Random - POR \| 378 (4 RCTs) \| ⨁⨁⨁◯ Moderate^b^ \| - \| The mean endometrial thickness at trigger (mm)-Random - POR was 0 \| MD 0.82 higher (0.5 higher to 1.14 higher); Metaphase II oocytes-Random - POR \| 378 (4 RCTs) \| ⨁⨁◯◯ Low^c,d^ \| - \| The mean metaphase II oocytes-Random - POR was 0 \| MD 0.65 higher (0.46 lower to 1.75 higher); Clinical pregnancy rate - POR \| 294 (3 RCTs) \| ⨁⨁⨁◯ Moderate^b^ \| RR 2.68 (1.41 to 5.09) \| Study population \|; \| \| \| \| 80 per 1,000 \| 134 more per 1,000 (33 more to 326 more); Miscarriage rate - POR \| 171 (3 RCTs) \| ⨁⨁◯◯ Low^e^ \| RR 0.9 (0.3 to 2.7) \| Study population \|; \| \| \| \| 60 per 1,000 \| 6 fewer per 1,000 (42 fewer to 102 more) a. Downgraded by two levels due to imprecision: low sample size and the effect could be both beneficial and harmful. b. Downgraded by one level due to imprecision: low sample size. c. Downgraded by one level due to inconsistency: the I^2^ value is high (I^2^>80%). We explored the clinical, methdological and statistical characteristics of the studies, but the unexplained heterogeneity remained. This potentially reduces our certainty on the observed magnitude of effect. d. Downgraded by one level due to imprecision: the effect could be both beneficial and harmful. e. Downgraded by two levels due to imprecision: low sample size, event rate, and the effect could be both beneficial and harmful. 3. Pretreatment with Testosterone Outcome 5 Outcomes \| № of participants (studies) Follow-up \| Certainty of the evidence (GRADE) \| Relative effect (95% CI) \| Anticipated absolute effects^*^ (95% CI) \|; \| \| \| \| Risk with Control \| Risk difference with Testosterone; Live birth rate-Random - POR \| 110 (1 RCT) \| ⨁⨁◯◯ Low^a,b^ \| RR 2.14 (0.95 to 4.84) \| Study population \|; \| \| \| \| 127 per 1,000 \| 145 more per 1,000 (6 fewer to 489 more); Implantation rate-POR \| 278 (1 RCT) \| ⨁⨁⨁◯ Moderate^a,c^ \| RR 2.46 (1.12 to 5.41) \| Study population \|; \| \| \| \| 58 per 1,000 \| 85 more per 1,000 (7 more to 256 more); Clinical pregnancy rate - POR \| 327 (3 RCTs) \| ⨁⨁⨁◯ Moderate^d^ \| RR 2.26 (1.46 to 3.51) \| Study population \|; \| \| \| \| 141 per 1,000 \| 178 more per 1,000 (65 more to 354 more); Metaphase II oocytes-Random - POR \| 140 (1 RCT) \| ⨁⨁◯◯ Low^b^ \| - \| The mean metaphase II oocytes-Random - POR was 0 \| MD 0.8 higher (0.04 lower to 1.64 higher); Miscarriage rate-POR \| 25 (1 RCT) \| ⨁⨁◯◯ Low^a,b^ \| RR 0.94 (0.10 to 8.92) \| Study population \|; \| \| \| \| 125 per 1,000 \| 8 fewer per 1,000 (113 fewer to 990 more) a. Although included studies have a high risk of performance bias, the outcome is unlikely to be affected. b. Downgraded by two levels due to imprecision: low sample size and the effect could be both beneficial and harmful. c. Downgraded by one level due to imprecision: low event rate. d. Downgraded by one level due to high risk of attrition bias. Table 3: High Ovarian Responses 1. Pretreatment with GnRH-ant Outcomes 4 Outcomes \| № of participants (studies) Follow-up \| Certainty of the evidence (GRADE) \| Relative effect (95% CI) \| Anticipated absolute effects^*^ (95% CI) \|; \| \| \| \| Risk with Control \| Risk difference with GnRH-ant; Implantaion rate, %- HOR, favor GnRH-ant \| 88 (1 RCT) \| ⨁⨁◯◯ Low^a,b^ \| - \| The mean implantaion rate, %- HOR, favor GnRH-ant was 0 \| MD 8.7 higher (1.69 lower to 19.09 higher); Metaphase II oocytes-Random - HOR, favor GnRH-ant \| 88 (1 RCT) \| ⨁⨁◯◯ Low^a,b^ \| - \| The mean metaphase II oocytes-Random - HOR, favor GnRH-ant was 0 \| MD 0.55 higher (3.04 lower to 4.14 higher); Clinical pregnancy rate - HOR, favor GnRH-ant \| 88 (1 RCT) \| ⨁⨁◯◯ Low^a,b^ \| RR 4.61 (1.01 to 20.93) \| Study population \|; \| \| \| \| 40 per 1,000 \| 144 more per 1,000 (0 fewer to 797 more); Miscarriage rate - HOR, favor GnRH-ant \| 9 (1 RCT) \| ⨁⨁◯◯ Low^a,c^ \| RR 0.86 (0.17 to 4.37) \| Study population \|; \| \| \| \| 500 per 1,000 \| 70 fewer per 1,000 (415 fewer to 1,685 more) a. Included studies have an unclear risk of bias (weight >20%). However, it is unlikely to affect the overall estimates. b. Downgraded by two levels due to imprecision: low event rate and effect could be both beneficial or harmful. c. Downgraded by two levels due to imprecision: low sample size, low event rate and effect could be both beneficial and harmful. | |
| Undesirable Effects  How substantial are the undesirable anticipated effects? | | |
| Judgement | Research evidence | Additional considerations |
| ○ Large ○ Moderate ○ Small ○ Trivial ● Varies ○ Don't know | Table 1: Normal Ovarian Responses 1. Pretreatment with OCP Outcome 5 **Outcomes** \| **№ of participants (studies) Follow-up** \| **Certainty of the evidence (GRADE)** \| **Relative effect (95% CI)** \| **Anticipated absolute effects^*^ (95% CI)** \|; \| \| \| \| **Risk with Control** \| **Risk difference with OCP**; Live birth rate - NOR \| 44 (1 RCT) \| ⨁⨁◯◯ Low^a,b^ \| **RR 0.51** (0.15 to 1.73) \| Study population \|; \| \| \| \| 292 per 1,000 \| **143 fewer per 1,000** (248 fewer to 213 more); Cumulative live birth rate - NOR \| 52 (1 RCT) \| ⨁⨁◯◯ Low^b,c^ \| **RR 0.81** (0.43 to 1.53) \| Study population \|; \| \| \| \| 476 per 1,000 \| **90 fewer per 1,000** (271 fewer to 252 more); Endometrial thickness (mm) - NOR \| 548 (2 RCTs) \| ⨁⨁⨁◯ Moderate^a,c,d^ \| - \| The mean endometrial thickness (mm) - NOR was **0** \| MD **0.21 lower** (0.53 lower to 0.12 higher); Clinical pregnancy rate - NOR \| 690 (5 RCTs) \| ⨁⨁◯◯ Low^a,b^ \| **RR 0.77** (0.55 to 1.07) \| Study population \|; \| \| \| \| 425 per 1,000 \| **98 fewer per 1,000** (191 fewer to 30 more); Miscarriage rate - NOR \| 391 (4 RCTs) \| ⨁◯◯◯ Very low^d,e,f^ \| **RR 1.37** (0.84 to 2.25) \| Study population \|; \| \| \| \| 138 per 1,000 \| **51 more per 1,000** (22 fewer to 172 more) a. Although included studies have a high risk of performance bias, the outcome is unlikely to be affected. b. Downgraded by two levels due to imprecision: low sample size and the effect could be both beneficial and harmful. c. Included studies have the potential risk of selection bias (weight >20%). However, it did not affect the overall estimates. d. Downgraded by one level due to imprecision: the effect could be both beneficial and harmful. e. Included studies have the potential risk of selection bias (weight >20%). f. Downgraded by one level due to inconsistency: I^2^ is low but the point estimates are in different directions across included studies. 2. Pretreatment with Estrogen Outcome 5 **Outcomes** \| **№ of participants (studies) Follow-up** \| **Certainty of the evidence (GRADE)** \| **Relative effect (95% CI)** \| **Anticipated absolute effects^*^ (95% CI)** \|; \| \| \| \| **Risk with Control** \| **Risk difference with Estrogen**; Live birth rate - NOR \| 43 (1 RCT) \| ⨁⨁◯◯ Low^a,b^ \| **RR 0.54** (0.16 to 1.82) \| Study population \|; \| \| \| \| 292 per 1,000 \| **134 fewer per 1,000** (245 fewer to 239 more); Cumulative live birth rate \| 43 (1 RCT) \| ⨁⨁◯◯ Low^b^ \| **RR 0.57** (0.25 to 1.30) \| Study population \|; \| \| \| \| 476 per 1,000 \| **205 fewer per 1,000** (357 fewer to 143 more); Endometrial thickness (mm) - NOR \| 586 (2 RCTs) \| ⨁⨁⨁◯ Moderate^c^ \| - \| The mean endometrial thickness (mm) - NOR was **0** \| MD **0.01 higher** (0.3 lower to 0.31 higher); Clinical pregnancy rate - NOR \| 494 (7 RCTs) \| ⨁◯◯◯ Very low^a,b,d^ \| **RR 0.91** (0.68 to 1.23) \| Study population \|; \| \| \| \| 382 per 1,000 \| **34 fewer per 1,000** (122 fewer to 88 more); Miscarriage rate - NOR \| 506 (3 RCTs) \| ⨁◯◯◯ Very low^a,b,d^ \| **RR 1.02** (0.53 to 1.99) \| Study population \|; \| \| \| \| 68 per 1,000 \| **1 more per 1,000** (32 fewer to 68 more) a. Although included studies have a high risk of performance bias, the outcome is unlikely to be affected. b. Downgraded by two levels due to imprecision: low event rate and effect could be both beneficial or harmful. c. Downgraded by one level due to imprecision: wide confidence interval. d. Downgraded by one level due to inconsistency: I^2^ is low and we consider it is because of the wide CI and small sample size. The point estimates are in different directions across included studies. 3. Pretreatment with GnRH-ant Outcome 5 **Outcomes** \| **№ of participants (studies) Follow-up** \| **Certainty of the evidence (GRADE)** \| **Relative effect (95% CI)** \| **Anticipated absolute effects^*^ (95% CI)** \|; \| \| \| \| **Risk with Control** \| **Risk difference with GnRH-ant**; Live birth rate-NOR \| 117 (1 RCT) \| ⨁⨁◯◯ Low^a,b^ \| **RR 0.79** (0.49 to 1.25) \| Study population \|; \| \| \| \| 431 per 1,000 \| **91 fewer per 1,000** (220 fewer to 108 more); Clinical pregnancy rate - NOR \| 157 (2 RCTs) \| ⨁◯◯◯ Very low^a,b,c^ \| **RR 0.90** (0.64 to 1.26) \| Study population \|; \| \| \| \| 474 per 1,000 \| **47 fewer per 1,000** (171 fewer to 123 more); Metaphase II oocytes-NOR \| 620 (1 RCT) \| ⨁⨁⨁◯ Moderate^d^ \| **RR 1.00** (0.93 to 1.08) \| Study population \|; \| \| \| \| 805 per 1,000 \| **0 fewer per 1,000** (56 fewer to 64 more); Miscarriage rate - NOR \| 91 (2 RCTs) \| ⨁⨁◯◯ Low^a,b^ \| **RR 1.34** (0.44 to 4.03) \| Study population \|; \| \| \| \| 106 per 1,000 \| **36 more per 1,000** (60 fewer to 322 more); Ectopic pregnancy rate-NOR \| 58 (1 RCT) \| ⨁⨁◯◯ Low^a,b^ \| **RR 1.15** (0.17 to 7.61) \| Study population \|; \| \| \| \| 65 per 1,000 \| **10 more per 1,000** (54 fewer to 426 more) a. Although included studies have a high risk of performance bias, the outcome is unlikely to be affected. b. Downgraded by two levels due to imprecision: low event rate and effect could be both beneficial or harmful. c. Downgraded by one level due to inconsistency: I^2^ is low and we consider it is because of the wide CI and small sample size. The point estimates are in different directions across included studies (weight>20%). d. Downgraded by one level due to imprecision: effect could be both beneficial or harmful. 4. Pretreatment with Testosterone Outcome 2 **Outcomes** \| **№ of participants (studies) Follow-up** \| **Certainty of the evidence (GRADE)** \| **Relative effect (95% CI)** \| **Anticipated absolute effects^*^ (95% CI)** \|; \| \| \| \| **Risk with Control** \| **Risk difference with Testosterone**; Live birth rate-Random - NOR \| 103 (1 RCT) \| ⨁⨁◯◯ Low^a^ \| **RR 0.78** (0.43 to 1.41) \| Study population \|; \| \| \| \| 340 per 1,000 \| **75 fewer per 1,000** (194 fewer to 139 more); Implantation rate-NOR \| 135 (1 RCT) \| ⨁⨁⨁◯ Moderate^b^ \| **RR 0.79** (0.47 to 1.31) \| Study population \|; \| \| \| \| 344 per 1,000 \| **72 fewer per 1,000** (182 fewer to 107 more) a. Downgraded by two levels due to imprecision: low sample size and the effect could be both beneficial and harmful. b. Downgraded by one level due to imprecision: the effect could be both beneficial and harmful. 5. Pretreatment with Progestogen Outcome 2 **Outcomes** \| **№ of participants (studies) Follow-up** \| **Certainty of the evidence (GRADE)** \| **Relative effect (95% CI)** \| **Anticipated absolute effects^*^ (95% CI)** \|; \| \| \| \| **Risk with Control** \| **Risk difference with Progestogen**; Live birth rate-NOR \| 44 (1 RCT) \| ⨁⨁◯◯ Low^a,b^ \| **RR 0.86** (0.32 to 2.29) \| Study population \|; \| \| \| \| 292 per 1,000 \| **41 fewer per 1,000** (198 fewer to 376 more); Pregnancy rate-NOR \| 44 (1 RCT) \| ⨁⨁◯◯ Low^a,b^ \| **RR 0.70** (0.34 to 1.44) \| Study population \|; \| \| \| \| 500 per 1,000 \| **150 fewer per 1,000** (330 fewer to 220 more) a. a. Although included studies have a high risk of performance bias, the outcome is unlikely to be affected. b. Downgraded by two levels due to imprecision: low sample size and event rate, and the effect could be both beneficial and harmful. Table 2: Poor Ovarian Responses 1. Pretreatment with OCP **Outcomes** \| **№ of participants (studies) Follow-up** \| **Certainty of the evidence (GRADE)** \| **Relative effect (95% CI)** \| **Anticipated absolute effects^*^ (95% CI)** \|; \| \| \| \| **Risk with Control** \| **Risk difference with OCP**; Endometrial thickness (mm) - POR \| 134 (2 RCTs) \| ⨁⨁⨁◯ Moderate^a,b^ \| - \| The mean endometrial thickness (mm) - POR was **0** \| MD **0.27 lower** (0.88 lower to 0.35 higher) a. One study has a potential risk of selection bias. However, the effect is unlikely to be affected. b. Downgraded by one level due to imprecision: effect could be both beneficial or harmful. 2. Pretreatment with Testosterone **Outcomes** \| **№ of participants (studies) Follow-up** \| **Certainty of the evidence (GRADE)** \| **Relative effect (95% CI)** \| **Anticipated absolute effects^*^ (95% CI)** \|; \| \| \| \| **Risk with Control** \| **Risk difference with Testosterone**; Endometrial thickness on hCG day(mm)-Random - POR \| 110 (1 RCT) \| ⨁⨁◯◯ Low^a,b^ \| - \| The mean endometrial thickness on hCG day(mm)-Random - POR was **0** \| MD **0.1 lower** (0.59 lower to 0.39 higher) a. Although included studies have a high risk of performance bias, the outcome is unlikely to be affected. b. Downgraded by two levels due to imprecision: low sample size and the effect could be both beneficial and harmful. Table3: High Ovarian Responses 1. Pretreatment with GnRH-ant **Outcomes** \| **№ of participants (studies) Follow-up** \| **Certainty of the evidence (GRADE)** \| **Relative effect (95% CI)** \| **Anticipated absolute effects^*^ (95% CI)** \|; \| \| \| \| **Risk with Control** \| **Risk difference with GnRH-ant**; Moderate, severe risk of OHSS-Random - HOR, favor control \| 88 (1 RCT) \| ⨁⨁◯◯ Low^a,b^ \| **RR 1.10** (0.64 to 1.88) \| Study population \|; \| \| \| \| 360 per 1,000 \| **36 more per 1,000** (130 fewer to 317 more) a. Included studies have an unclear risk of bias (weight >20%). However, it is unlikely to affect the overall estimates. b. Downgraded by two levels due to imprecision: low sample size, low event rate and effect could be both beneficial and harmful. | |
| Certainty of evidence  What is the overall certainty of the evidence of effects? | | |
| Judgement | Research evidence | Additional considerations |
| ○ Very low ● Low ○ Moderate ○ High ○ No included studies | See the evidence table.  In the normal ovarian response population,   - The evidence quality for pretreatment with OCP is low, with an overall evidence quality of low. - The evidence quality for pretreatment with Estrogen is low, with an overall evidence quality of low. - The evidence quality for pretreatment with GnRH-ant is low to very low, with an overall evidence quality of very low. - The evidence quality for pretreatment with Testosterone is moderate to low, with an overall evidence quality of low. - The evidence quality for pretreatment with Progestogen is low, with an overall evidence quality of low.   In the low ovarian response population,   - The evidence quality for pretreatment with OCP is low, with an overall evidence quality of low. - The evidence quality for pretreatment with GnRH-ant is low, with an overall evidence quality of low. - The evidence quality for pretreatment with Testosterone is moderate to low, with an overall evidence quality of low.   In the high ovarian response population,   - Pretreatment with GnRH-ant in greater benefits and harms, with OHSS being uncommon with antagonist pre-treatment, small sample sizes, low quality, and not consistent with clinical practice. | |
| Values  Is there important uncertainty about or variability in how much people value the main outcomes? | | |
| Judgement | Research evidence | Additional considerations |
| ○ Important uncertainty or variability ○ Possibly important uncertainty or variability ● Probably no important uncertainty or variability ○ No important uncertainty or variability | No relevant patient values were identified.  The importance of outcome indicators in this guideline was determined through voting and discussions among a panel of clinical experts who considered the significance of clinical outcomes. Following discussions during the guideline meetings, clinical experts concluded that there may be no significant uncertainty or variability. | |
| Balance of effects  Does the balance between desirable and undesirable effects favor the intervention or the comparison? | | |
| Judgement | Research evidence | Additional considerations |
| ○ Favors the comparison ○ Probably favors the comparison ○ Does not favor either the intervention or the comparison ● Probably favors the intervention ○ Favors the intervention ○ Varies ○ Don't know | In the normal ovarian response population:  1. Pre-treatment with OCP:   - Desirable effect: Increase in MII oocytes (1.56, RCT=2, n=284, MD=1.56, 95% CI 0.14–2.99), simultaneous reduction in moderate OHSS (3 fewer per 1000, RCT=2, n=629, RR=0.77, 95% CI 0.13–4.70). - Undesirable effect: Decrease in live birth rate (143 fewer per 1000, RCT=1, n=44, RR=0.51, 95% CI 0.15–1.73), cumulative live birth rate (90 fewer per 1000, RCT=1, n=52, RR=0.81, 95% CI 0.43–1.53), endometrial thickness (0.21mm decrease, RCT=2, n=548, MD=-0.21, 95% CI -0.53–0.12), clinical pregnancy rate (98 fewer per 1000, RCT=5, n=690, RR=0.77, 95% CI 0.55–1.07), increase in miscarriage rate (51 more per 1000, RCT=4, n=391, RR=1.37, 95% CI 0.84–2.25). - Expert consensus: The benefit of using oral contraceptives for pretreatment is small, while harms are significant, particularly considering that harms outweigh benefits.   2. Pre-treatment with Estrogen:   - Desirable effect: Increase in MII oocytes (0.53, RCT=3, n=588, MD=0.53, 95% CI -0.23–1.29). - Undesirable effect: Decrease in live birth rate (134 fewer per 1000, RCT=1, n=43, RR=0.54, 95% CI 0.16–1.82), cumulative live birth rate (205 fewer per 1000, RCT=1, n=43, RR=0.57, 95% CI 0.25–1.30), implantation rate (29 fewer per 1000, RCT=1, n=660, RR=0.90, 95% CI 0.70–1.15), clinical pregnancy rate (34 fewer per 1000, RCT=7, n=494, RR=0.91, 95% CI 0.68–1.23), increase in miscarriage rate (1 more per 1000, RCT=3, n=506, RR=1.02, 95% CI 0.53–1.99), increase or decrease in endometrial thickness (0.01mm decrease, RCT=2, n=586, MD=-0.01, 95% CI -0.3–0.31). - Expert consensus: The benefit of using estrogen for pretreatment is small, while harms are significant, particularly with a substantial decrease in live birth rate, indicating that harms outweigh benefits.   3. Pre-treatment with GnRH-ant:   - Desirable effect: Increase in implantation rate (4 more per 1000, RCT=2, n=289, RR=1.01, 95% CI 0.75–1.36), decrease in moderate to severe OHSS (15 fewer per 1000, RCT=1, n=136, RR=0.50, 95% CI 0.05–5.39). - Undesirable effect: Decrease in live birth rate (91 fewer per 1000, RCT=1, n=117, RR=0.79, 95% CI 0.49–1.25), decrease in clinical pregnancy rate (47 fewer per 1000, RCT=2, n=157, RR=0.90, 95% CI 0.64–1.26), no increase in MII oocytes (RCT=1, n=620, RR=1.00, 95% CI 0.93–1.08), increase in miscarriage rate (36 more per 1000, RCT=2, n=91, RR=1.34, 95% CI 0.44–4.03), increase in ectopic pregnancy rate (10 per 1000, RCT=1, n=58, RR=1.15, 95% CI 0.17–7.61). - Expert consensus: The benefit of using antagonists for pretreatment is small, while harms are significant, with a notable 9% decrease in live birth rate, indicating that harms outweigh benefits. The benefit in reducing moderate OHSS is moderate, while other benefits are minor, suggesting that overall harms outweigh benefits.   4. Pre-treatment with Testosterone:   - Desirable effect: Increase in endometrial thickness (1mm, RCT=1, n=103, MD=1.00, 95% CI 0.14–1.86). - Undesirable effect: Decrease in live birth rate (75 fewer per 1000, RCT=1, n=103, RR=0.78, 95% CI 0.43–1.41), decrease in implantation rate (72 fewer per 1000, RCT=1, n=135, RR=0.79, 95% CI 0.47–1.31). - Expert consensus: The benefit of using androgens for pretreatment is small, while harms are significant. Therefore, this pretreatment is not recommended due to the imbalance between benefits and harms.   5. Pre-treatment with Progesterone:   - Desirable effect: Increase in number of oocytes retrieved (2.7, RCT=1, n=47, MD=2.70, 95% CI -0.98–6.38). - Undesirable effect: Decrease in live birth rate (41 fewer per 1000, RCT=1, n=44, RR=0.86, 95% CI 0.32–2.29), decrease in pregnancy rate (150 fewer per 1000, RCT=1, n=44, RR=0.70, 95% CI 0.34–1.44). - Expert consensus: The benefit of using progesterone for pretreatment is small, while harms are significant, suggesting that overall harms outweigh benefits.   In the poor ovarian response population:  1. Pre-treatment with OCP:   - Desriable effect: Increase in live birth rate (105 per 1000, RCT=2, n=134, RR=1.54, 95% CI 0.84–2.83), increase in implantation rate (53 per 1000, RCT=2, n=395, RR=1.57, 95% CI 0.90–2.74), increase in clinical pregnancy rate (119 per 1000, RCT=2, n=134, RR=1.53, 95% CI 0.88–2.67), decrease in miscarriage rate (4 per 1000, RCT=2, n=38, RR=0.97, 95% CI 0.18–5.26). - Undesriable effect: Decrease in endometrial thickness (0.27mm decrease, RCT=2, n=134, MD=-0.27, 95% CI -0.88–0.35). - Expert consensus: The benefit of using oral contraceptives for pretreatment is significant, while harms are minimal. Main consideration is given to the increase in live birth rate, which aligns with the results for implantation and pregnancy rates.   2. Pre-treatment with GnRH-ant:   - Desirable effect: Increase in embryo implantation rate (62 per 1000, RCT=2, n=100, RR=2.06, 95% CI 0.54–7.83), increase in endometrial thickness (0.82mm, RCT=4, n=378, MD=0.82, 95% CI 0.50–0.14), increase in MII oocytes (0.65, RCT=4, n=378, MD=0.65, 95% CI -0.46–1.75), increase in clinical pregnancy rate (134 per 1000, RCT=3, n=294, RR=2.68, 95% CI 1.41–5.09), decrease in miscarriage rate (6 per 1000, RCT=3, n=171, RR=0.90, 95% CI 0.30–2.70). - Expert consensus: The benefit of using antagonists for pretreatment is significant, while harms are minimal. Considering the substantial benefits outweigh minor harms.   3. Pre-treatment with Testosterone:   - Desirable effect: Increase in live birth rate (145 per 1000, RCT=1, n=110, RR=2.14, 95% CI 0.95–4.84), increase in implantation rate (85 per 1000, RCT=1, n=278, RR=2.46, 95% CI 1.12–5.41), increase in clinical pregnancy rate (178 per 1000, RCT=3, n=327, RR=2.26, 95% CI 1.46–3.51), increase in MII oocytes (0.8, RCT=1, n=140, MD=0.80, 95% CI -0.04–1.64), decrease in miscarriage rate (8 per 1000, RCT=1, n=25, RR=0.94, 95% CI 0.10–8.92). - Undesirable effect: Decrease in endometrial thickness (0.1mm decrease, RCT=1, n=110, MD=-0.10, 95% CI -0.59–0.39). - Expert consensus: The benefit of using androgens for pretreatment is significant, while harms are minimal. Considering the benefits outweigh harms.   In the high ovarian response population:  1. Pre-treatment with GnRH-ant:   - Desirable effect: Increase in implantation rate by 8.7% (RCT=1, n=88, MD=8.7, 95% CI -1.69–19.09), increase in number of M II oocytes by 0.55 (RCT=1, n=88, MD=0.55, 95% CI -3.04–4.14), increase in clinical pregnancy rate by 144 per 1000 (RCT=1, n=88, RR=4.61, 95% CI 1.01–20.93), decrease in miscarriage rate by 70 per 1000 (RCT=1, n=9, RR=0.86, 95% CI 0.17–4.37). - Undesirable effect: Increase in moderate/severe OHSS by 36 per 1000 (RCT=1, n=88, RR=1.10, 95% CI 0.64–1.88). - Expert consensus: In the high ovarian response population, pre-treatment with antagonists shows significant benefits but also significant harms. Considering the small sample size in the studies included and the low credibility of evidence, and because the use of antagonists for pre-treatment is not in line with clinical practice, experts unanimously agree not to recommend it for high response populations anymore. | |
| Resources required  How large are the resource requirements (costs)? | | |
| Judgement | Research evidence | Additional considerations |
| ○ Large costs ○ Moderate costs ● Negligible costs and savings ○ Moderate savings ○ Large savings ○ Varies ○ Don't know | This guideline did not systematically search for resource requirements. Practical searches did not find evidence related to the resource requirements of pre-treatments.  For patients with normal ovarian response, experts unanimously believe that the cost of using pre-treatments, including all medications, is relatively low in China. It accounts for a fraction of a percent of the entire IVF process and can be considered negligible. | |
| Certainty of evidence of required resources  What is the certainty of the evidence of resource requirements (costs)? | | |
| Judgement | Research evidence | Additional considerations |
| ○ Very low ○ Low ○ Moderate ○ High ● No included studies | Not applicable. | |
| Cost effectiveness  Does the cost-effectiveness of the intervention favor the intervention or the comparison? | | |
| Judgement | Research evidence | Additional considerations |
| ○ Favors the comparison ○ Probably favors the comparison ○ Does not favor either the intervention or the comparison ○ Probably favors the intervention ○ Favors the intervention ○ Varies ● No included studies | No relevant evidence. | |
| Equity  What would be the impact on health equity? | | |
| Judgement | Research evidence | Additional considerations |
| ○ Reduced ○ Probably reduced ● Probably no impact ○ Probably increased ○ Increased ○ Varies ○ Don't know | The experts discussed equity, primarily focusing on whether the use or non-use of pre-treatments in different regions of China would create disparities, i.e., whether different populations in different regions can access the recommended interventions in this guideline under circumstances beyond their control. After discussion, the experts unanimously agreed that the use of pre-treatments is unlikely to affect equity. | |
| Acceptability  Is the intervention acceptable to key stakeholders? | | |
| Judgement | Research evidence | Additional considerations |
| ○ No ○ Probably no ● Probably yes ○ Yes ○ Varies ○ Don't know | The experts discussed the acceptability of pre-treatment, primarily focusing on whether its use versus non-use among the Chinese population is acceptable, including factors such as willingness to pay the price, mode of administration, and other considerations. | |
| Feasibility  Is the intervention feasible to implement? | | |
| Judgement | Research evidence | Additional considerations |
| ○ No ○ Probably no ● Probably yes ○ Yes ○ Varies ○ Don't know | The experts discussed feasibility, primarily focusing on whether the use of pre-treatment in China is reasonable and feasible, whether medical interventions require specific technical personnel for implementation, and the availability of necessary technologies. | |

# PICO 3 Should fixed regimen or flexible regimen be preferred under GnRH-ant protocol?

## **Table 3.** **Recommendations**

| Problem  Is the problem a priority? | | |
| --- | --- | --- |
| Judgement | Research evidence | Additional considerations |
| ○ No ○ Probably no ○ Probably yes ● Yes ○ Varies ○ Don't know | GnRH antagonists were introduced into the protocols for ovarian stimulation in assisted reproductive technology (ART) in the 1990s. These antagonists reversibly bind to GnRH receptors in the pituitary gland, rapidly suppressing the release of endogenous GnRH. This allows for a quick inhibition of pituitary function within a short time frame (1-2 hours), preventing premature LH surges while maintaining pituitary responsiveness. Due to these advantages, there has been a gradual increase in the clinical demand for GnRH antagonists in assisted reproductive therapies.  In response to these benefits, expert consensus guidelines for the clinical use of GnRH antagonists were established by the Chinese Medical Association's Reproductive Medicine Branch in 2015, and evidence-based guidelines on ovarian stimulation protocols were issued by the European Society of Reproductive Medicine in 2020. However, specific guidance on the use and management of GnRH antagonists in IVF treatment was lacking in existing guidelines. Consequently, the Reproductive Medicine Specialty Committee of the Chinese Medical Doctor Association initiated the development of clinical management guidelines for the use of GnRH antagonists in IVF treatment. These guidelines provide tailored recommendations for different ovarian response populations.  Poor ovarian reserve/response (POR): Currently, the internationally recognized criteria for POR are defined by the Bologna criteria. According to these criteria, a diagnosis of POR requires meeting at least two out of the following three conditions: ① Advanced maternal age (≥ 40 years) or other risk factors for poor ovarian response; ② Previous history of poor ovarian response (≤ 3 oocytes retrieved with conventional stimulation); ③ Abnormal results in ovarian reserve tests such as AFC < 5-7, AMH < 0.5-1.1 ng/ml, or FSH > 8.5 mIU/mL (Kim 2021).  If a patient does not meet the criteria for advanced age or has normal results in ovarian reserve tests, those who experience poor ovarian response in at least two previous maximum stimulation cycles can also be classified as poor responders. Additional criteria identified in literature searches before 2015 include: fewer than 4 mature follicles in the first IVF cycle, FSH levels of 10-15 mIU/ml, and estradiol levels below 1500/600 pg/ml. | |
| Desirable Effects  How substantial are the desirable anticipated effects? | | |
| Judgement | Research evidence | Additional considerations |
| ○ Trivial ○ Small ● Moderate ○ Large ○ Varies ○ Don't know | Table 1: Normal Ovarian Responses Outcome 4 **Outcomes** \| **№ of participants (studies) Follow-up** \| **Certainty of the evidence (GRADE)** \| **Relative effect (95% CI)** \| **Anticipated absolute effects^*^ (95% CI)** \|; \| \| \| \| **Risk with Flexible** \| **Risk difference with Fixed**; Cumulation Live birth rate-NOR, favor fixed \| 818 (2 RCTs) \| ⨁⨁◯◯ Low^a,b^ \| **RR 1.04** (0.89 to 1.21) \| Study population \|; \| \| \| \| 369 per 1,000 \| **15 more per 1,000** (41 fewer to 78 more); Implantation rate-NOR, favor fixed \| 185 (1 RCT) \| ⨁⨁◯◯ Low^c^ \| **RR 1.03** (0.68 to 1.57) \| Study population \|; \| \| \| \| 319 per 1,000 \| **10 more per 1,000** (102 fewer to 182 more); Clinical pregnancy rate-NOR, favor fixed \| 476 (4 RCTs) \| ⨁⨁⨁◯ Moderate^b,d^ \| **RR 1.16** (0.89 to 1.51) \| Study population \|; \| \| \| \| 270 per 1,000 \| **43 more per 1,000** (30 fewer to 138 more); Early pregnancy loss-NOR, favor fixed \| 286 (2 RCTs) \| ⨁◯◯◯ Very low^a,c^ \| **RR 0.50** (0.09 to 2.65) \| Study population \|; \| \| \| \| 28 per 1,000 \| **14 fewer per 1,000** (25 fewer to 46 more) a. Included studies have the potential risk of selection bias (weight >20%) b. Downgraded by one level due to imprecision: effect could be both benefit and harmful. c. Downgraded by two levels due to imprecision: low event rate and effect could be both beneficial and harmful. d. Although included studies have a high risk of performance bias, the outcome is unlikely to be affected. | |
| Undesirable Effects  How substantial are the undesirable anticipated effects? | | |
| Judgement | Research evidence | Additional considerations |
| ○ Trivial ○ Small ● Moderate ○ Large ○ Varies ○ Don't know | Table 1: Normal Ovarian Responses Outcome 3 **Outcomes** \| **№ of participants (studies) Follow-up** \| **Certainty of the evidence (GRADE)** \| **Relative effect (95% CI)** \| **Anticipated absolute effects^*^ (95% CI)** \|; \| \| \| \| **Risk with Flexible** \| **Risk difference with Fixed**; Moderate OHSS-NOR, favor flexible \| 201 (1 RCT) \| ⨁⨁◯◯ Low^a,b^ \| **RR 1.26** (0.35 to 4.57) \| Study population \|; \| \| \| \| 40 per 1,000 \| **10 more per 1,000** (26 fewer to 141 more); Metaphase II oocytes-NOR, favor flexible \| 454 (3 RCTs) \| ⨁⨁⨁◯ Moderate^a,c^ \| - \| The mean metaphase II oocytes-NOR, favor flexible was **0** \| MD **0.08 lower** (0.77 lower to 0.6 higher); Endometrial thickness on the day of ET, mm-NOR, favor flexible \| 93 (1 RCT) \| ⨁⨁◯◯ Low^a,d^ \| - \| The mean endometrial thickness on the day of ET, mm-NOR, favor flexible was **0** \| MD **0.3 lower** (0.85 lower to 0.25 higher) a. Although included studies have a high risk of performance bias, the outcome is unlikely to be affected. b. Downgraded by two levels due to imprecision: low event rate and effect could be both beneficial and harmful. c. Downgraded by one level due to imprecision: effect could be both benefit and harmful. d. Downgraded by two levels due to imprecision: small sample size and effect could be both beneficial and harmful. | |
| Certainty of evidence  What is the overall certainty of the evidence of effects? | | |
| Judgement | Research evidence | Additional considerations |
| ○ Very low ● Low ○ Moderate ○ High ○ No included studies | The certainty of evidence among the studies conducted in NOR population is low. | |
| Values  Is there important uncertainty about or variability in how much people value the main outcomes? | | |
| Judgement | Research evidence | Additional considerations |
| ○ Important uncertainty or variability ○ Possibly important uncertainty or variability ● Probably no important uncertainty or variability ○ No important uncertainty or variability | No relevant patient values were identified.  The importance of outcome indicators in this guideline was determined through voting and discussions among a panel of clinical experts who considered the significance of clinical outcomes. Following discussions during the guideline meetings, clinical experts concluded that there may be no significant uncertainty or variability. | |
| Balance of effects  Does the balance between desirable and undesirable effects favor the intervention or the comparison? | | |
| Judgement | Research evidence | Additional considerations |
| ○ Favors the comparison ○ Probably favors the comparison ○ Does not favor either the intervention or the comparison ● Probably favors the intervention ○ Favors the intervention ○ Varies ○ Don't know | In people with NOR, the fixed protocol offers several benefits over the flexible protocol. It may increase the cumulative live birth rate by 15 per 1000 people (RCT=2, n=818, RR=1.04, 95% CI 0.89–1.21), implantation rate by 10 per 1000 people (RCT=1, n=185, RR=1.03, 95% CI 0.68–1.57), clinical pregnancy rate by 43 per 1000 people (RCT=4, n=476, RR=1.16, 95% CI 0.89–1.51), and it may reduce early miscarriage by 14 per 1000 people (RCT=2, n=286, RR=0.50, 95% CI 0.09–2.65). However, it also may increases moderate OHSS by 10 per 1000 people (RCT=1, n=201, RR=1.26, 95% CI 0.35–4.57), reduces MII oocytes by 0.08 (RCT=3, n=454, MD=-0.08, 95% CI -0.77–0.60), and decreases endometrial thickness by 0.3 mm (RCT=1, n=93, MD=-0.30, 95% CI -0.85–0.25). The GDG concluded that the benefit of the fixed protocol probably outweighs the harms compared to the flexible protocol. | |
| Resources required  How large are the resource requirements (costs)?" | | |
| Judgement | Research evidence | Additional considerations |
| ○ Large costs ○ Moderate costs ○ Negligible costs and savings ○ Moderate savings ○ Large savings ○ Varies ● Don't know | This guideline did not systematically search for resource requirements. | |
| Certainty of evidence of required resources  What is the certainty of the evidence of resource requirements (costs)? | | |
| Judgement | Research evidence | Additional considerations |
| ○ Very low ○ Low ○ Moderate ○ High ● No included studies | Not applicable. | |
| Cost effectiveness  Does the cost-effectiveness of the intervention favor the intervention or the comparison? | | |
| Judgement | Research evidence | Additional considerations |
| ○ Favors the comparison ○ Probably favors the comparison ○ Does not favor either the intervention or the comparison ○ Probably favors the intervention ○ Favors the intervention ○ Varies ● No included studies | No relevant evidence. | |
| Equity  What would be the impact on health equity? | | |
| Judgement | Research evidence | Additional considerations |
| ○ Reduced ○ Probably reduced ● Probably no impact ○ Probably increased ○ Increased ○ Varies ○ Don't know | The experts need to discuss fairness, mainly focusing on whether the application of fixed protocols vs. flexible protocols in different regions of China would cause unfairness. Specifically, whether different populations in different regions can access the interventions recommended by this guideline equally under circumstances beyond their control during normal treatment.  The use of fixed protocols may not affect equity. | |
| Acceptability  Is the intervention acceptable to key stakeholders? | | |
| Judgement | Research evidence | Additional considerations |
| ○ No ○ Probably no ● Probably yes ○ Yes ○ Varies ○ Don't know | The experts discussed acceptability and found no factors that would impact the recommendations. | |
| Feasibility  Is the intervention feasible to implement? | | |
| Judgement | Research evidence | Additional considerations |
| ○ No ○ Probably no ● Probably yes ○ Yes ○ Varies ○ Don't know | The experts discussed feasibility and found no factors that would impact the recommendations. | |

# PICO 4 Is early initiation of GnRH-ant during stimulation recommended?

## **Table 4.** **Recommedations**

| Problem  Is the problem a priority? | | |
| --- | --- | --- |
| Judgement | Research evidence | Additional considerations |
| ○ No ○ Probably no ○ Probably yes ● Yes ○ Varies ○ Don't know | GnRH antagonists were introduced into the protocols for ovarian stimulation in assisted reproductive technology (ART) in the 1990s. These antagonists reversibly bind to GnRH receptors in the pituitary gland, rapidly suppressing the release of endogenous GnRH. This allows for a quick inhibition of pituitary function within a short time frame (1-2 hours), preventing premature LH surges while maintaining pituitary responsiveness. Due to these advantages, there has been a gradual increase in the clinical demand for GnRH antagonists in assisted reproductive therapies.  In response to these benefits, expert consensus guidelines for the clinical use of GnRH antagonists were established by the Chinese Medical Association's Reproductive Medicine Branch in 2015, and evidence-based guidelines on ovarian stimulation protocols were issued by the European Society of Reproductive Medicine in 2020. However, specific guidance on the use and management of GnRH antagonists in IVF treatment was lacking in existing guidelines. Consequently, the Reproductive Medicine Specialty Committee of the Chinese Medical Doctor Association initiated the development of clinical management guidelines for the use of GnRH antagonists in IVF treatment. These guidelines provide tailored recommendations for different ovarian response populations.  Poor ovarian reserve/response (POR): Currently, the internationally recognized criteria for POR are defined by the Bologna criteria. According to these criteria, a diagnosis of POR requires meeting at least two out of the following three conditions: ① Advanced maternal age (≥ 40 years) or other risk factors for poor ovarian response; ② Previous history of poor ovarian response (≤ 3 oocytes retrieved with conventional stimulation); ③ Abnormal results in ovarian reserve tests such as AFC < 5-7, AMH < 0.5-1.1 ng/ml, or FSH > 8.5 mIU/mL (Kim 2021).  If a patient does not meet the criteria for advanced age or has normal results in ovarian reserve tests, those who experience poor ovarian response in at least two previous maximum stimulation cycles can also be classified as poor responders. Additional criteria identified in literature searches before 2015 include: fewer than 4 mature follicles in the first IVF cycle, FSH levels of 10-15 mIU/ml, and estradiol levels below 1500/600 pg/ml. | |
| Desirable Effects  How substantial are the desirable anticipated effects? | | |
| Judgement | Research evidence | Additional considerations |
| ○ Trivial ○ Small ● Moderate ○ Large ○ Varies ○ Don't know | Table 1: Normal Ovarian Responses Outcomes 2 **Outcomes** \| **№ of participants (studies) Follow-up** \| **Certainty of the evidence (GRADE)** \| **Relative effect (95% CI)** \| **Anticipated absolute effects^*^ (95% CI)** \|; \| \| \| \| **Risk with Late administration** \| **Risk difference with Early administration**; Clinical pregnancy rate-NOR, favor late \| 60 (1 RCT) \| ⨁◯◯◯ Very low^a,b^ \| **RR 1.33** (0.66 to 269.00) \| Study population \|; \| \| \| \| 300 per 1,000 \| **99 more per 1,000** (102 fewer to 80,400 more); Implantation rate,% NOR, favor early \| 120 (2 RCTs) \| ⨁⨁◯◯ Low^a,c^ \| - \| The mean implantation rate,% NOR, favor early was **0** \| MD **3.4 higher** (3.23 higher to 3.57 higher) a. Included studies have the potential risk of selection bias (weight >20%). b. Downgraded by two levels due to imprecision: low sample size and the effect could be both beneficial and harmful. c. Downgraded by one level due to low sample size. Table 2: Poor Ovarian Responses Outcomes 2 **Outcomes** \| **№ of participants (studies) Follow-up** \| **Certainty of the evidence (GRADE)** \| **Relative effect (95% CI)** \| **Anticipated absolute effects^*^ (95% CI)** \|; \| \| \| \| **Risk with Flexible administration** \| **Risk difference with Early administration**; Implantation rate - POR-favour I \| 116 (1 RCT) \| ⨁⨁⨁◯ Moderate^a,b^ \| - \| The mean implantation rate - POR-favour I was **0** \| MD **0.25 higher** (0.13 higher to 0.37 higher); Oocytes retrieved - POR-favour I \| 116 (1 RCT) \| ⨁⨁◯◯ Low^a,c^ \| - \| The mean oocytes retrieved - POR-favour I was **0** \| MD **0.97 higher** (0.01 lower to 1.95 higher) a. Downgraded by 0.5 level due to ROB: there is a high risk of bias. b. Downgraded by 1 level due to imprecision: small sample size. c. Downgraded by 2 levels due to imprecision: wide confidence interval and small sample size. Table3: High Ovarian Responses Outcomes 5 **Outcomes** \| **№ of participants (studies) Follow-up** \| **Certainty of the evidence (GRADE)** \| **Relative effect (95% CI)** \| **Anticipated absolute effects^*^ (95% CI)** \|; \| \| \| \| **Risk with Late administration** \| **Risk difference with Early administration**; Live births rate-NOR, favor late \| 24 (1 RCT) \| ⨁◯◯◯ Very low^a,b^ \| **RR 1.25** (0.44 to 3.55) \| Study population \|; \| \| \| \| 333 per 1,000 \| **83 more per 1,000** (187 fewer to 850 more); Clinical pregnancy rate-POR+NOR+HOR, favor late \| 146 (2 RCTs) \| ⨁⨁◯◯ Low^c,d^ \| **RR 1.08** (0.31 to 3.72) \| Study population \|; \| \| \| \| 541 per 1,000 \| **43 more per 1,000** (373 fewer to 1,470 more); Endometrial thickness(mm)-POR+HOR, favor late \| 122 (1 RCT) \| ⨁⨁◯◯ Low^d^ \| - \| The mean endometrial thickness(mm)-POR+HOR, favor late was **0** \| MD **0.2 higher** (0.49 lower to 0.89 higher); Oocytes retrieved \| 122 (1 RCT) \| ⨁⨁◯◯ Low^d^ \| - \| The mean oocytes retrieved was **0** \| MD **2.2 higher** (0.21 lower to 4.61 higher); Implantation rate,%-HOR favor early \| 24 (1 RCT) \| ⨁⨁◯◯ Low^a,e^ \| - \| The mean implantation rate,%-HOR favor early was **0** \| MD **15.3 higher** (15.03 higher to 15.57 higher) a. Included studies have the potential risk of selection bias (weight >20%). b. Downgraded by two levels due to imprecision: low sample size, low event rate and effect could be both beneficial and harmful. c. Included studies have the potential risk of selection bias (weight >20%). However, it did not affect the overall estimates. d. Downgraded by two levels due to imprecision: low sample size and the effect could be both beneficial and harmful. e. Downgraded by one level due to imprecision: very low sample size. | |
| Undesirable Effects  How substantial are the undesirable anticipated effects? | | |
| Judgement | Research evidence | Additional considerations |
| ○ Large ● Moderate ○ Small ○ Trivial ○ Varies ○ Don't know | Table 1: Normal Ovarian Responses Outcomes 1 **Outcomes** \| **№ of participants (studies) Follow-up** \| **Certainty of the evidence (GRADE)** \| **Relative effect (95% CI)** \| **Anticipated absolute effects^*^ (95% CI)** \|; \| \| \| \| **Risk with Late administration** \| **Risk difference with Early administration**; Live births rate-NOR, favor late \| 617 (1 RCT) \| ⨁⨁◯◯ Low^a,b^ \| **RR 0.89** (0.67 to 1.20) \| Study population \|; \| \| \| \| 239 per 1,000 \| **26 fewer per 1,000** (79 fewer to 48 more) a. Included studies have the potential risk of selection bias (weight >20%). b. Downgraded by one level due to imprecision: the effect could be both beneficial and harmful. Table 2: Poor Ovarian Responses Outcomes 2 **Outcomes** \| **№ of participants (studies) Follow-up** \| **Certainty of the evidence (GRADE)** \| **Relative effect (95% CI)** \| **Anticipated absolute effects^*^ (95% CI)** \|; \| \| \| \| **Risk with Flexible administration** \| **Risk difference with Early administration**; Metaphase II oocytes-favour I \| 116 (1 RCT) \| ⨁⨁⨁◯ Moderate^a,b^ \| - \| The mean metaphase II oocytes-favour I was **0** \| MD **0.89 higher** (0.02 higher to 1.76 higher); Top quality embryos-favour I \| 116 (1 RCT) \| ⨁⨁◯◯ Low^a,c^ \| - \| The mean top quality embryos-favour I was **0** \| MD **0.31 higher** (0.24 lower to 0.86 higher) a. Downgraded by 0.5 level due to ROB: there is a high risk of bias. b. Downgraded by 1 level due to imprecision: small sample size. c. Downgraded by 2 levels due to imprecision: wide confidence interval and small sample size. | |
| Certainty of evidence  What is the overall certainty of the evidence of effects? | | |
| Judgement | Research evidence | Additional considerations |
| ● Very low ○ Low ○ Moderate ○ High ○ No included studies | In the normal ovarian response population, the quality of evidence for early initiation versus late initiation of treatment on key outcome indicators is low to very low, with an overall evidence quality assessed as very low according to GRADE guidelines.  In the low ovarian response population, the quality of evidence for early initiation versus late initiation of treatment on key outcome indicators is moderate to,low with an overall evidence quality assessed as low according to GRADE guidelines.  In the high ovarian response population, the quality of evidence for early initiation versus late initiation of treatment on key outcome indicators is low to very low, with an overall evidence quality assessed as very low according to GRADE guidelines. | |
| Values  Is there important uncertainty about or variability in how much people value the main outcomes? | | |
| Judgement | Research evidence | Additional considerations |
| ○ Important uncertainty or variability ○ Possibly important uncertainty or variability ● Probably no important uncertainty or variability ○ No important uncertainty or variability | No relevant patient values were identified.  The importance of outcome indicators in this guideline was determined through voting and discussions among a panel of clinical experts who considered the significance of clinical outcomes. Following discussions during the guideline meetings, clinical experts concluded that there may be no significant uncertainty or variability. | |
| Balance of effects  Does the balance between desirable and undesirable effects favor the intervention or the comparison? | | |
| Judgement | Research evidence | Additional considerations |
| ○ Favors the comparison ○ Probably favors the comparison ○ Does not favor either the intervention or the comparison ● Probably favors the intervention ○ Favors the intervention ○ Varies ○ Don't know | In people with NOR，early initiation shows probable benefits such as a 99 per 1000 increase in clinical pregnancy rate (RCT=1, n=60, RR=1.33, 95% CI 0.66–2.69) and a 1.11% increase in implantation rate or 33 per 1000 (RCT=1, n=60, MD=1.11, 95% CI -11.51–13.73; RR=1.11, 95% CI 0.53–2.34). However, it may also reduce the live birth rate by 26 per 1000 (RCT=1, n=617, RR=0.89, 95% CI 0.67–1.20). The GDG concluded that, due to limited evidence, the benefits and harms of early initiation compared to late initiation remain uncertain.  In people with POR, early initiation may increase the embryo implantation rate (by 0.25%, RCT=1, n=116, RR=0.25, 95% CI 0.13–0.37) and the number of oocytes retrieved (by 0.97, RCT=1, n=116, MD=0.97, 95% CI -0.01–1.95). However, it may also reduce the number of MII oocytes (by 0.89, RCT=1, n=116, MD=-0.89, 95% CI 0.02–1.76) and high-quality embryos (by 0.31, RCT=1, n=116, MD=-0.31, 95% CI -0.24–0.86). The GDG is uncertain if early initiation offers more benefits than harms compared to late initiation, as the evidence is of very low certainty.  In people with HOR, early initiation may increase live birth rate by 83 per 1000 (RCT=1, n=24, RR=1.25, 95% CI 0.44–3.55), clinical pregnancy rate by 114 per 1000 (RCT=2, n=146, RR=1.21, 95% CI 0.93–1.58), endometrial thickness by 0.2 mm (RCT=1, n=122, MD=0.20, 95% CI -0.49–0.89), oocytes retrieved by 2.2 (RCT=1, n=122, MD=2.20, 95% CI -0.21–4.61), and implantation rate by 167 per 1000 (RCT=1, n=24, RR=2.00, 95% CI 0.45–8.94). There is a lack of evidence of harm. The GDG has limited confidence in the observed effect due to the small sample size and missing outcomes such as OHSS, hence no recommendation is made. | |
| Resources required  How large are the resource requirements (costs)? | | |
| Judgement | Research evidence | Additional considerations |
| ○ Large costs ○ Moderate costs ○ Negligible costs and savings ○ Moderate savings ○ Large savings ● Varies ○ Don't know | This guideline did not conduct a systematic search on resource requirements. Practical searches did not find evidence regarding additional resources needed for relevant fixed protocols. Considering that early administration of GnRH antagonists may increase stimulation time (2-3 days), involving increased usage of GnRH -ant and potentially increasing the resources required per MII oocyte, particularly in high responder populations, final recommendations should fully consider these additional resource implications when making recommendations. | |
| Certainty of evidence of required resources  What is the certainty of the evidence of resource requirements (costs)? | | |
| Judgement | Research evidence | Additional considerations |
| ○ Very low ○ Low ○ Moderate ○ High ● No included studies | Not applicable. | |
| Cost effectiveness  Does the cost-effectiveness of the intervention favor the intervention or the comparison? | | |
| Judgement | Research evidence | Additional considerations |
| ○ Favors the comparison ○ Probably favors the comparison ○ Does not favor either the intervention or the comparison ○ Probably favors the intervention ○ Favors the intervention ○ Varies ● No included studies | No relevant evidence. | |
| Equity  What would be the impact on health equity? | | |
| Judgement | Research evidence | Additional considerations |
| ○ Reduced ○ Probably reduced ● Probably no impact ○ Probably increased ○ Increased ○ Varies ○ Don't know | The experts discussed equity, primarily focusing on whether the use of early initiation protocols versus conventional protocols in different regions of China could lead to inequality. Specifically, they considered whether different regional populations can access the recommended intervention measures in this guideline under circumstances beyond their control during regular treatment. | |
| Acceptability  Is the intervention acceptable to key stakeholders? | | |
| Judgement | Research evidence | Additional considerations |
| ○ No ○ Probably no ● Probably yes ○ Yes ○ Varies ○ Don't know | The experts discussed the acceptability of early initiation, primarily focusing on whether early initiation versus late initiation is acceptable among the Chinese population, including factors such as willingness to pay, method of administration, etc. | |
| Feasibility  Is the intervention feasible to implement? | | |
| Judgement | Research evidence | Additional considerations |
| ○ No ○ Probably no ● Probably yes ○ Yes ○ Varies ○ Don't know | The experts discussed the feasibility, primarily focusing on whether early initiation versus late initiation is reasonable and feasible in use in China, whether medical intervention requires specific technical personnel for implementation, and whether the necessary technology exists. | |

# PICO 5 Is the GnRHa add-on trigger recommended over the HCG trigger alone?

## **Table 5.** **Recommedations**

| Problem  Is the problem a priority? | | |
| --- | --- | --- |
| Judgement | Research evidence | Additional considerations |
| ○ No ○ Probably no ○Probably yes ●Yes ○ Varies ○ Don't know | GnRH antagonists were introduced into the protocols for ovarian stimulation in assisted reproductive technology (ART) in the 1990s. These antagonists reversibly bind to GnRH receptors in the pituitary gland, rapidly suppressing the release of endogenous GnRH. This allows for a quick inhibition of pituitary function within a short time frame (1-2 hours), preventing premature LH surges while maintaining pituitary responsiveness. Due to these advantages, there has been a gradual increase in the clinical demand for GnRH antagonists in assisted reproductive therapies.  In response to these benefits, expert consensus guidelines for the clinical use of GnRH antagonists were established by the Chinese Medical Association's Reproductive Medicine Branch in 2015, and evidence-based guidelines on ovarian stimulation protocols were issued by the European Society of Reproductive Medicine in 2020. However, specific guidance on the use and management of GnRH antagonists in IVF treatment was lacking in existing guidelines. Consequently, the Reproductive Medicine Specialty Committee of the Chinese Medical Doctor Association initiated the development of clinical management guidelines for the use of GnRH antagonists in IVF treatment. These guidelines provide tailored recommendations for different ovarian response populations.  Poor ovarian reserve/response (POR): Currently, the internationally recognized criteria for POR are defined by the Bologna criteria. According to these criteria, a diagnosis of POR requires meeting at least two out of the following three conditions: ① Advanced maternal age (≥ 40 years) or other risk factors for poor ovarian response; ② Previous history of poor ovarian response (≤ 3 oocytes retrieved with conventional stimulation); ③ Abnormal results in ovarian reserve tests such as AFC < 5-7, AMH < 0.5-1.1 ng/ml, or FSH > 8.5 mIU/mL (Kim 2021).  If a patient does not meet the criteria for advanced age or has normal results in ovarian reserve tests, those who experience poor ovarian response in at least two previous maximum stimulation cycles can also be classified as poor responders. Additional criteria identified in literature searches before 2015 include: fewer than 4 mature follicles in the first IVF cycle, FSH levels of 10-15 mIU/ml, and estradiol levels below 1500/600 pg/ml. | |
| Desirable Effects  How substantial are the desirable anticipated effects? | | |
| Judgement | Research evidence | Additional considerations |
| ○ Trivial ●Small ○ Moderate ○ Large ○ Varies ○ Don't know | GnRHa+hCG compared to hCG Table 1: Normal Ovarian Responses Outcome 6 **Outcomes** \| **№ of participants (studies) Follow-up** \| **Certainty of the evidence (GRADE)** \| **Relative effect (95% CI)** \| **Anticipated absolute effects^*^ (95% CI)** \|; \| \| \| \| **Risk with hCG** \| **Risk difference with GnRHa+hCG**; Cumulative live birth rate - NOR, favor I \| 149 (1 RCT) \| ⨁⨁◯◯ Low^a^ \| **RR 1.39** (0.92 to 2.11) \| Study population \|; \| \| \| \| 320 per 1,000 \| **125 more per 1,000** (26 fewer to 355 more); Live birth rate-NOR, favor I \| 478 (3 RCTs) \| ⨁⨁⨁◯ Moderate^b^ \| **RR 1.37** (0.97 to 1.95) \| Study population \|; \| \| \| \| 275 per 1,000 \| **102 more per 1,000** (8 fewer to 262 more); Implantation rate - NOR, favor I \| 1689 (7 RCTs) \| ⨁◯◯◯ Very low^c,d^ \| **RR 1.25** (0.98 to 1.61) \| Study population \|; \| \| \| \| 213 per 1,000 \| **53 more per 1,000** (4 fewer to 130 more); Clinical pregnancy rate-NOR, favor I \| 1149 (8 RCTs) \| ⨁⨁⨁◯ Moderate^c^ \| **RR 1.20** (1.03 to 1.39) \| Study population \|; \| \| \| \| 351 per 1,000 \| **70 more per 1,000** (11 more to 137 more); Metaphase II oocytes-NOR, favor I \| 1226 (8 RCTs) \| ⨁⨁⨁◯ Moderate^e^ \| - \| The mean metaphase II oocytes-NOR, favor I was **0** \| MD **0.82 higher** (0.2 higher to 1.44 higher); Miscarriage rate-NOR, favor I \| 444 (5 RCTs) \| ⨁◯◯◯ Very low^a,e^ \| **RR 0.73** (0.37 to 1.42) \| Study population \|; \| \| \| \| 118 per 1,000 \| **32 fewer per 1,000** (75 fewer to 50 more) a. Downgraded by two levels due to imprecision: low event rate and effect could be both beneficial and harmful. b. Downgraded by one level due to imprecision: low event rate. c. Downgraded by one level due to imprecision: the effect could be both beneficial and harmful. d. Included studies have the potential risk of selection bias (weight >20%). e. Downgraded by one level due to inconsistency: the point estimates are in different directions across included studies (weight >20%). Table 2: Poor Ovarian Responses Outcome 2 **Outcomes** \| **№ of participants (studies) Follow-up** \| **Certainty of the evidence (GRADE)** \| **Relative effect (95% CI)** \| **Anticipated absolute effects^*^ (95% CI)** \|; \| \| \| \| **Risk with hCG** \| **Risk difference with GnRHa+hCG**; Clinical pregnancy rate - POR \| 82 (1 RCT) \| ⨁◯◯◯ Very low^a,b^ \| **RR 1.86** (0.83 to 4.18) \| Study population \|; \| \| \| \| 171 per 1,000 \| **147 more per 1,000** (29 fewer to 543 more); Miscarriage rate - POR \| 82 (1 RCT) \| ⨁◯◯◯ Very low^a,b^ \| **RR 0.56** (0.20 to 1.52) \| Study population \|; \| \| \| \| 220 per 1,000 \| **97 fewer per 1,000** (176 fewer to 114 more) a. Included studies have the potential risk of selection bias (weight >20%). b. Downgraded by two levels due to imprecision: low event rate and effect could be both beneficial and harmful. GnRHa compared to hCG Table 1: Normal Ovarian Responses Outcome 2 **Outcomes** \| **№ of participants (studies) Follow-up** \| **Certainty of the evidence (GRADE)** \| **Relative effect (95% CI)** \| **Anticipated absolute effects^*^ (95% CI)** \|; \| \| \| \| **Risk with hCG** \| **Risk difference with GnRHa**; Metaphase II oocytes-NOR \| 372 (2 RCTs) \| ⨁◯◯◯ Very low^a,b^ \| - \| The mean metaphase II oocytes-NOR was **0** \| MD **0.35 higher** (0.19 lower to 0.88 higher); Endometrial thickness (cm) day after hCG/GnRH-NOR, favor GnRHa \| 179 (1 RCT) \| ⨁◯◯◯ Very low^a,c^ \| - \| The mean endometrial thickness (cm) day after hCG/GnRH-NOR, favor GnRHa was **0** \| MD **0.01 higher** (0 to 0.02 higher) a. Included studies have the potential risk of selection bias (weight >20%). b. Downgraded by two levels due to imprecision: low event rate and effect could be both beneficial and harmful. c. Downgraded by two levels due to imprecision: low sample size and the effect could be both beneficial and harmful. | |
| Undesirable Effects  How substantial are the undesirable anticipated effects? | | |
| Judgement | Research evidence | Additional considerations |
| ○ Large ○ Moderate ●Small ○Trivial ○ Varies ○ Don't know | GnRHa+hCG compared to hCG Table 1: Normal Ovarian Responses Outcome 1 **Outcomes** \| **№ of participants (studies) Follow-up** \| **Certainty of the evidence (GRADE)** \| **Relative effect (95% CI)** \| **Anticipated absolute effects^*^ (95% CI)** \|; \| \| \| \| **Risk with hCG** \| **Risk difference with GnRHa+hCG**; Endometrial thickness on the day of hCG, mm-NOR, favor c \| 491 (3 RCTs) \| ⨁◯◯◯ Very low^a,b,c^ \| - \| The mean endometrial thickness on the day of hCG, mm-NOR, favor c was **0** \| MD **0.04 lower** (0.48 lower to 0.39 higher) a. Included studies have the potential risk of selection bias (weight >20%). b. Downgraded by one level due to inconsistency: the point estimates are in different directions across included studies (weight >20%). c. Downgraded by one level due to imprecision: the effect could be both beneficial and harmful. GnRHa compared to hCG Table 1: Normal Ovarian Responses Outcome 3 **Outcomes** \| **№ of participants (studies) Follow-up** \| **Certainty of the evidence (GRADE)** \| **Relative effect (95% CI)** \| **Anticipated absolute effects^*^ (95% CI)** \|; \| \| \| \| **Risk with hCG** \| **Risk difference with GnRHa**; Live birth rate-NOR, favor GnRHa \| 44 (1 RCT) \| ⨁◯◯◯ Very low^a,b^ \| **RR 1.46** (0.37 to 5.78) \| Study population \|; \| \| \| \| 130 per 1,000 \| **60 more per 1,000** (82 fewer to 623 more); Clinical pregnancy rate-NOR, favor HCG \| 291 (2 RCTs) \| ⨁⨁⨁◯ Moderate^c,d^ \| **RR 0.38** (0.07 to 2.18) \| Study population \|; \| \| \| \| 357 per 1,000 \| **221 fewer per 1,000** (332 fewer to 421 more); Miscarriage rate-NOR, favor HCG \| 164 (4 RCTs) \| ⨁⨁◯◯ Low^a,d^ \| **RR 4.42** (1.10 to 17.67) \| Study population \|; \| \| \| \| 124 per 1,000 \| **423 more per 1,000** (12 more to 2,062 more) a. Included studies have the potential risk of selection bias (weight >20%). b. Downgraded by two levels due to imprecision: low event rate and effect could be both beneficial and harmful. c. Although included studies have a high risk of performance bias, the outcome is unlikely to be affected. d. Downgraded by one levels due to imprecision: low event rate. | |
| Certainty of evidence  What is the overall certainty of the evidence of effects? | | |
| Judgement | Research evidence | Additional considerations |
| ● Very low ○ Low ○ Moderate ○ High ○ No included studies | In the normal ovarian response population, the quality of evidence comparing the use of GnRHa trigger combined with HCG versus HCG trigger alone is moderate to very low. According to the GRADE guidelines, the overall quality of evidence is very low.  In the low ovarian response population, the quality of evidence comparing the use of GnRHa trigger combined with HCG versus HCG trigger alone is very low. According to the GRADE guidelines, the overall quality of evidence is very low.  The quality of evidence comparing GnRHa trigger versus HCG trigger alone is moderate to very low, and according to the GRADE guidelines, the overall quality of evidence is very low. | |
| Values  Is there important uncertainty about or variability in how much people value the main outcomes? | | |
| Judgement | Research evidence | Additional considerations |
| ○ Important uncertainty or variability ○ Possibly important uncertainty or variability ● Probably no important uncertainty or variability ○ No important uncertainty or variability | No relevant patient values were identified.  The importance of outcome indicators in this guideline was determined through voting and discussions among a panel of clinical experts who considered the significance of clinical outcomes. Following discussions during the guideline meetings, clinical experts concluded that there may be no significant uncertainty or variability. | |
| Balance of effects  Does the balance between desirable and undesirable effects favor the intervention or the comparison? | | |
| Judgement | Research evidence | Additional considerations |
| ○ Favors the comparison ○ Probably favors the comparison ○ Does not favor either the intervention or the comparison ● Probably favors the intervention ○ Favors the intervention ○ Varies ○ Don't know | Eleven studies compared adding GnRHa trigger to HCG with using HCG alone in people with NOR，very low certainty evidence indicates the benefits of using a GnRH-a trigger with HCG includes probable increased live birth rates (102 per 1000, RCT=3, n=478, RR=1.37, 95% CI 0.97–1.95), cumulative live birth rates (125 per 1000, RCT=1, n=149, RR=1.39, 95% CI 0.92–2.11), implantation rates (53 per 1000, RCT=7, n=1689, RR=1.25, 95% CI 0.98–1.61), clinical pregnancy rates (70 per 1000, RCT=8, n=1149, RR=1.20, 95% CI 1.03–1.39), and number of MII oocytes (0.82, RCT=8, n=1226, MD=0.82, 95% CI 0.20–1.44). It may also reduce miscarriage rates by 32 per 1000 (RCT=5, n=444, RR=0.73, 95% CI 0.37–1.42). The only harm noted was a slight reduction in endometrial thickness (0.04 mm, RCT=3, n=491, MD=-0.04, 95% CI -0.48–0.39). Overall, the GDG feels the current evidence may indicate more benefit than harms, albeit the certainty of evidence under this comparison is very low.  In people with POR，the intervention may increase clinical pregnancy rate by an average of 147 per 1000（RCT=1, n=82, RR=1.86 95% CI 0.83–4.18），and reduce miscarriage rate by 97 per 1000（RCT=1, n=82, RR=0.56 95% CI 0.20–1.52）. There appears no obvious benefit of the intervention in people with HOR.  When comparing GnRHa and HCG alone, in people with NOR，the number of MII oocytes may increase by 0.35 (RCT=2, n=372, MD=0.35, 95% CI -0.19–0.88), and endometrial thickness may increase by 0.01 mm (RCT=1, n=179, MD=0.01, 95% CI 0.00–0.02). The potential harm is reflected in reduced implantation rate by 214 per 1000 people (RCT=2, n=398, RR=0.31, 95% CI 0.03–3.04), a reduced clinical pregnancy rate by 221 per 1000 people (RCT=2, n=291, RR=0.38, 95% CI 0.07–2.18), and an increased miscarriage rate by 423 per 1000 people (RCT=4, n=164, RR=4.42, 95% CI 1.10–17.67). Overall, the harm of using GnRHa alone in people with NOR probably outweighs benefits (very low certainty evidence). The evidence in HOR patient is lacking. | |
| Resources required  How large are the resource requirements (costs)? | | |
| Judgement | Research evidence | Additional considerations |
| ○ Large costs ● Moderate costs ○ Negligible costs and savings ○ Moderate savings ○ Large savings ○ Varies ○ Don't know | This guideline did not conduct a systematic search on resource requirements. Practical searches did not uncover evidence regarding additional resources needed for relevant fixed protocols. After discussion, experts unanimously agreed that the use of additional GnRHa triggers or substituting GnRHa triggers for HCG triggers may slightly increase the required stimulant, but remains within acceptable limits. | |
| Certainty of evidence of required resources  What is the certainty of the evidence of resource requirements (costs)? | | |
| Judgement | Research evidence | Additional considerations |
| ○ Very low ○ Low ○ Moderate ○ High ● No included studies | Not applicable. | |
| Cost effectiveness  Does the cost-effectiveness of the intervention favor the intervention or the comparison? | | |
| Judgement | Research evidence | Additional considerations |
| ○ Favors the comparison ○ Probably favors the comparison ○ Does not favor either the intervention or the comparison ○ Probably favors the intervention ○ Favors the intervention ○ Varies ● No included studies | No relevant evidence. | |
| Equity  What would be the impact on health equity? | | |
| Judgement | Research evidence | Additional considerations |
| ○ Reduced ○ Probably reduced ● Probably no impact ○ Probably increased ○ Increased ○ Varies ○ Don't know | The experts discussed equity focusing primarily on whether the additional use of GnRHa triggers or the substitution of GnRHa triggers for HCG triggers in different regions of China would lead to unfairness. That is, whether different populations in different regions can access the recommended interventions of this guideline under circumstances beyond their control. | |
| Acceptability  Is the intervention acceptable to key stakeholders? | | |
| Judgement | Research evidence | Additional considerations |
| ○ No ○ Probably no ● Probably yes ○ Yes ○ Varies ○ Don't know | The experts discussed acceptability, primarily focusing on whether the additional use of GnRHa triggers or substituting GnRHa triggers for HCG triggers would be acceptable among the Chinese population. This includes considerations of willingness to pay the price and accept the mode of administration, among other factors. | |
| Feasibility  Is the intervention feasible to implement? | | |
| Judgement | Research evidence | Additional considerations |
| ○ No ○ Probably no ● Probably yes ○ Yes ○ Varies ○ Don't know | The experts discussed acceptability, primarily focusing on whether the additional use of GnRHa triggers or substituting GnRHa triggers for HCG triggers would be acceptable among the Chinese population. This includes considerations of the willingness to pay the price, the method of administration, and other factors. | |

# PICO 6 For individuals undergoing fresh embryo transfer with GnRHa trigger, is reinforced luteal support recommended?

## **Table 6.** **Recommedations**

| Problem  Is the problem a priority? | | |
| --- | --- | --- |
| Judgement | Research evidence | Additional considerations |
| ○ No ○ Probably no ○ Probably yes ● Yes ○ Varies ○ Don't know | GnRH antagonists were introduced into the protocols for ovarian stimulation in assisted reproductive technology (ART) in the 1990s. These antagonists reversibly bind to GnRH receptors in the pituitary gland, rapidly suppressing the release of endogenous GnRH. This allows for a quick inhibition of pituitary function within a short time frame (1-2 hours), preventing premature LH surges while maintaining pituitary responsiveness. Due to these advantages, there has been a gradual increase in the clinical demand for GnRH antagonists in assisted reproductive therapies.  In response to these benefits, expert consensus guidelines for the clinical use of GnRH antagonists were established by the Chinese Medical Association's Reproductive Medicine Branch in 2015, and evidence-based guidelines on ovarian stimulation protocols were issued by the European Society of Reproductive Medicine in 2020. However, specific guidance on the use and management of GnRH antagonists in IVF treatment was lacking in existing guidelines. Consequently, the Reproductive Medicine Specialty Committee of the Chinese Medical Doctor Association initiated the development of clinical management guidelines for the use of GnRH antagonists in IVF treatment. These guidelines provide tailored recommendations for different ovarian response populations.  Poor ovarian reserve/response (POR): Currently, the internationally recognized criteria for POR are defined by the Bologna criteria. According to these criteria, a diagnosis of POR requires meeting at least two out of the following three conditions: ① Advanced maternal age (≥ 40 years) or other risk factors for poor ovarian response; ② Previous history of poor ovarian response (≤ 3 oocytes retrieved with conventional stimulation); ③ Abnormal results in ovarian reserve tests such as AFC < 5-7, AMH < 0.5-1.1 ng/ml, or FSH > 8.5 mIU/mL (Kim 2021).  If a patient does not meet the criteria for advanced age or has normal results in ovarian reserve tests, those who experience poor ovarian response in at least two previous maximum stimulation cycles can also be classified as poor responders. Additional criteria identified in literature searches before 2015 include: fewer than 4 mature follicles in the first IVF cycle, FSH levels of 10-15 mIU/ml, and estradiol levels below 1500/600 pg/ml. | |
| Desirable Effects  How substantial are the desirable anticipated effects? | | |
| Judgement | Research evidence | Additional considerations |
| ○ Trivial ○ Small ○ Moderate ● Large ○ Varies ○ Don't know | Outcome 2 **Outcomes** \| **№ of participants (studies) Follow-up** \| **Certainty of the evidence (GRADE)** \| **Relative effect (95% CI)** \| **Anticipated absolute effects^*^ (95% CI)** \|; \| \| \| \| **Risk with Standard luteal support** \| **Risk difference with Enhanced luteal support**; Ongoing pregnancies: favour I \| 115 (1 non-randomised study) \| ⨁◯◯◯ Very low^a,b^ \| **RR 1.98** (1.16 to 3.37) \| Study population \|; \| \| \| \| 253 per 1,000 \| **248 more per 1,000** (40 more to 599 more); Early pregnancy losses: favour I \| 115 (1 non-randomised study) \| ⨁◯◯◯ Very low^a,c^ \| **RR 0.36** (0.12 to 1.06) \| Study population \|; \| \| \| \| 352 per 1,000 \| **225 fewer per 1,000** (309 fewer to 21 more) a. Downgraded by 1 level due to ROB: A retrospective cohort study, non-randomized controlled study. b. Downgraded by 1 level due to imprecision: low event rate. c. Downgraded by 2 levels due to imprecision: wide confidence interval and low event rate. | |
| Undesirable Effects  How substantial are the undesirable anticipated effects? | | |
| Judgement | Research evidence | Additional considerations |
| ○ Trivial ● Small ○ Moderate ○ Large ○ Varies ○ Don't know | Outcome 1 **Outcomes** \| **№ of participants (studies) Follow-up** \| **Certainty of the evidence (GRADE)** \| **Relative effect (95% CI)** \| **Anticipated absolute effects^*^ (95% CI)** \|; \| \| \| \| **Risk with Standard luteal support** \| **Risk difference with Enhanced luteal support**; Oocytes retrieved \| 115 (1 non-randomised study) \| ⨁◯◯◯ Very low^a,b^ \| - \| The mean oocytes retrieved was **0** \| MD **1.7 lower** (6.42 lower to 3.02 higher) a. Downgraded by 1 level due to ROB: A retrospective cohort study, non-randomized controlled study. b. Downgraded by 2 levels due to imprecision: wide confidence interval and small sample size. | |
| Certainty of evidence  What is the overall certainty of the evidence of effects? | | |
| Judgement | Research evidence | Additional considerations |
| ● Very low ○ Low ○ Moderate ○ High ○ No included studies | According to the GRADE guidelines, the quality of evidence is very low for the use of immediate luteal support compared to luteal support administered 2-5 days after egg retrieval in populations with normal ovarian response. | |
| Values  Is there important uncertainty about or variability in how much people value the main outcomes? | | |
| Judgement | Research evidence | Additional considerations |
| ○ Important uncertainty or variability ● Possibly important uncertainty or variability ○ Probably no important uncertainty or variability ○ No important uncertainty or variability | No relevant patient values were identified.  The importance of outcome measures in this guideline was determined through a vote and discussion among the clinical expert panel after considering the clinical significance of outcomes. Following discussions at the guideline meeting, clinical experts concluded that there may be little or uncertain changes in the importance of outcome measures within patient value preferences. | |
| Balance of effects  Does the balance between desirable and undesirable effects favor the intervention or the comparison? | | |
| Judgement | Research evidence | Additional considerations |
| ○ Favors the comparison ○ Probably favors the comparison ○ Does not favor either the intervention or the comparison ● Probably favors the intervention ○ Favors the intervention ○ Varies ○ Don't know | There lacks evidence regarding enhanced luteal support after an antagonist protocol with an agonist trigger, so GDG cannot provide recommendations. For people with NOR, immediate luteal support post-retrieval may increase ongoing pregnancy rates by 248 per 1000 people (RCT=1, n=115, RR=1.98, 95% CI 1.16–3.37) and reduces early miscarriage rates by 225 per 1000 people (RCT=1, n=115, RR=0.36, 95% CI 0.12–1.06). The intervention appears to have slightly lowered the number of oocytes retrieved (1.7 fewer, RCT=1, n=115, MD=-1.70, 95% CI -6.42–3.02), but unrelated to luteal support timing. | |
| Resources required  How large are the resource requirements (costs)?" | | |
| Judgement | Research evidence | Additional considerations |
| ○ Large costs ○ Moderate costs ● Negligible costs and savings ○ Moderate savings ○ Large savings ○ Varies ○ Don't know | This guideline did not conduct a systematic search on resource requirements. Practical searches did not uncover evidence regarding additional resources needed for relevant fixed protocols.  Following discussions, experts unanimously agreed that providing immediate luteal support after egg retrieval compared to using luteal support 2-5 days later may increase patient costs for luteal support medications (due to extended use for 2-5 days), but the overall increase in resources is deemed acceptably | |
| Certainty of evidence of required resources  What is the certainty of the evidence of resource requirements (costs)? | | |
| Judgement | Research evidence | Additional considerations |
| ○ Very low ○ Low ○ Moderate ○ High ● No included studies | Not applicable. | |
| Cost effectiveness  Does the cost-effectiveness of the intervention favor the intervention or the comparison? | | |
| Judgement | Research evidence | Additional considerations |
| ○ Favors the comparison ○ Probably favors the comparison ○ Does not favor either the intervention or the comparison ○ Probably favors the intervention ○ Favors the intervention ○ Varies ● No included studies | No relevant evidence. | |
| Equity  What would be the impact on health equity? | | |
| Judgement | Research evidence | Additional considerations |
| ○ Reduced ○ Probably reduced ● Probably no impact ○ Probably increased ○ Increased ○ Varies ○ Don't know | After discussions, experts unanimously agreed that providing immediate luteal support after egg retrieval compared to using luteal support 2-5 days later may not affect equity. | |
| Acceptability  Is the intervention acceptable to key stakeholders? | | |
| Judgement | Research evidence | Additional considerations |
| ○ No ○ Probably no ● Probably yes ○ Yes ○ Varies ○ Don't know | After discussions, experts unanimously agreed that key stakeholders generally find immediate luteal support after egg retrieval compared to luteal support administered 2-5 days later to be largely acceptable. No factors were identified that would affect the acceptability of immediate luteal support following egg retrieval. | |
| Feasibility  Is the intervention feasible to implement? | | |
| Judgement | Research evidence | Additional considerations |
| ○ No ○ Probably no ● Probably yes ○ Yes ○ Varies ○ Don't know | After discussions, experts unanimously agreed that providing immediate luteal support after egg retrieval compared to using luteal support 2-5 days later does not require special technical support, thus recommending it may not affect feasibility. | |

# PICO 7 Fresh embryo transfer compared with frozen-all strategy for GnRH-ant protocol?

## **Table 7.** **Recommedations**

| Problem  Is the problem a priority? | | |
| --- | --- | --- |
| Judgement | Research evidence | Additional considerations |
| ○ No ○ Probably no ○ Probably yes ● Yes ○ Varies ○ Don't know | GnRH antagonists were introduced into the protocols for ovarian stimulation in assisted reproductive technology (ART) in the 1990s. These antagonists reversibly bind to GnRH receptors in the pituitary gland, rapidly suppressing the release of endogenous GnRH. This allows for a quick inhibition of pituitary function within a short time frame (1-2 hours), preventing premature LH surges while maintaining pituitary responsiveness. Due to these advantages, there has been a gradual increase in the clinical demand for GnRH antagonists in assisted reproductive therapies.  In response to these benefits, expert consensus guidelines for the clinical use of GnRH antagonists were established by the Chinese Medical Association's Reproductive Medicine Branch in 2015, and evidence-based guidelines on ovarian stimulation protocols were issued by the European Society of Reproductive Medicine in 2020. However, specific guidance on the use and management of GnRH antagonists in IVF treatment was lacking in existing guidelines. Consequently, the Reproductive Medicine Specialty Committee of the Chinese Medical Doctor Association initiated the development of clinical management guidelines for the use of GnRH antagonists in IVF treatment. These guidelines provide tailored recommendations for different ovarian response populations.  Poor ovarian reserve/response (POR): Currently, the internationally recognized criteria for POR are defined by the Bologna criteria. According to these criteria, a diagnosis of POR requires meeting at least two out of the following three conditions: ① Advanced maternal age (≥ 40 years) or other risk factors for poor ovarian response; ② Previous history of poor ovarian response (≤ 3 oocytes retrieved with conventional stimulation); ③ Abnormal results in ovarian reserve tests such as AFC < 5-7, AMH < 0.5-1.1 ng/ml, or FSH > 8.5 mIU/mL (Kim 2021).  If a patient does not meet the criteria for advanced age or has normal results in ovarian reserve tests, those who experience poor ovarian response in at least two previous maximum stimulation cycles can also be classified as poor responders. Additional criteria identified in literature searches before 2015 include: fewer than 4 mature follicles in the first IVF cycle, FSH levels of 10-15 mIU/ml, and estradiol levels below 1500/600 pg/ml. | |
| Desirable Effects  How substantial are the desirable anticipated effects? | | |
| Judgement | Research evidence | Additional considerations |
| ○ Trivial ○ Small ● Moderate ○ Large ○ Varies ○ Don't know | Table1: High Ovarian Responses 1. Overall 1.2 Multiple elective ET Outcome 2 **Outcomes** \| **№ of participants (studies) Follow-up** \| **Certainty of the evidence (GRADE)** \| **Relative effect (95% CI)** \| **Anticipated absolute effects^*^ (95% CI)** \|; \| \| \| \| **Risk with Frozen embryo transfer** \| **Risk difference with Fresh embryo transfer**; Pre-eclampsia \| 861 (1 RCT) \| ⨁⨁⨁◯ Moderate^a,b^ \| RR 0.32 (0.13 to 0.80) \| Study population \|; \| \| \| \| 44 per 1,000 \| 30 fewer per 1,000 (38 fewer to 9 fewer); Gestational hypertension \| 861 (1 RCT) \| ⨁⨁◯◯ Low^a,c^ \| RR 0.51 (0.18 to 1.47) \| Study population \|; \| \| \| \| 23 per 1,000 \| 11 fewer per 1,000 (19 fewer to 11 more) a. Downgraded by 0.5 level due to ROB: there are high risk of bias in blind domains b. Downgraded by 1 levels due to imprecision: very low event rate. c. Downgraded by 2 levels due to imprecision: wide confidence interval and low event rate. Table 2: Normal Ovarian Responses 1. Overall Outcome 2 Outcomes \| № of participants (studies) Follow-up \| Certainty of the evidence (GRADE) \| Relative effect (95% CI) \| Anticipated absolute effects^*^ (95% CI) \|; \| \| \| \| Risk with Frozen embryo transfer-NOR \| Risk difference with Fresh embryo transfer; Pre-eclampsia-favor fresh \| 2226 (3 RCTs) \| ⨁⨁◯◯ Low^a,b^ \| RR 0.64 (0.37 to 1.11) \| Study population \|; \| \| \| \| 40 per 1,000 \| 14 fewer per 1,000 (25 fewer to 4 more); Gestational hypertension-favor fresh \| 2226 (3 RCTs) \| ⨁⨁◯◯ Low^a,b^ \| RR 0.96 (0.50 to 1.85) \| Study population \|; \| \| \| \| 17 per 1,000 \| 1 fewer per 1,000 (9 fewer to 15 more) a. Downgraded by 0.5 level due to ROB: there are high risk of bias in blind domains b. Downgraded by 2 levels due to imprecision: wide confidence interval and low event rate. 1.1 Single elective ET Outcome 2 Outcomes \| № of participants (studies) Follow-up \| Certainty of the evidence (GRADE) \| Relative effect (95% CI) \| Anticipated absolute effects^*^ (95% CI) \|; \| \| \| \| Risk with Frozen embryo transfer-NOR-使用 \| Risk difference with Fresh embryo transfer; Pre-eclampsia \| 1025 (2 RCTs) \| ⨁⨁◯◯ Low^a,b^ \| RR 0.54 (0.17 to 1.71) \| Study population \|; \| \| \| \| 35 per 1,000 \| 16 fewer per 1,000 (29 fewer to 25 more); Gestational hypertension \| 1025 (2 RCTs) \| ⨁⨁◯◯ Low^a,b^ \| RR 0.82 (0.37 to 1.82) \| Study population \|; \| \| \| \| 26 per 1,000 \| 5 fewer per 1,000 (17 fewer to 22 more) a. Downgraded by 0.5 level due to ROB: there are high risk of bias in blind domains b. Downgraded by 2 levels due to imprecision: wide confidence interval and low event rate. 1.2 Multiple elective ET Outcome 4 Outcomes \| № of participants (studies) Follow-up \| Certainty of the evidence (GRADE) \| Relative effect (95% CI) \| Anticipated absolute effects^*^ (95% CI) \|; \| \| \| \| Risk with Frozen embryo transfer-NOR-使用 \| Risk difference with Fresh embryo transfer; Live birth rate-Single vs. Multiple \| 2157 (1 RCT) \| ⨁⨁⨁◯ Moderate^a,b^ \| RR 1.03 (0.95 to 1.12) \| Study population \|; \| \| \| \| 487 per 1,000 \| 15 more per 1,000 (24 fewer to 58 more); Implantation rate \| 3993 (1 RCT) \| ⨁⨁⨁◯ Moderate^a,b^ \| RR 1.03 (0.95 to 1.11) \| Study population \|; \| \| \| \| 411 per 1,000 \| 12 more per 1,000 (21 fewer to 45 more); Clinical pregnancy rate-Single vs. Multiple \| 2157 (1 RCT) \| ⨁⨁⨁◯ Moderate^a,b^ \| RR 1.05 (0.97 to 1.13) \| Study population \|; \| \| \| \| 544 per 1,000 \| 27 more per 1,000 (16 fewer to 71 more); Pre-eclampsia \| 1201 (1 RCT) \| ⨁⨁◯◯ Low^a,c^ \| RR 0.73 (0.41 to 1.30) \| Study population \|; \| \| \| \| 44 per 1,000 \| 12 fewer per 1,000 (26 fewer to 13 more) a. Downgraded by 0.5 level due to ROB: there are high risk of bias in blind domains b. Downgraded by 1 level due to imprecision: wide confidence interval. c. Downgraded by 2 levels due to imprecision: wide confidence interval and low event rate. | |
| Undesirable Effects  How substantial are the undesirable anticipated effects? | | |
| Judgement | Research evidence | Additional considerations |
| ○ Large ○ Moderate ● Small ○ Trivial ○ Varies ○ Don't know | Table1: High Ovarian Responses 1. Overall Outcome 3 **Outcomes** \| **№ of participants (studies) Follow-up** \| **Certainty of the evidence (GRADE)** \| **Relative effect (95% CI)** \| **Anticipated absolute effects^*^ (95% CI)** \|; \| \| \| \| **Risk with Frozen embryo transfer** \| **Risk difference with Fresh embryo transfer**; Moderate, severe risk of OHSS-favor frozen \| 1957 (3 RCTs) \| ⨁◯◯◯ Very low^a,b,c^ \| **RR 3.45** (0.80 to 14.80) \| Study population \|; \| \| \| \| 18 per 1,000 \| **43 more per 1,000** (4 fewer to 242 more); Live birth rate-favor frozen \| 1953 (3 RCTs) \| ⨁⨁⨁⨁ High^a^ \| **RR 0.87** (0.78 to 0.96) \| Study population \|; \| \| \| \| 458 per 1,000 \| **59 fewer per 1,000** (101 fewer to 18 fewer); Clinical pregnancy rate-favor frozen \| 1957 (3 RCTs) \| ⨁⨁⨁◯ Moderate^a,d^ \| **RR 0.96** (0.88 to 1.04) \| Study population \|; \| \| \| \| 550 per 1,000 \| **22 fewer per 1,000** (66 fewer to 22 more) a. Downgraded by 0.5 level due to ROB: there are high risk of bias in blind domains b. Downgraded by one level due to ROB and indirectness: downgraded 0.5 level due to there were high risk of bias in blind domains; downgraded 0.5 level due to heterogeneity. c. Downgraded by 2 levels due to imprecision: wide confidence interval and low event rate. d. Downgraded by 1 level due to imprecision: wide confidence interval. 1.1 Single elective ET Outcome 3 **Outcomes** \| **№ of participants (studies) Follow-up** \| **Certainty of the evidence (GRADE)** \| **Relative effect (95% CI)** \| **Anticipated absolute effects^*^ (95% CI)** \|; \| \| \| \| **Risk with Frozen embryo transfer** \| **Risk difference with Fresh embryo transfer**; Moderate, severe risk of OHSS \| 209 (1 RCT) \| ⨁⨁⨁◯ Moderate^a^ \| **RR 18.82** (1.11 to 319.24) \| Study population \|; \| \| \| \| 0 per 1,000 \| **0 fewer per 1,000** (0 fewer to 0 fewer); Live birth rate \| 205 (1 RCT) \| ⨁⨁◯◯ Low^b^ \| **RR 0.95** (0.68 to 1.32) \| Study population \|; \| \| \| \| 416 per 1,000 \| **21 fewer per 1,000** (133 fewer to 133 more); Clinical pregnancy rate \| 209 (1 RCT) \| ⨁⨁◯◯ Low^b^ \| **RR 0.89** (0.68 to 1.15) \| Study population \|; \| \| \| \| 548 per 1,000 \| **60 fewer per 1,000** (175 fewer to 82 more) a. Downgraded by 1 levels due to imprecision: very low event rate. b. Downgraded by 2 levels due to imprecision: wide confidence interval and low event rate. 1.2 Multiple elective ET **Outcomes** \| **№ of participants (studies) Follow-up** \| **Certainty of the evidence (GRADE)** \| **Relative effect (95% CI)** \| **Anticipated absolute effects^*^ (95% CI)** \|; \| \| \| \| **Risk with Frozen embryo transfer** \| **Risk difference with Fresh embryo transfer**; Moderate, severe risk of OHSS \| 1748 (2 RCTs) \| ⨁◯◯◯ Very low^a,b,c^ \| **RR 2.43** (0.48 to 12.34) \| Study population \|; \| \| \| \| 20 per 1,000 \| **28 more per 1,000** (10 fewer to 222 more); Live birth rate \| 1748 (2 RCTs) \| ⨁⨁⨁⨁ High^a^ \| **RR 0.86** (0.77 to 0.96) \| Study population \|; \| \| \| \| 463 per 1,000 \| **65 fewer per 1,000** (106 fewer to 19 fewer); Clinical pregnancy rate \| 1748 (2 RCTs) \| ⨁⨁⨁◯ Moderate^a,d^ \| **RR 0.96** (0.88 to 1.05) \| Study population \|; \| \| \| \| 550 per 1,000 \| **22 fewer per 1,000** (66 fewer to 28 more) a. Downgraded by 0.5 level due to ROB: there are high risk of bias in blind domains b. Downgraded by 1 level due to indirectness: the I^2^ value is high (I^2^>80%). We explored the clinical, methdological and statistical characteristics of the studies, but the unexplained heterogeneity remained. This potentially reduces our certainty on the observed magnitude of effect. c. Downgraded by 2 levels due to imprecision: wide confidence interval and low event rate. d. Downgraded by 1 level due to imprecision: wide confidence interval. Table1: Normal Ovarian Responses 1. overall **Outcomes** \| **№ of participants (studies) Follow-up** \| **Certainty of the evidence (GRADE)** \| **Relative effect (95% CI)** \| **Anticipated absolute effects^*^ (95% CI)** \|; \| \| \| \| **Risk with Frozen embryo transfer-NOR-使用** \| **Risk difference with Fresh embryo transfer**; Moderate, severe risk of OHSS-favor frozen \| 3807 (2 RCTs) \| ⨁⨁⨁◯ Moderate^a,b^ \| **RR 2.80** (1.41 to 5.56) \| Study population \|; \| \| \| \| 6 per 1,000 \| **10 more per 1,000** (2 more to 26 more); Implantation rate-favor frozen \| 5664 (2 RCTs) \| ⨁⨁◯◯ Low^a,c,d^ \| **RR 0.90** (0.68 to 1.18) \| Study population \|; \| \| \| \| 475 per 1,000 \| **48 fewer per 1,000** (152 fewer to 86 more); Clinical pregnancy rate-favor frozen \| 3879 (3 RCTs) \| ⨁⨁◯◯ Low^a,c,d^ \| **RR 0.94** (0.73 to 1.21) \| Study population \|; \| \| \| \| 574 per 1,000 \| **34 fewer per 1,000** (155 fewer to 121 more); Live birth rate-favor frozen \| 4332 (4 RCTs) \| ⨁⨁⨁◯ Moderate^a,c^ \| **RR 0.92** (0.87 to 0.98) \| Study population \|; \| \| \| \| 481 per 1,000 \| **39 fewer per 1,000** (63 fewer to 10 fewer) a. Downgraded by 0.5 level due to ROB: there are high risk of bias in blind domains b. Downgraded by 1 levels due to imprecision: very low event rate. c. Downgraded by 1 level due to indirectness: the I^2^ value is high (I^2^>80%). We explored the clinical, methdological and statistical characteristics of the studies, but the unexplained heterogeneity remained. This potentially reduces our certainty on the observed magnitude of effect. d. Downgraded by 1 level due to imprecision: wide confidence interval. 1.1 Single elective ET **Outcomes** \| **№ of participants (studies) Follow-up** \| **Certainty of the evidence (GRADE)** \| **Relative effect (95% CI)** \| **Anticipated absolute effects^*^ (95% CI)** \|; \| \| \| \| **Risk with Frozen embryo transfer-NOR-使用** \| **Risk difference with Fresh embryo transfer**; Moderate, severe risk of OHSS \| 1650 (1 RCT) \| ⨁⨁◯◯ Low^a,b^ \| **RR 2.25** (0.70 to 7.28) \| Study population \|; \| \| \| \| 5 per 1,000 \| **6 more per 1,000** (1 fewer to 30 more); Implantation rate \| 1671 (1 RCT) \| ⨁⨁⨁⨁ High^a^ \| **RR 0.78** (0.71 to 0.85) \| Study population \|; \| \| \| \| 625 per 1,000 \| **138 fewer per 1,000** (181 fewer to 94 fewer); Clinical pregnancy rate-D3 vs. D5 \| 1722 (2 RCTs) \| ⨁⨁◯◯ Low^c,d^ \| **RR 0.86** (0.63 to 1.18) \| Study population \|; \| \| \| \| 612 per 1,000 \| **86 fewer per 1,000** (226 fewer to 110 more) a. Downgraded by 0.5 level due to ROB: there are high risk of bias in blind domains b. Downgraded by 2 levels due to imprecision: wide confidence interval and low event rate. c. Downgraded by 1 level due to ROB: there are high risk of bias in blind domains, large sample size and direction inconsistency. d. Downgraded by 1 level due to imprecision: wide confidence interval. 1.2 Multiple elective ET **Outcomes** \| **№ of participants (studies) Follow-up** \| **Certainty of the evidence (GRADE)** \| **Relative effect (95% CI)** \| **Anticipated absolute effects^*^ (95% CI)** \|; \| \| \| \| **Risk with Frozen embryo transfer-NOR-使用** \| **Risk difference with Fresh embryo transfer**; Moderate, severe risk of OHSS \| 2157 (1 RCT) \| ⨁⨁⨁◯ Moderate^a,b^ \| **RR 3.13** (1.34 to 7.31) \| Study population \|; \| \| \| \| 6 per 1,000 \| **14 more per 1,000** (2 more to 41 more); Gestational hypertension \| 1201 (1 RCT) \| ⨁⨁◯◯ Low^a,c^ \| **RR 1.33** (0.43 to 4.18) \| Study population \|; \| \| \| \| 9 per 1,000 \| **3 more per 1,000** (5 fewer to 27 more) a. Downgraded by 0.5 level due to ROB: there are high risk of bias in blind domains b. Downgraded by 1 levels due to imprecision: very low event rate. c. Downgraded by 2 levels due to imprecision: wide confidence interval and low event rate. | |
| Certainty of evidence  What is the overall certainty of the evidence of effects? | | |
| Judgement | Research evidence | Additional considerations |
| ●Very low ○ Low ○ Moderate ○ High ○ No included studies | In the HOR population, among IVF-assisted pregnancy patients treated with COS antagonist protocols, the quality of evidence for key outcome indicators comparing fresh embryo transfer versus frozen embryo transfer is rated as high to very low. According to GRADE guidelines, the overall quality of evidence is very low.  In the NOR, among IVF-assisted pregnancy patients treated with COS antagonist protocols, the quality of evidence for key outcome indicators comparing fresh embryo transfer versus frozen embryo transfer is rated as moderate to low. According to GRADE guidelines, the overall quality of evidence is low. | |
| Values  Is there important uncertainty about or variability in how much people value the main outcomes? | | |
| Judgement | Research evidence | Additional considerations |
| ○ Important uncertainty or variability ○ Possibly important uncertainty or variability ● Probably no important uncertainty or variability ○ No important uncertainty or variability | The importance of outcomes was predefined by all panel members, no evidence was retrieved to support the value of the main outcomes. | |
| Balance of effects  Does the balance between desirable and undesirable effects favor the intervention or the comparison? | | |
| Judgement | Research evidence | Additional considerations |
| ○ Favors the comparison ○ Probably favors the comparison ○ Does not favor either the intervention or the comparison ● Probably favors the intervention ○ Favors the intervention ○ Varies ○ Don't know | In the HOR population, among IVF patients treated with COS antagonist protocols, the benefits of fresh embryo transfer versus frozen embryo transfer are unclear. desirable effects include: significantly increased incidence of OHSS in key outcomes (increase of 43 per 1000 persons, RCT=3, n=1957, RR=3.45, 95% CI 0.80–14.80), reduced live birth rate (decrease of 59 per 1000 persons, RCT=3, n=1953, RR=0.87, 95% CI 0.78–0.96), and reduced clinical pregnancy rate (decrease of 22 per 1000 persons, RCT=3, n=1957, RR=0.96, 95% CI 0.88–1.04).  Subgroup effects indicate: the desirable effects are unclear.  Undesirable effects include reduced live birth rate (decrease of 21 per 1000 persons, RCT=1, n=205, RR=0.95, 95% CI 0.68–1.32), reduced clinical pregnancy rate (decrease of 60 per 1000 persons, RCT=1, n=209, RR=0.89, 95% CI 0.68–1.15), and possibly increased incidence of moderate to severe OHSS, though the results are inconclusive. For cleavage-stage multiple embryo transfer, the benefits of fresh embryo transfer versus frozen embryo transfer are: reduced incidence of preeclampsia in key outcomes (decrease of 30 per 1000 persons, RCT=1, n=861, RR=0.32, 95% CI 0.13–0.80); reduced incidence of pregnancy-induced hypertension in important outcomes (decrease of 11 per 1000 persons, RCT=1, n=861, RR=0.51, 95% CI 0.18–1.47). Harms include: increased incidence of moderate to severe OHSS (increase of 28 per 1000 persons, RCT=2, n=1748, RR=2.43, 95% CI 0.48–12.34), reduced live birth rate (decrease of 65 per 1000 persons, RCT=2, n=1748, RR=0.86, 95% CI 0.77–0.96), and reduced clinical pregnancy rate (decrease of 22 per 1000 persons, RCT=2, n=1748, RR=0.96, 95% CI 0.88–1.05).  In the NOR population, the desirable effects are as follows: in key outcomes, there is a moderate reduction in the incidence of preeclampsia (decrease of 14 per 1000 persons, RCT=3, n=2226, RR=0.64, 95% CI 0.37–1.11) and a slight reduction in the incidence of pregnancy-induced hypertension (decrease of 1 per 1000 persons, RCT=3, n=2226, RR=0.96, 95% CI 0.50–1.85).  The undesirable effects include: in key outcomes, a slight increase in ovarian hyperstimulation syndrome (increase of 10 per 1000 persons, RCT=2, n=3807, RR=2.80, 95% CI 1.41–5.56), moderate reduction in live birth rate (decrease of 39 per 1000 persons, RCT=4, n=4332, RR=0.92, 95% CI 0.87–0.98), moderate reduction in embryo implantation rate (decrease of 48 per 1000 persons, RCT=2, n=5664, RR=0.90, 95% CI 0.68–1.18), and moderate reduction in clinical pregnancy rate (decrease of 34 per 1000 persons, RCT=3, n=3879, RR=0.94, 95% CI 0.73–1.21).  Subgroup effects indicate: in the normal ovarian response population, the benefits of single embryo transfer with blastocyst stage embryos result in a moderate reduction in preeclampsia (decrease of 16 per 1000 persons, RCT=2, n=1025, RR=0.54, 95% CI 0.17–1.71) and a slight reduction in pregnancy-induced hypertension (decrease of 5 per 1000 persons, RCT=2, n=1025, RR=0.82, 95% CI 0.37–1.82). Harms include: slight increase in ovarian hyperstimulation syndrome (increase of 6 per 1000 persons, RCT=1, n=1650, RR=2.25, 95% CI 0.70–7.28), significant reduction in embryo implantation rate (decrease of 138 per 1000 persons, RCT=1, n=1671, RR=0.78, 95% CI 0.71–0.85), significant reduction in clinical pregnancy rate (decrease of 86 per 1000 persons, RCT=2, n=1722, RR=0.86, 95% CI 0.63–1.18). In the subgroup undergoing cleavage-stage multiple embryo transfer, the benefits include slight increase in live birth rate (increase of 15 per 1000 persons, RCT=1, n=2157, RR=1.03, 95% CI 0.95–1.12), moderate increase in clinical pregnancy rate (increase of 27 per 1000 persons, RCT=1, n=2157, RR=1.05, 95% CI 0.97–1.13), slight increase in embryo implantation rate (increase of 12 per 1000 persons, RCT=1, n=3993, RR=1.03, 95% CI 0.95–1.11), and reduction in preeclampsia (decrease of 12 per 1000 persons, RCT=1, n=1201, RR=0.73, 95% CI 0.41–1.30). Undesirable effects include: moderate increase in moderate to severe OHSS in key outcomes (increase of 14 per 1000 persons, RCT=1, n=2157, RR=3.13, 95% CI 1.34–7.31), slight increase in pregnancy-induced hypertension (increase of 3 per 1000 persons, RCT=1, n=1201, RR=1.33, 95% CI 0.43–4.18). | |
| Resources required  How large are the resource requirements (costs)? | | |
| Judgement | Research evidence | Additional considerations |
| ○ Large costs ○ Moderate costs ● Negligible costs and savings ○ Moderate savings ○ Large savings ○ Varies ○ Don't know | A retrospective cohort study found that in patients undergoing frozen embryo transfer compared to fresh embryo transfer, the average per-person cost (6952 and 6863 euros respectively) and the cost per live birth (13,101 and 15,279 euros respectively) showed no statistically significant differences. Although embryo freezing incurs additional costs such as embryo cryopreservation and thawing, reducing the number of transfer cycles may potentially lower overall expenses. | |
| Certainty of evidence of required resources  What is the certainty of the evidence of resource requirements (costs)? | | |
| Judgement | Research evidence | Additional considerations |
| ○ Very low ○ Low ○ Moderate ○ High ● No included studies | Not applicable. | |
| Cost effectiveness  Does the cost-effectiveness of the intervention favor the intervention or the comparison? | | |
| Judgement | Research evidence | Additional considerations |
| ○ Favors the comparison ○ Probably favors the comparison ● Does not favor either the intervention or the comparison ○ Probably favors the intervention ○ Favors the intervention ○ Varies ○ No included studies | LE and colleagues conducted a randomized controlled trial comparing frozen embryo transfer (FET) with fresh embryo transfer (ET) in non-PCOS patients and performed a cost-effectiveness analysis. The results showed no statistically significant differences between the two groups in terms of live birth rates and average costs per couple.  A study from Brazil indicated the following costs: For each ongoing pregnancy, fresh embryo transfer compared to full-cycle frozen embryo transfer cost 23,040.33 ± 2,323.27 vs. 22,742.04 ± 1,006.66 (p = 0.040). For the entire treatment process, fresh embryo transfer compared to full-cycle frozen embryo transfer cost 7,417.54 ± 747.95 vs. 9,020.58 ± 399.29 (p = 0.001). | |
| Equity  What would be the impact on health equity? | | |
| Judgement | Research evidence | Additional considerations |
| ○ Reduced ○ Probably reduced ● Probably no impact ○ Probably increased ○ Increased ○ Varies ○ Don't know | The discussion centered on fairness, primarily addressing whether the use of fresh embryo transfer versus frozen embryo transfer in antagonist therapy across different regions in China could lead to inequity. Specifically, whether different populations in various regions could access recommended interventions outlined in the guidelines under similar circumstances was examined. | |
| Acceptability  Is the intervention acceptable to key stakeholders? | | |
| Judgement | Research evidence | Additional considerations |
| ○ No ○ Probably no ● Probably yes ○ Yes ○ Varies ○ Don't know | The experts discussed the acceptability of using fresh embryo transfer compared to frozen embryo transfer in antagonist therapy among the Chinese population, including aspects such as willingness to pay for this treatment and the mode of administration. | |
| Feasibility  Is the intervention feasible to implement? | | |
| Judgement | Research evidence | Additional considerations |
| ○ No ○ Probably no ● Probably yes ○ Yes ○ Varies ○ Don't know | The experts discussed the feasibility of using fresh embryo transfer compared to frozen embryo transfer in antagonist therapy in China, whether specific technical personnel are needed for medical interventions, and whether the required technology exists. | |

# PICO 8 Which is the preferred timing to perform the embryo transfer: Day 3 or Day 5?

## **Table 8.** **Recommedations**

| Problem  Is the problem a priority? | | |
| --- | --- | --- |
| Judgement | Research evidence | Additional considerations |
| ○ No ○ Probably no ○ Probably yes ● Yes ○ Varies ○ Don't know | GnRH antagonists were introduced into the protocols for ovarian stimulation in assisted reproductive technology (ART) in the 1990s. These antagonists reversibly bind to GnRH receptors in the pituitary gland, rapidly suppressing the release of endogenous GnRH. This allows for a quick inhibition of pituitary function within a short time frame (1-2 hours), preventing premature LH surges while maintaining pituitary responsiveness. Due to these advantages, there has been a gradual increase in the clinical demand for GnRH antagonists in assisted reproductive therapies.  In response to these benefits, expert consensus guidelines for the clinical use of GnRH antagonists were established by the Chinese Medical Association's Reproductive Medicine Branch in 2015, and evidence-based guidelines on ovarian stimulation protocols were issued by the European Society of Reproductive Medicine in 2020. However, specific guidance on the use and management of GnRH antagonists in IVF treatment was lacking in existing guidelines. Consequently, the Reproductive Medicine Specialty Committee of the Chinese Medical Doctor Association initiated the development of clinical management guidelines for the use of GnRH antagonists in IVF treatment. These guidelines provide tailored recommendations for different ovarian response populations.  Poor ovarian reserve/response (POR): Currently, the internationally recognized criteria for POR are defined by the Bologna criteria. According to these criteria, a diagnosis of POR requires meeting at least two out of the following three conditions: ① Advanced maternal age (≥ 40 years) or other risk factors for poor ovarian response; ② Previous history of poor ovarian response (≤ 3 oocytes retrieved with conventional stimulation); ③ Abnormal results in ovarian reserve tests such as AFC < 5-7, AMH < 0.5-1.1 ng/ml, or FSH > 8.5 mIU/mL (Kim 2021).  If a patient does not meet the criteria for advanced age or has normal results in ovarian reserve tests, those who experience poor ovarian response in at least two previous maximum stimulation cycles can also be classified as poor responders. Additional criteria identified in literature searches before 2015 include: fewer than 4 mature follicles in the first IVF cycle, FSH levels of 10-15 mIU/ml, and estradiol levels below 1500/600 pg/ml. | |
| Desirable Effects  How substantial are the desirable anticipated effects? | | |
| Judgement | Research evidence | Additional considerations |
| ○ Trivial ● Small ○ Moderate ○ Large ○ Varies ○ Don't know | Table 1: Cleavage embryo transfer compared to Blastocyst embryo transfer for IVF assisted pregnancy patients Outcomes 1 **Outcomes** \| **№ of participants (studies) Follow-up** \| **Certainty of the evidence (GRADE)** \| **Relative effect (95% CI)** \| **Anticipated absolute effects^*^ (95% CI)** \|; \| \| \| \| **Risk with Blastocyst embryo transfer** \| **Risk difference with Cleavage embryo transfer**; Multiple births \| 99 (1 RCT) \| ⨁⨁◯◯ Low^a^ \| **RR 7.02** (0.35 to 142.56) \| Study population \|; \| \| \| \| 0 per 1,000 \| **0 fewer per 1,000** (0 fewer to 0 fewer) a. Downgraded by two level due to imprecision: wide confidence interval and low event rate. | |
| Undesirable Effects  How substantial are the undesirable anticipated effects? | | |
| Judgement | Research evidence | Additional considerations |
| ○ Trivial ○ Small ● Moderate ○ Large ○ Varies ○ Don't know | Table 1: Cleavage embryo transfer compared to Blastocyst embryo transfer for IVF assisted pregnancy patients Outcomes 2 **Outcomes** \| **№ of participants (studies) Follow-up** \| **Certainty of the evidence (GRADE)** \| **Relative effect (95% CI)** \| **Anticipated absolute effects^*^ (95% CI)** \|; \| \| \| \| **Risk with Blastocyst embryo transfer** \| **Risk difference with Cleavage embryo transfer**; Clinical pregnancy rate \| 351 (1 RCT) \| ⨁⨁⨁◯ Moderate^a^ \| **RR 0.70** (0.50 to 0.99) \| Study population \|; \| \| \| \| 331 per 1,000 \| **99 fewer per 1,000** (166 fewer to 3 fewer); Ectopic pregnancy \| 132 (1 RCT) \| ⨁⨁◯◯ Low^b^ \| **RR 1.24** (0.08 to 19.36) \| Study population \|; \| \| \| \| 14 per 1,000 \| **3 more per 1,000** (13 fewer to 252 more) a. Downgraded by one level due to imprecision: low event rate. b. Downgraded by two level due to imprecision: wide confidence interval and low event rate. | |
| Certainty of evidence  What is the overall certainty of the evidence of effects? | | |
| Judgement | Research evidence | Additional considerations |
| ○ Very low ○ Low ● Moderate ○ High ○ No included studies | In IVF patients undergoing treatment with COS and antagonist regimen, the evidence quality for key outcome indicators comparing D3 embryo transfer to D5 embryo transfer is moderate, with an overall moderate quality of evidence. | |
| Values  Is there important uncertainty about or variability in how much people value the main outcomes? | | |
| Judgement | Research evidence | Additional considerations |
| ○ Important uncertainty or variability ○ Possibly important uncertainty or variability ● Probably no important uncertainty or variability ○ No important uncertainty or variability | No relevant patient values were identified.  The importance of outcome indicators in this guideline was determined through voting and discussions among a panel of clinical experts who considered the significance of clinical outcomes. Following discussions during the guideline meetings, clinical experts concluded that there may be no significant uncertainty or variability. | |
| Balance of effects  Does the balance between desirable and undesirable effects favor the intervention or the comparison? | | |
| Judgement | Research evidence | Additional considerations |
| ○ Favors the comparison ○ Probably favors the comparison ○ Does not favor either the intervention or the comparison ● Probably favors the intervention ○ Favors the intervention ○ Varies ○ Don't know | In IVF patients undergoing COS with an antagonist protocol，the benefits of D3 embryo transfer compared to D5 embryo transfer are unknown. However, D3 embryo transfer appears to reduce the clinical pregnancy rate by 99 per 1000 people (RCT=1, n=351, RR=0.70, 95% CI 0.50–0.99) and slightly increases the risk of ectopic pregnancy by 3 per 1000 people (RCT=1, n=132, RR=1.24, 95% CI 0.08–19.36). The multiple pregnancy rate is unclear.  After discussion, experts unanimously agreed that in IVF patients undergoing treatment with COS and antagonist regimen, there is a preference for D5 embryo transfer due to desirable and undesirable effects. | |
| Resources required  How large are the resource requirements (costs)?" | | |
| Judgement | Research evidence | Additional considerations |
| ○ Large costs ○ Moderate costs ● Negligible costs and savings ○ Moderate savings ○ Large savings ○ Varies ○ Don't know | This guideline did not systematically search for resource requirements. Practical searches did not find evidence regarding additional resources needed for D3 embryo transfer compared to D5 embryo transfer. Following discussion, experts unanimously agreed that using D3 embryo transfer compared to D5 embryo transfer results in a minor increase in resource requirements that is deemed acceptable. | |
| Certainty of evidence of required resources  What is the certainty of the evidence of resource requirements (costs)? | | |
| Judgement | Research evidence | Additional considerations |
| ○ Very low ○ Low ○ Moderate ○ High ● No included studies | Not applicable. | |
| Cost effectiveness  Does the cost-effectiveness of the intervention favor the intervention or the comparison? | | |
| Judgement | Research evidence | Additional considerations |
| ○ Favors the comparison ○ Probably favors the comparison ○ Does not favor either the intervention or the comparison ○ Probably favors the intervention ○ Favors the intervention ○ Varies ● No included studies | No relevant evidence. | |
| Equity  What would be the impact on health equity? | | |
| Judgement | Research evidence | Additional considerations |
| ○ Reduced ○ Probably reduced ● Probably no impact ○ Probably increased ○ Increased ○ Varies ○ Don't know | After discussion, experts unanimously agreed that D3 embryo transfer compared to D5 embryo transfer may not have an impact on equity. | |
| Acceptability  Is the intervention acceptable to key stakeholders? | | |
| Judgement | Research evidence | Additional considerations |
| ○ No ○ Probably no ● Probably yes ○ Yes ○ Varies ○ Don't know | After discussion, experts unanimously agreed that D3 embryo transfer compared to D5 embryo transfer may not have an impact on acceptability. | |
| Feasibility  Is the intervention feasible to implement? | | |
| Judgement | Research evidence | Additional considerations |
| ○ No ○ Probably no ● Probably yes ○ Yes ○ Varies ○ Don't know | After discussion, experts unanimously agreed that D3 embryo transfer compared to D5 embryo transfer may not have an impact on feasibility. | |
