# Supplementary material for: Recommendations for the management of GnRH‐ant for ovarian stimulation in the assistant reproductive process: Benefits and harms for different ovarian response population
Source: Clin Public Health Guidel. 2025 May 21;2(3):e70022. doi: 10.1002/gin2.70022 (PMC13563367; doi:10.1002/gin2.70022)
Supplement: Supplementary file 2 [file GIN2-2-e70022-s003.docx]

Contents

[PICO 1 Whether the GnRH-ant protocol is recommended over the GnRH agonist (GnRH-a) protocols?	1](#_Toc176871314)

[PICO 2 Among patients undergoing IVF assisted reproduction with COS using GnRH-ant, is it recommended to implement pretreatments?	2](#_Toc176871315)

[PICO 3 Should fixed regimen or flexible regimen be preferred under GnRH-ant protocol?	3](#_Toc176871316)

[PICO 4 Is early initiation of GnRH-ant during stimulation recommended?	4](#_Toc176871317)

[PICO 5 Is the GnRHa add-on trigger recommended over the HCG trigger alone?	5](#_Toc176871318)

[PICO 6 For individuals undergoing fresh embryo transfer with GnRHa trigger, is reinforced luteal support recommended?	6](#_Toc176871319)

[PICO 7 Fresh embryo transfer compared with frozen-all strategy for GnRH-ant protocol?	7](#_Toc176871320)

[PICO 8 Which is the preferred timing to perform the embryo transfer: Day 3 or Day 5?	8](#_Toc176871321)

# PICO 1. Whether the GnRH-ant protocol is recommended over the GnRH agonist (GnRH-a) protocols?

**Identification of studies via databases and registers**

Records removed before screening:

Duplicate records removed (n = 2778)

Records identified through

English database searching (n =6766)

PubMed, n = 1329

Embase, n = 2695

Wos, n = 1536

Cochrane, n = 1206

**Identification**

Records excluded

(n = 3713)

Records screened

(n =3988)

Reports not retrieved (n = 18)

Reports sought for retrieval

(n = 275)

**Screening**

Full-text records excluded (n=241)

with reasons:
1) Not IVF assisted pregnancy patients (n=6)

2) Not GnRH-ant protocol for intervention group (n=3)

3) Not GnRHa protocol for control group (n=5)

4) Not RCT (n=14)

5) Not Chinese or English (n=6)

6) Duplicates (n=19)

7) Published earlier than 2016 (n=176)

8) Title & Abstract only (n=8)

9) Protocol (n=4)

Reports assessed for eligibility

(n = 257)

**Included**

16 Reports of included studies

2 SR (48 Reference)

14 RCT

# PICO 2. Among patients undergoing IVF assisted reproduction with COS using GnRH-ant, is it recommended to implement pretreatments?

**Identification of studies via databases and registers**

Records removed before screening:

Duplicate records removed (n = 2778)

Records identified through

English database searching (n =6766)

PubMed, n = 1329

Embase, n = 2695

Wos, n = 1536

Cochrane, n = 1206

**Identification**

Records screened

(n = 3988)

Records excluded

(n = 3907)

Reports not retrieved

(n = 3)

1) Unpublished data (n = 3)

Reports sought for retrieval

(n = 81)

**Screening**

Full-text records excluded (n=55) with reasons:

1) Not RCT (n=17)

2) Duplicates (n=9)

3) Title & Abstract only (n=21)

4) Comparison between different pretreatment methods (n=4)

5) Comparison between different pretreatment medicine (n=2)

6) Not GnRH-ant protocol (n=1)

7) Protocol (n=1)

Reports assessed for eligibility

(n = 78)

23 Reports of included studies

1 SR (3 references)

22 RCT

**Included**

# PICO 3. Should fixed regimen or flexible regimen be preferred under GnRH-ant protocol?

**Identification of studies via databases and registers**

Records removed before screening:

Duplicate records removed (n =2778)

Records identified through

English database searching (n =6766)

PubMed, n = 1329

Embase, n = 2695

Wos, n = 1536

Cochrane, n = 1206

**Identification**

Records screened

(n = 3988)

Records excluded

(n = 3949)

Reports not retrieved

(n = 0)

Reports sought for retrieval

(n = 39)

**Screening**

Full-text records excluded (n=30) with reasons:

1) Not RCT (n=14)

2) Not IVF assisted pregnancy patients (n=2)

3) Not fixed GnRH-ant protocol for intervention group (n=2)

4) Not flexible GnRH-ant protocol for control group (n=8)

5) Duplicates (n=4)

Reports assessed for eligibility

(n = 39)

9 Reports of included studies

9 RCT (2 Abstract)

**Included**

# PICO 4. Is early initiation of GnRH-ant during stimulation recommended?

**Identification of studies via other methods**

**Identification of studies via databases**

Records removed before screening:

Duplicate records removed (n =2778)

Records identified through

English database searching (n =6766)

PubMed, n = 1329

Embase, n = 2695

Wos, n = 1536

Cochrane, n = 1206

Records identified from:

Articles provided by NCT website

(n = 2)

**Identification**

Records screened

(n = 3988)

Records excluded

(n = 3942)

Reports not retrieved

(n = 0)

Reports not retrieved

(n = 8)

1) Unpublished data (n = 8)

Reports sought for retrieval

(n = 2)

Reports sought for retrieval

(n = 46)

**Screening**

Full-text records excluded (n=32) with reasons:

1) Not RCT (n=11)

2) Not early initiation of GnRH-ant protocol for intervention group (n=18)

3) Duplicates (n=2)

4) Not Chinese or English (n=1)

Reports excluded (n = 2)

1) Duplication (n=2)

Reports assessed for eligibility (n = 0)

Reports assessed for eligibility (n = 38)

Reports of included studies

6 RCT (2 Abstracts)

**Included**

# PICO 5. Is the GnRHa add-on trigger recommended over the HCG trigger alone?

**Identification of studies via databases and registers**

Records removed before screening:

Duplicate records removed (n =482)

Records identified through

English database searching (n = 1489)

PubMed, n = 202

Embase, n = 481

Wos, n = 341

Cochrane, n = 465

**Identification**

Records screened

(n = 1007)

Records excluded

(n = 936)

Reports sought for retrieval

(n = 71)

Reports not retrieved

(n = 0)

**Screening**

Full-text records excluded (n=65) with reasons:

1) Not RCT (n=14)

2) Not IVF assisted pregnancy patients (n=1)

3) Not triggered with HCG for control group (n=1)

4) Duplicates (n=13)

5) Not triggered with GnRH-a for intervention group (n=1)

6) Published earlier than 2020 (n=35)

Reports assessed for eligibility

(n = 71)

7 Reports of included studies

1 SR (12 Reference)

5 RCT

**Included**

# PICO 6. For individuals undergoing fresh embryo transfer with GnRHa trigger, is reinforced luteal support recommended?

**Identification of studies via other methods**

**Identification of studies via databases**

Records identified through

English database searching (n = 1489)

PubMed, n = 202

Embase, n = 481

Wos, n = 341

Cochrane, n = 465

Records identified from:

Articles provided by experts

(n = 13)

Records removed before screening:

Duplicate records removed (n = 482)

**Identification**

Records screened

(n = 1007)

Records excluded

(n = 983)

Reports not retrieved

(n = 0)

Reports not retrieved (n = 0)

Reports sought for retrieval

(n = 13)

Reports sought for retrieval

(n = 24)

**Screening**

Full-text records excluded (n=24) with reasons:

1) Not RCT (n=2)

2) Not triggered with GnRHa (n=10)

3) Without luteal phase support (LPS) for control group (n=9)

4) Unable to extract data (n=3)

Reports excluded (n = 12)

1) Wrong study design (n = 4)
2) Wrong population (n = 1)
3) Wrong intervention (n = 7)

Reports assessed for eligibility (n = 13)

Reports assessed for eligibility (n = 24)

Reports of included studies

1 Cohort

**Included**

# PICO 7. Fresh embryo transfer compared with frozen-all strategy for GnRH-ant protocol?

**Identification of studies via other methods**

**Identification of studies via databases**

Records removed before screening:

Duplicate records removed (n = 480)

Records identified from:

Articles provided by experts

(n = 2)

Records identified through

English database searching (n = 1302)

PubMed, n = 220;

Embase, n = 551;

Wos, n = 365;

Cochrane, n = 166.

**Identification**

Records screened

(n = 822)

Records excluded

(n = 797)

Reports not retrieved

(n = 0)

Reports sought for retrieval

(n = 2)

Reports sought for retrieval

(n = 25)

Reports not retrieved

(n = 0)

**Screening**

Reports excluded (n = 0)

Reports excluded (n = 20):

1) Not RCT (n=8)

2) Not fresh embryo transfer in GnRH-ant protocol for intervention group (n=6)

3) Duplicates (n=4)

4) Title only (n=1)

5) Protocol (n=1)

4) Title only (n=1)

5) Protocol (n=1)

Reports assessed for eligibility (n = 2)

Reports assessed for eligibility (n = 25)

Reports of included studies

(n = 7)

**Included**

# PICO 8. Which is the preferred timing to perform the embryo transfer: Day 3 or Day 5?

**Identification of studies via databases and registers**

Records removed before screening:

Duplicate records removed (n =959)

Records identified through

English database searching (n = 2281)

PubMed, n = 419

Embase, n = 892

Wos, n = 520

Cochrane, n = 450

**Identification**

Records screened

(n = 1322)

Records excluded

(n = 1294)

Reports sought for retrieval

(n = 28)

Reports not retrieved

(n = 0)

**Screening**

Full-text records excluded (n=27) with reasons:

1) Not RCT (n=13)

2) Embryos were not transferred on day 3 for intervention group (n=4)

3) Embryos were not transferred on day 5 for control group(n=7)

4) Not GnRH-ant protocol (n=2)

5) Unable to extract data (n=1)

Reports assessed for eligibility

(n = 28)

1 Reports of included studies

1 RCT

**Included**
