# Supplementary material for: Recommendations for the management of GnRH‐ant for ovarian stimulation in the assistant reproductive process: Benefits and harms for different ovarian response population
Source: Clin Public Health Guidel. 2025 May 21;2(3):e70022. doi: 10.1002/gin2.70022 (PMC13563367; doi:10.1002/gin2.70022)
Supplement: Supplementary file 3 [file GIN2-2-e70022-s004.docx]

**Search strategy**

**Search Date: 2022-12-31**

|  | Pubmed | Embase | Wos | Cochrane |
| --- | --- | --- | --- | --- |
| Number of searches | 128 | 231 | 205 | 33 |
| After deduplication | 126 | 132 | 97 | 24 |
| Total SR | 379 | | | |
| Number of searches | 1201 | 2464 | 1331 | 1173 |
| After deduplication | 1192 | 1543 | 420 | 454 |
| PICO 1-4 RCT | 3609 | | | |
| Number of searches | 74 | 250 | 136 | 432 |
| After deduplication | 74 | 184 | 37 | 333 |
| PICO 5/6 RCT | 628 | | | |
| Number of searches | 92 | 320 | 160 | 133 |
| After deduplication | 92 | 234 | 62 | 55 |
| PICO 7 RCT | 443 | | | |
| Number of searches | 291 | 661 | 315 | 417 |
| After deduplication | 288 | 412 | 78 | 165 |
| PICO 8 RCT | 943 | | | |

PubMed

#1. "Fertilization in Vitro"[Mesh] OR "Reproductive Techniques, Assisted"[Mesh] OR "Ovulation Induction"[Mesh] OR ART[tw] OR "artificial insemination*"[tw] OR "intrauterine insemination*"[tw] OR "embryo disposition*"[tw] OR "Embryo Transfer*"[tw] OR "in vitro fertilization*"[tw] OR "fertilization in vitro"[tw] OR "intracytoplasmic sperm injection*"[tw] OR "intra-cytoplasmic sperm injection*"[tw] OR "ovulation induction*"[tw] OR "oviduct insemination*"[tw] OR IVF[tw] OR ICSI[tw] OR "Ovarian Stimulation*"[tw] OR IUI[tw] OR "uterine insemination*"[tw] OR "Test-Tube Fertilization"[tw] OR "Test-Tube Babies"[tw] OR "Test-Tube Baby"[tw] OR single sperm injection[tw] OR "in-vitro assisted fertilization*"[tw]
#2. "ganirelix"[Supplementary Concept] OR "cetrorelix"[Supplementary Concept] OR "iturelix" [Supplementary Concept] OR "Gonadotropin-releasing hormone antagonist*"[tw] OR "GnRH antagonist*"[tw] OR "GnRH-ant"[tw] OR "GnRH receptor antagonist*"[tw] OR GnRHant[tw] OR ganirelix[tw] OR "RS-26306"[tw] OR Antagon[tw] OR orgalutran[tw] OR cetrorelix[tw] OR "SB-75"[tw] OR Cetrotide[tw] OR iturelix[tw] OR Antide[tw] OR "ORF 23541"[tw]	5269
#3. #1 and #2	2029
#4. "Meta-Analysis"[pt] OR "Meta-Analysis as Topic"[Mesh] OR "Systematic Review" [pt] OR "Systematic Reviews as Topic"[Mesh] OR "systematic"[Filter] OR "Systematic Review*"[tiab] OR "Meta-Analysis"[tiab] OR Metaanalys*[tiab]
#5. ("controlled clinical trial"[pt] OR "Controlled Clinical Trials as Topic"[MeSH] OR "Random Allocation"[MeSH] OR "Double-Blind Method"[MeSH] OR "single-blind method"[MeSH] OR "Control Groups"[MeSH] OR "cross-over studies"[MeSH] OR random*[tiab] OR placebo[tiab] OR trial[tiab] OR groups[tiab] OR crossover[tiab] OR cross-over[tiab]) NOT ("Animals"[Mesh] NOT ("Humans"[Mesh] AND "Animals"[Mesh]))
#6. ("Observational Study"[pt] OR "Observational Studies as Topic"[Mesh] OR "Cohort Studies"[Mesh] OR "Case-Control Studies"[Mesh] OR "Cross-Sectional Studies"[Mesh] OR Observational Stud*[tiab] OR Cohort[tiab] OR "Follow-Up"[tiab] OR Longitudinal*[tiab] OR Prospectiv*[tiab] OR Retrospectiv*[tiab] OR "Case-Control"[tiab] OR "Cross-Sectional"[tiab] OR "case series"[tiab] OR "single arm"[tiab]) NOT ("Animals"[Mesh] NOT ("Humans"[Mesh] AND "Animals"[Mesh]))
#7. #3 and #4	128 ，pico1-8，SR
#8. #3 and #5	1101，pico1-4，RCT
#9. #3 and #6	1008，pico1-8，OS
#10. "GnRHa trigger*"[tw] OR "GnRH agonist trigger*"[tw] OR "gonadotrophin-releasing hormone analogues trigger*"[tw] OR "gonadotrophin-releasing hormone agonist trigger*"[tw] OR "GnRH-a trigger*"[tw]
#11. #8 AND #10	74, PICO5/6, RCT
#12. "fresh embryo*"[tw] OR "frozen embryo*"[tw]
#13. #8 AND #12	92, PICO7, RCT
#14. "cleavage stage"[tw] OR "blastocyst stage"[tw] OR D3[tw] OR D5[tw] OR "3 days"[tw] OR "5 days"[tw] OR "day 3"[tw] OR "day 5"[tw] OR "D-3"[tw] OR "D-5"[tw] OR D2[tw] OR D6[tw] OR "2 days"[tw] OR "6 days"[tw] OR "day 2"[tw] OR "day 6"[tw] OR "D-2"[tw] OR "D-6"[tw]
#15. #8 AND #14	291, PICO8,RCT

EMBASE

#1. 'in vitro fertilization'/exp OR (((artificial OR intrauterine OR intra-uterine OR oviduct* OR uterin*) NEAR/3 insemination*) OR (embryo NEAR/3 (disposition* OR Transfer*)) OR ("in vitro" NEAR/3 fertilization*) OR ((intracytoplasmic OR "intra-cytoplasmic" OR single) NEAR/3 "sperm injection*") OR (ovulation NEAR/3 (induction* OR Stimulation*)) OR IVF OR ICSI OR IUI OR "Test-Tube Fertilization*" OR "Test-Tube Babies" OR "Test-Tube Baby" OR "testtube baby" OR "testtube babies"):ab,ti,kw
#2. 'ganirelix'/exp OR 'cetrorelix'/exp OR 'iturelix'/exp OR ("Gonadotropin-releasing hormone antagonist*" OR "GnRH antagonist*" OR "GnRH-ant" OR "GnRH receptor antagonist*" OR GnRHant OR ganirelix OR "RS-26306" OR Antagon OR orgalutran OR cetrorelix OR "SB-75" OR Cetrotide OR iturelix OR Antide OR "ORF 23541"):ab,ti,kw	7553
#3. #1 and #2	4265
#4. 'meta analysis'/exp OR 'meta analysis (topic)'/exp OR 'systematic review'/exp OR 'systematic review (topic)'/exp OR (Systemat* NEAR/3 Review* OR 'Meta Analysis*' OR Metaanalys*):ab,ti,kw
#5. ('controlled clinical trial'/exp OR 'Controlled Clinical Trial (Topic)'/exp OR 'double blind procedure'/de OR 'control group'/de OR 'crossover procedure'/de OR 'single blind procedure'/de OR 'triple blind procedure'/de OR 'placebo'/de OR 'randomization'/exp OR (random* OR trial OR groups OR placebo* OR crossover OR "cross-over"):ab,ti,kw) AND 'human'/exp
#6. ('observational study'/exp OR 'cohort analysis'/exp OR 'cross-sectional study'/exp OR 'case control study'/exp OR ("observational stud*" OR Cohort OR "Follow-Up" OR Longitudinal* OR Prospectiv* OR Retrospectiv* OR "Case-Control" OR "Cross-Sectional" OR "case-series" OR "single arm"):ab,ti,kw) AND 'human'/exp
#7. #3 and #4	231 ，pico1-8，SR
#8. #3 and#5	2245，pico1-4，RCT
#9. #3 and #6	2189，pico1-8，OS
#10. ((GnRHa OR "GnRH agonist" OR "gonadotrophin-releasing hormone analogues" OR "gonadotrophin-releasing hormone agonist" OR "GnRH-a") NEAR/5 trigger*):ab,ti,kw
#11. #8 AND #10	250, PICO5/6, RCT
#12. ((fresh OR frozen) NEAR/3 embryo*):ab,ti,kw
#13. #8 AND #12	320, PICO7, RCT
#14. ("cleavage stage" OR "blastocyst stage" OR D3 OR D5 OR "3 days" OR "5 days" OR "day 3" OR "day 5" OR "D-3" OR "D-5" OR D2 OR D6 OR "2 days" OR "6 days" OR "day 2" OR "day 6" OR "D-2" OR "D-6"):ab,ti,kw
#15. #8 AND #14	661, PICO8,RCT

Web Of Science

#1. TS=(((artificial OR intrauterine OR intra-uterine OR oviduct* OR uterin*) NEAR/3 insemination*) OR (embryo NEAR/3 (disposition* OR Transfer*)) OR ("in vitro" NEAR/3 fertilization*) OR ((intracytoplasmic OR "intra-cytoplasmic" OR single) NEAR/3 "sperm injection*") OR (ovulation NEAR/3 (induction* OR Stimulation*)) OR IVF OR ICSI OR IUI OR "Test-Tube Fertilization*" OR "Test-Tube Babies" OR "Test-Tube Baby" OR "testtube baby" OR "testtube babies")
#2. TS=("Gonadotropin-releasing hormone antagonist*" OR "GnRH antagonist*" OR "GnRH-ant" OR "GnRH receptor antagonist*" OR GnRHant OR ganirelix OR "RS-26306" OR Antagon OR orgalutran OR cetrorelix OR "SB-75" OR Cetrotide OR iturelix OR Antide OR "ORF 23541")	4967
#3. #1 and #2	2174
#4. TS=("Meta-Analysis" OR "Meta Analysis" OR "Systematic Review" OR "Systematic Review*" OR "Meta-Analysis" OR "Meta-Analy*" OR "Metaanaly*" OR ("systematic*" NEAR/4 "Review*"))
#5. TS=("controlled trial" OR "Clinical Trial" OR (Random* NEAR/5 Trial) OR "Random Allocation" OR Randomization OR "Double Blind" OR "single blind" OR "Control Group" OR "Control Groups" OR "cross over" OR placebo* OR crossover OR random* OR trial OR groups) OR TI=("trial" OR "RCT" OR "groups" OR "group")
#6. TS=("case control" OR family OR longitudinal* OR retrospect* OR prospect* OR cohort OR "follow up" OR observational OR epidemiological OR "cross sectional" or "cross-sectional" OR "single arm")
#7. #3 and #4	205 ，pico1-8，SR
#8. #3 and#5	1316，pico1-4，RCT
#9. #3 and #6	944，pico1-8，OS
#10. TS=((GnRHa OR "GnRH agonist" OR "gonadotrophin-releasing hormone analogues" OR "gonadotrophin-releasing hormone agonist" OR "GnRH-a") NEAR/5 trigger*)
#11. #8 AND #10	136, PICO5/6, RCT
#12. TS=((fresh OR frozen) NEAR/3 embryo*)
#13. #8 AND #12	160, PICO7, RCT
#14. TS=("cleavage stage" OR "blastocyst stage" OR D3 OR D5 OR "3 days" OR "5 days" OR "day 3" OR "day 5" OR "D-3" OR "D-5" OR D2 OR D6 OR "2 days" OR "6 days" OR "day 2" OR "day 6" OR "D-2" OR "D-6")
#15. #8 AND #14	315, PICO8,RCT

Cochrane

#1	MeSH descriptor: [Fertilization in Vitro] explode all trees	2218

#2	MeSH descriptor: [Reproductive Techniques, Assisted] explode all trees	3428

#3	(((artificial OR intrauterine OR intra-uterine OR oviduct* OR uterin*) NEAR/3 insemination*) OR (embryo NEAR/3 (disposition* OR Transfer*)) OR ("in vitro" NEAR/3 fertilization*) OR ((intracytoplasmic OR "intra-cytoplasmic" OR single) NEAR/3 "sperm injection*") OR (ovulation NEAR/3 (induction* OR Stimulation*)) OR IVF OR ICSI OR IUI OR "Test-Tube Fertilization*" OR "Test-Tube Babies" OR "Test-Tube Baby" OR "testtube baby" OR "testtube babies"):ti,ab,kw	11992

#4	#1 or #2 or #3	12100

#5	"Gonadotropin-releasing hormone antagonist*" OR "GnRH antagonist*" OR "GnRH-ant" OR "GnRH receptor antagonist*" OR GnRHant OR ganirelix OR "RS-26306" OR Antagon OR orgalutran OR cetrorelix OR "SB-75" OR Cetrotide OR iturelix OR Antide OR "ORF 23541"	1702

#6	#4 and #5	1165，reviews 30，trials 1132，answers 3

#7	((GnRHa OR "GnRH agonist" OR "gonadotrophin-releasing hormone analogues" OR "gonadotrophin-releasing hormone agonist" OR "GnRH-a") NEAR/5 trigger*):ti,ab,kw	2275

#8	#6 AND #7	452，pico5/6 reviews 20，trials 432

#9	((fresh OR frozen) NEAR/3 embryo*):ti,ab,kw	1363

#10	#6 AND #9	135，pico7 reviews 2，trials 133

#11	"cleavage stage" OR "blastocyst stage" OR D3 OR D5 OR "3 days" OR "5 days" OR "day 3" OR "day 5" OR "D-3" OR "D-5" OR D2 OR D6 OR "2 days" OR "6 days" OR "day 2" OR "day 6" OR "D-2" OR "D-6"	92978

#12	#6 AND #11	438，pico8 reviews 20，trials 417，answers 1
