# Supplementary material for: Recommendations for the management of GnRH‐ant for ovarian stimulation in the assistant reproductive process: Benefits and harms for different ovarian response population
Source: Clin Public Health Guidel. 2025 May 21;2(3):e70022. doi: 10.1002/gin2.70022 (PMC13563367; doi:10.1002/gin2.70022)
Supplement: Supplementary file 4 [file GIN2-2-e70022-s005.docx]

Table of Content

[PICO 1 Whether the GnRH-ant protocol is recommended over the GnRH agonist (GnRH-a) protocols?	2](#_Toc27909)

[1.1 Normal Ovarian Responses	2](#_Toc12371)

[1.2 Poor Ovarian Responses	4](#_Toc30862)

[1.3 High Ovarian Responses	7](#_Toc4376)

[PICO 2 Among patients undergoing IVF assisted reproduction with COS using GnRH-ant, is it recommended to implement pretreatments?	10](#_Toc15538)

[2.1 Normal Ovarian Responses	10](#_Toc16238)

[2.2 Poor Ovarian Responses	20](#_Toc14094)

[2.3 High Ovarian Responses	26](#_Toc24420)

[PICO 3 Should fixed regimen or flexible regimen be preferred under GnRH-ant protocol?	28](#_Toc19637)

[PICO 4 Is early initiation of GnRH-ant during stimulation recommended?	30](#_Toc22007)

[4.1 Normal Ovarian Responses	30](#_Toc31230)

[4.2 Poor Ovarian Responses	32](#_Toc29745)

[4.3 High Ovarian Responses	34](#_Toc8626)

[PICO 5 Is the GnRHa add-on trigger recommended over the HCG trigger alone?	36](#_Toc6158)

[5.1 Add-on GnRHa trigger to HCG and HCG alone	36](#_Toc32462)

[5.2 GnRHa and HCG alone	39](#_Toc22979)

[PICO 6 For individuals undergoing fresh embryo transfer with GnRHa trigger, is reinforced luteal support recommended?	41](#_Toc5429)

[PICO 7 Fresh embryo transfer compared with frozen-all strategy for GnRH-ant protocol?	42](#_Toc20515)

[7.1 Normal Ovarian Responses	42](#_Toc8446)

[7.2 High Ovarian Responses	47](#_Toc3372)

[PICO 8 Which is the preferred timing to perform the embryo transfer: Day 3 or Day 5?	52](#_Toc16720)

# PICO 1 Whether the GnRH-ant protocol is recommended over the GnRH agonist (GnRH-a) protocols?

## 1.1 Normal Ovarian Responses

Question: Should GnRH-ant be used for IVF cycle patients compared to GnRH-a? (Normal Ovarian Responses )

| **Certainty assessment** | | | | | | | **№ of patients** | | **Effect** | | **Certainty** | **Importance** |
| --- | --- | --- | --- | --- | --- | --- | --- | --- | --- | --- | --- | --- |
| **№ of studies** | **Study design** | **Risk of bias** | **Inconsistency** | **Indirectness** | **Imprecision** | **Other considerations** | **GnRH-ant** | **GnRH-a** | **Relative (95% CI)** | **Absolute (95% CI)** |  |  |
| **Live birth rate (assessed with: Number of events )** | | | | | | | | | | | | |
| **2** | **randomised trials** | **not serious^a^** | **not serious** | **not serious** | **serious^b^** | **none** | **86/218 (39.4%)** | **92/218 (42.2%)** | **RR 0.93 (0.75 to 1.17)** | **30 fewer per 1,000 (from 106 fewer to 72 more)** | **⨁⨁⨁◯ Moderate** | **CRITICAL** |
| **Cumulative live birth rate** | | | | | | | | | | | | |
| **2** | **randomised trials** | **not serious^a^** | **serious^c^** | **not serious** | **serious^b^** | **none** | **377/807 (46.7%)** | **367/789 (46.5%)** | **RR 1.00 (0.86 to 1.17)** | **0 fewer per 1,000 (from 65 fewer to 79 more)** | **⨁⨁◯◯ Low** | **CRITICAL** |
| **Moderate and severe OHSS** | | | | | | | | | | | | |
| **7** | **randomised trials** | **not serious^a,d^** | **not serious** | **not serious** | **serious^b^** | **none** | **34/745 (4.6%)** | **45/553 (8.1%)** | **RR 0.65 (0.32 to 1.30)** | **28 fewer per 1,000 (from 55 fewer to 24 more)** | **⨁⨁⨁◯ Moderate** | **CRITICAL** |
| **Clinical pregnancy rates (assessed with: Number of events)** | | | | | | | | | | | | |
| **32** | **randomised trials** | **not serious^a,d^** | **not serious** | **not serious** | **not serious** | **none** | **1308/4191 (31.2%)** | **1226/3406 (36.0%)** | **RR 0.92 (0.87 to 0.97)** | **29 fewer per 1,000 (from 47 fewer to 11 fewer)** | **⨁⨁⨁⨁ High** | **CRITICAL** |
| **Implantation rate (assessed with: Number of events)** | | | | | | | | | | | | |
| **8** | **randomised trials** | **not serious^a,d^** | **not serious** | **not serious** | **not serious** | **none** | **263/1500 (17.5%)** | **332/1537 (21.6%)** | **RR 0.80 (0.66 to 0.95)** | **43 fewer per 1,000 (from 73 fewer to 11 fewer)** | **⨁⨁⨁⨁ High** | **CRITICAL** |
| **Metaphase II oocytes (assessed with: number of MII oocytes)** | | | | | | | | | | | | |
| **12** | **randomised trials** | **not serious^a,d^** | **very serious^e^** | **not serious** | **serious^b^** | **none** | **1035** | **1031** | **-** | **MD 0.77 fewer (1.61 fewer to 0.07 more)** | **⨁◯◯◯ Very low** | **IMPORTANT** |
| **Endometrial thickness (assessed with: mm)** | | | | | | | | | | | | |
| **11** | **randomised trials** | **not serious^a,d^** | **serious^f^** | **not serious** | **not serious** | **none** | **978** | **975** | **-** | **MD 0.35 lower (0.64 lower to 0.06 lower)** | **⨁⨁⨁◯ Moderate** | **IMPORTANT** |
| **Miscarriage rate (assessed with: number of events)** | | | | | | | | | | | | |
| **16** | **randomised trials** | **serious^g^** | **serious^h^** | **not serious** | **serious^i^** | **none** | **125/957 (13.1%)** | **106/754 (14.1%)** | **RR 0.96 (0.68 to 1.34)** | **6 fewer per 1,000 (from 45 fewer to 48 more)** | **⨁◯◯◯ Very low** | **NOT IMPORTANT** |

**CI: confidence interval; MD: mean difference; RR: risk ratio**

**Explanations**

a. Although included studies have a high risk of performance bias, the outcome is unlikely to be affected.

b. Downgraded by one level due to imprecision: the effect could be both beneficial and harmful.

c. Downgraded by one level due to inconsistency: the I^2^ value is high (I^2^=58%). We explored the clinical, methdological and statistical characteristics of the studies, but the unexplained heterogeneity remained. This potentially reduces our certainty on the observed magnitude of effect.

d. One of the included studies also has a high/unclear risk of selection bias, but the study is unlikely to affect the final estimates (weight <20%).

e. Downgraded by two levels due to inconsistency: the I^2^ value is high (I^2^ > 80%). We explored the clinical, methdological and statistical characteristics of the studies, but the unexplained heterogeneity remained. This potentially reduces our certainty on the observed magnitude of effect

f. Downgraded by one level due to inconsistency: the I^2^ value is high (I^2^=75%). We explored the clinical, methdological and statistical characteristics of the studies, but the unexplained heterogeneity remained. This potentially reduces our certainty on the observed magnitude of effect .

g. Downgraded by one level due to high/unclear risk of selection bias (weight>20%).

h. Downgraded by one level due to inconsistency: the I^2^<40%. We explored the clinical, methdological and statistical characteristics of the studies, but the unexplained heterogeneity remained. This potentially reduces our certainty on the observed magnitude of effect.

i. Downgraded by one level due to imprecision: low event rate and the effect could be both beneficial and harmful.

## 1.2 Poor Ovarian Responses

Should GnRH-ant be used for IVF cycle patients compared to GnRH-a? (Poor Ovarian Responses )

| **Certainty assessment** | | | | | | | **№ of patients** | | **Effect** | | **Certainty** | **Importance** |
| --- | --- | --- | --- | --- | --- | --- | --- | --- | --- | --- | --- | --- |
| **№ of studies** | **Study design** | **Risk of bias** | **Inconsistency** | **Indirectness** | **Imprecision** | **Other considerations** | **GnRH-ant** | **GnRH-a** | **Relative (95% CI)** | **Absolute (95% CI)** |  |  |
| **Live birth rate (assessed with: Number of events )** | | | | | | | | | | | | |
| **3^1,2^** | **randomised trials** | **serious^a^** | **not serious** | **not serious** | **serious^b^** | **none** | **20/196 (10.2%)** | **26/190 (13.7%)** | **RR 0.73 (0.43 to 1.24)** | **37 fewer per 1,000 (from 78 fewer to 33 more)** | **⨁⨁◯◯ Low** | **CRITICAL** |
| **Cumulative live birth rate** | | | | | | | | | | | | |
| **1^3^** | **randomised trials** | **not serious^c^** | **not serious** | **not serious** | **serious^b^** | **none** | **19/97 (19.6%)** | **17/94 (18.1%)** | **RR 1.08 (0.60 to 1.95)** | **14 more per 1,000 (from 72 fewer to 172 more)** | **⨁⨁⨁◯ Moderate** | **CRITICAL** |
| **Clinical pregnancy rates (assessed with: Number of events)** | | | | | | | | | | | | |
| **15** | **randomised trials** | **not serious^c^** | **not serious** | **not serious** | **serious^d^** | **none** | **201/1041 (19.3%)** | **208/998 (20.8%)** | **RR 0.96 (0.71 to 1.23)** | **8 fewer per 1,000 (from 60 fewer to 48 more)** | **⨁⨁⨁◯ Moderate** | **CRITICAL** |
| **Implantation rate (assessed with: Number of events)** | | | | | | | | | | | | |
| **7** | **randomised trials** | **not serious^c^** | **not serious** | **not serious** | **serious^d^** | **none** | **119/699 (17.0%)** | **138/782 (17.6%)** | **RR 0.98 (0.73 to 1.31)** | **4 fewer per 1,000 (from 48 fewer to 55 more)** | **⨁⨁⨁◯ Moderate** | **CRITICAL** |
| **Metaphase II oocytes (assessed with: number of MII oocytes)** | | | | | | | | | | | | |
| **7** | **randomised trials** | **serious^a^** | **very serious^e^** | **not serious** | **serious^d^** | **none** | **627** | **619** | **-** | **MD 0.2 more (0.21 fewer to 0.62 more)** | **⨁◯◯◯ Very low** | **IMPORTANT** |
| **Endometrial thickness (assessed with: mm)** | | | | | | | | | | | | |
| **11** | **randomised trials** | **serious^a^** | **very serious^f^** | **not serious** | **not serious** | **none** | **830** | **791** | **-** | **MD 0.71 fewer (1.34 fewer to 0.074 fewer)** | **⨁◯◯◯ Very low** | **IMPORTANT** |
| **Miscarriage rate (assessed with: number of events)** | | | | | | | | | | | | |
| **6** | **randomised trials** | **serious^a^** | **serious^g^** | **not serious** | **serious^b^** | **none** | **22/72 (30.6%)** | **14/69 (20.3%)** | **RR 1.22 (0.60 to 2.49)** | **45 more per 1,000 (from 81 fewer to 302 more)** | **⨁◯◯◯ Very low** | **NOT IMPORTANT** |

**CI:** confidence interval; **MD:** mean difference; **RR:** risk ratio

**Explanations**

a. Downgraded by one level due to high/unclear risk of selection bias (weight>20%)

b. Downgraded by one level due to imprecision: low event rate and the effect could be both beneficial and harmful.

c. Although included studies have high risk performance bias, it is unlikely to influence the final outcome.

d. Downgraded by one level due to imprecision: the effect could be both beneficial and harmful.

e. Downgraded by two levels due to inconsistency: the I^2^ value is high (I^2^ > 80%). We explored the clinical, methdological and statistical characteristics of the studies, but the unexplained heterogeneity remained. This potentially reduces our certainty on the observed magnitude of effect.

f. Downgraded by one level due to inconsistency: the I^2^ value is high (I^2^ > 80%). We explored the clinical, methdological and statistical characteristics of the studies, but the unexplained heterogeneity remained. This potentially reduces our certainty on the observed magnitude of effect.

g. Downgraded by one level due to inconsistency: the points estimates are in different directions across included studies (weight>20%). The I^2^ is low but we consider it because of the wide CI and small sample size.

## 1.3 High Ovarian Responses

Question: Should GnRH-ant be used for IVF cycle patients compared to GnRH-a? (High Ovarian Responses )

| **Certainty assessment** | | | | | | | **№ of patients** | | **Effect** | | **Certainty** | **Importance** |
| --- | --- | --- | --- | --- | --- | --- | --- | --- | --- | --- | --- | --- |
| **№ of studies** | **Study design** | **Risk of bias** | **Inconsistency** | **Indirectness** | **Imprecision** | **Other considerations** | **GnRH-ant** | **GnRH-a** | **Relative (95% CI)** | **Absolute (95% CI)** |  |  |
| **Live birth rate (assessed with: Number of events )** | | | | | | | | | | | | |
| **11** | **randomised trials** | **not serious** | **not serious** | **not serious** | **seriousa** | **none** | **36/105 (34.3%)** | **36/103 (35.0%)** | RR 0.98 **(0.68 to 1.43)** | **7 fewer per 1,000 (from 112 fewer to 150 more)** | **⨁⨁⨁◯ Moderate** | CRITICAL |
| Moderate and severe OHSS | | | | | | | | | | | | |
| **5** | **randomised trials** | **not seriousb** | **not serious** | **not serious** | **not serious** | **none** | **58/249 (23.3%)** | **121/273 (44.3%)** | **RR 0.48 (0.27 to 0.86)** | **230 fewer per 1,000 (from 324 fewer to 62 fewer)** | **⨁⨁⨁⨁ High** | CRITICAL |
| Clinical pregnancy rates (assessed with: Number of events) | | | | | | | | | | | | |
| **10** | **randomised trials** | **not serious** | **seriousc** | **not serious** | **seriousd** | **none** | **223/509 (43.8%)** | **232/532 (43.6%)** | **RR 1.01 (0.87 to 1.17)** | **4 more per 1,000 (from 57 fewer to 74 more)** | **⨁⨁◯◯ Low** | CRITICAL |
| Implantation rate (assessed with: Number of events) | | | | | | | | | | | | |
| **2** | **randomised trials** | **not serious** | **not serious** | **not serious** | **seriousd** | **none** | **47/191 (24.6%)** | **55/204 (27.0%)** | **RR 0.93 (0.66 to 1.29)** | **19 fewer per 1,000 (from 92 fewer to 78 more)** | **⨁⨁⨁◯ Moderate** | CRITICAL |
| Metaphase II oocytes (assessed with: number of MII oocytes) | | | | | | | | | | | | |
| **3** | **randomised trials** | **not serious** | **not serious** | **not serious** | **seriousd** | **none** | **228** | **232** | **-** | **MD** 0.72 more **(0.91 fewer to 2.36 more)** | **⨁⨁⨁◯ Moderate** | IMPORTANT |
| Endometrial thickness (assessed with: mm) | | | | | | | | | | | | |
| **5** | **randomised trials** | **not serious** | **seriouse** | **not serious** | **seriousd** | **none** | **362** | **361** | **-** | **MD** 0.57 lower **(1.19 lower to 0.05 higher)** | **⨁⨁◯◯ Low** | IMPORTANT |
| Miscarriage rate (assessed with: number of events) | | | | | | | | | | | | |
| **5** | **randomised trials** | **seriousf** | **seriousc** | **not serious** | **seriousd** | **none** | **33/227 (14.5%)** | **27/220 (12.3%)** | RR 1.02 **(0.64 to 1.63)** | **2 more per 1,000 (from 44 fewer to 77 more)** | **⨁◯◯◯ Very low** | NOT IMPORTANT |

**CI:** confidence interval; **MD:** mean difference; **RR:** risk ratio

**Explanations**

a. Downgraded by one level due to high/unclear risk of selection bias (weight>20%)

b. Downgraded by one level due to imprecision: low event rate and the effect could be both beneficial and harmful.

c. Although included studies have high risk performance bias, it is unlikely to influence the final outcome.

d. Downgraded by one level due to imprecision: the effect could be both beneficial and harmful.

e. Downgraded by two levels due to inconsistency: the I^2^ value is high (I^2^ > 80%). We explored the clinical, methdological and statistical characteristics of the studies, but the unexplained heterogeneity remained. This potentially reduces our certainty on the observed magnitude of effect.

f. Downgraded by one level due to inconsistency: the I^2^ value is high (I^2^ > 80%). We explored the clinical, methdological and statistical characteristics of the studies, but the unexplained heterogeneity remained. This potentially reduces our certainty on the observed magnitude of effect.

g. Downgraded by one level due to inconsistency:The I^2^ is low but we consider it because of the wide CI and small sample size.

# PICO 2 Among patients undergoing IVF assisted reproduction with COS using GnRH-ant, is it recommended to implement pretreatments?

## 2.1 Normal Ovarian Responses

2.1.1 Pretreatment with Oral Contraceptive Pill (OCP)

Question: OCP compared to Control for IVF assisted pregnancy patients (Normal Ovarian Responses)

| **Certainty assessment** | | | | | | | **№ of patients** | | **Effect** | | **Certainty** | **Importance** |
| --- | --- | --- | --- | --- | --- | --- | --- | --- | --- | --- | --- | --- |
| **№ of studies** | **Study design** | **Risk of bias** | **Inconsistency** | **Indirectness** | **Imprecision** | **Other considerations** | **OCP** | **Control** | **Relative (95% CI)** | **Absolute (95% CI)** |  |  |
| **Live birth rate - NOR** | | | | | | | | | | | | |
| **1** | **randomised trials** | **not serious^a^** | **not serious** | **not serious** | **very serious^b^** | **none** | **3/20 (15.0%)** | **7/24 (29.2%)** | **RR 0.51 (0.15 to 1.73)** | **143 fewer per 1,000 (from 248 fewer to 213 more)** | **⨁⨁◯◯ Low** | **CRITICAL** |
| **Cumulative live birth rate - NOR** | | | | | | | | | | | | |
| **1** | **randomised trials** | **not serious^c^** | **not serious** | **not serious** | **very serious^b^** | **none** | **12/31 (38.7%)** | **10/21 (47.6%)** | **RR 0.81 (0.43 to 1.53)** | **90 fewer per 1,000 (from 271 fewer to 252 more)** | **⨁⨁◯◯ Low** | **CRITICAL** |
| **Endometrial thickness (mm) - NOR** | | | | | | | | | | | | |
| **2** | **randomised trials** | **not serious^a,c^** | **not serious** | **not serious** | **serious^d^** | **none** | **267** | **281** | **-** | **MD 0.21 lower (0.53 lower to 0.12 higher)** | **⨁⨁⨁◯ Moderate** | **IMPORTANT** |
| **Metaphase II oocytes- NOR** | | | | | | | | | | | | |
| **2** | **randomised trials** | **not serious^c^** | **not serious** | **not serious** | **serious^e^** | **none** | **142** | **142** | **-** | **MD 1.56 higher (0.14 higher to 2.99 higher)** | **⨁⨁⨁◯ Moderate** | **IMPORTANT** |
| **Clinical pregnancy rate - NOR** | | | | | | | | | | | | |
| **5** | **randomised trials** | **not serious^a^** | **not serious** | **not serious** | **very serious^b^** | **none** | **111/344 (32.3%)** | **147/346 (42.5%)** | **RR 0.77 (0.55 to 1.07)** | **98 fewer per 1,000 (from 191 fewer to 30 more)** | **⨁⨁◯◯ Low** | **IMPORTANT** |
| **Moderate risk of OHSS- NOR** | | | | | | | | | | | | |
| **2** | **randomised trials** | **not serious** | **serious^f^** | **not serious** | **very serious^b^** | **none** | **3/319 (0.9%)** | **4/310 (1.3%)** | **RR 0.77 (0.13 to 4.70)** | **3 fewer per 1,000 (from 11 fewer to 48 more)** | **⨁◯◯◯ Very low** | **IMPORTANT** |
| **Severe risk of OHSS- NOR** | | | | | | | | | | | | |
| **2** | **randomised trials** | **not serious** | **not serious** | **not serious** | **very serious^b^** | **none** | **1/319 (0.3%)** | **0/310 (0.0%)** | **RR 2.86 (0.12 to 69.73)** | **0 fewer per 1,000 (from 0 fewer to 0 fewer)** | **⨁⨁◯◯ Low** | **IMPORTANT** |
| **Miscarriage rate - NOR** | | | | | | | | | | | | |
| **4** | **randomised trials** | **serious^g^** | **serious^f^** | **not serious** | **serious^d^** | **none** | **39/188 (20.7%)** | **28/203 (13.8%)** | **RR 1.37 (0.84 to 2.25)** | **51 more per 1,000 (from 22 fewer to 172 more)** | **⨁◯◯◯ Very low** | **NOT IMPORTANT** |

**CI: confidence interval; MD: mean difference; RR: risk ratio**

**Explanations**

a. Although included studies have a high risk of performance bias, the outcome is unlikely to be affected.

b. Downgraded by two levels due to imprecision: low sample size and the effect could be both beneficial and harmful.

c. Included studies have the potential risk of selection bias (weight >20%). However, it did not affect the overall estimates.

d. Downgraded by one level due to imprecision: the effect could be both beneficial and harmful.

e. Downgraded by one level due to imprecision: low sample size.

f. Downgraded by one level due to inconsistency: I^2^ is low but the point estimates are in different directions across included studies.

g. Included studies have the potential risk of selection bias (weight >20%).

2.1.2 Pretreatment with Estrogen

Question: Estrogen compared to Control for IVF assisted pregnancy patients (Normal Ovarian Responses)

| **Certainty assessment** | | | | | | | **№ of patients** | | **Effect** | | **Certainty** | **Importance** |
| --- | --- | --- | --- | --- | --- | --- | --- | --- | --- | --- | --- | --- |
| **№ of studies** | **Study design** | **Risk of bias** | **Inconsistency** | **Indirectness** | **Imprecision** | **Other considerations** | **Estrogen** | **Control** | **Relative (95% CI)** | **Absolute (95% CI)** |  |  |
| **Live birth rate - NOR** | | | | | | | | | | | | |
| **1** | **randomised trials** | **not serious^a^** | **not serious** | **not serious** | **very serious^b^** | **none** | **3/19 (15.8%)** | **7/24 (29.2%)** | **RR 0.54 (0.16 to 1.82)** | **134 fewer per 1,000 (from 245 fewer to 239 more)** | **⨁⨁◯◯ Low** | **CRITICAL** |
| **Cumulative live birth rate** | | | | | | | | | | | | |
| **1** | **randomised trials** | **not serious** | **not serious** | **not serious** | **very serious^b^** | **none** | **6/22 (27.3%)** | **10/21 (47.6%)** | **RR 0.57 (0.25 to 1.30)** | **205 fewer per 1,000 (from 357 fewer to 143 more)** | **⨁⨁◯◯ Low** |  |
| **Implantation rate-NOR** | | | | | | | | | | | | |
| **1** | **randomised trials** | **not serious** | **not serious** | **not serious** | **very serious^b^** | **none** | **89/337 (26.4%)** | **95/323 (29.4%)** | **RR 0.90 (0.70 to 1.15)** | **29 fewer per 1,000 (from 88 fewer to 44 more)** | **⨁⨁◯◯ Low** | **CRITICAL** |
| **Endometrial thickness (mm) - NOR** | | | | | | | | | | | | |
| **2** | **randomised trials** | **not serious** | **not serious** | **not serious** | **serious^c^** | **none** | **296** | **290** | **-** | **MD 0.01 higher (0.3 lower to 0.31 higher)** | **⨁⨁⨁◯ Moderate** | **IMPORTANT** |
| **Metaphase II oocytes-Start stimulation - NOR** | | | | | | | | | | | | |
| **3** | **randomised trials** | **not serious^a^** | **not serious** | **not serious** | **serious^c^** | **none** | **300** | **288** | **-** | **MD 0.53 higher (0.23 lower to 1.29 higher)** | **⨁⨁⨁◯ Moderate** | **IMPORTANT** |
| **Clinical pregnancy rate - NOR** | | | | | | | | | | | | |
| **7** | **randomised trials** | **not serious^a^** | **serious^d^** | **not serious** | **very serious^b^** | **none** | **88/245 (35.9%)** | **95/249 (38.2%)** | **RR 0.91 (0.68 to 1.23)** | **34 fewer per 1,000 (from 122 fewer to 88 more)** | **⨁◯◯◯ Very low** | **IMPORTANT** |
| **Miscarriage rate - NOR** | | | | | | | | | | | | |
| **3** | **randomised trials** | **not serious^a^** | **serious^d^** | **not serious** | **very serious^b^** | **none** | **17/257 (6.6%)** | **17/249 (6.8%)** | **RR 1.02 (0.53 to 1.99)** | **1 more per 1,000 (from 32 fewer to 68 more)** | **⨁◯◯◯ Very low** | **NOT IMPORTANT** |

**CI: confidence interval; MD: mean difference; RR: risk ratio**

**Explanations**

a. Although included studies have a high risk of performance bias, the outcome is unlikely to be affected.

b. Downgraded by two levels due to imprecision: low event rate and effect could be both beneficial or harmful.

c. Downgraded by one level due to imprecision: wide confidence interval.

d. Downgraded by one level due to inconsistency: I^2^ is low and we consider it is because of the wide CI and small sample size. The point estimates are in different directions across included studies.

2.1.3 Pretreatment with GnRH-ant

Question: GnRH-ant compared to Control for IVF assisted pregnancy patients (Normal Ovarian Responses)

| **Certainty assessment** | | | | | | | **№ of patients** | | **Effect** | | **Certainty** | **Importance** |
| --- | --- | --- | --- | --- | --- | --- | --- | --- | --- | --- | --- | --- |
| **№ of studies** | **Study design** | **Risk of bias** | **Inconsistency** | **Indirectness** | **Imprecision** | **Other considerations** | **GnRH-ant** | **Control** | **Relative (95% CI)** | **Absolute (95% CI)** |  |  |
| **Live birth rate-NOR** | | | | | | | | | | | | |
| **1** | **randomised trials** | **not serious^a^** | **not serious** | **not serious** | **very serious^b^** | **none** | **20/59 (33.9%)** | **25/58 (43.1%)** | **RR 0.79 (0.49 to 1.25)** | **91 fewer per 1,000 (from 220 fewer to 108 more)** | **⨁⨁◯◯ Low** | **CRITICAL** |
| **Implantation rate-NOR** | | | | | | | | | | | | |
| **2** | **randomised trials** | **not serious^a^** | **serious^c^** | **not serious** | **very serious^b^** | **none** | **54/145 (37.2%)** | **54/144 (37.5%)** | **RR 1.01 (0.75 to 1.36)** | **4 more per 1,000 (from 94 fewer to 135 more)** | **⨁◯◯◯ Very low** | **CRITICAL** |
| **Clinical pregnancy rate - NOR** | | | | | | | | | | | | |
| **2** | **randomised trials** | **not serious^a^** | **serious^c^** | **not serious** | **very serious^b^** | **none** | **34/79 (43.0%)** | **37/78 (47.4%)** | **RR 0.90 (0.64 to 1.26)** | **47 fewer per 1,000 (from 171 fewer to 123 more)** | **⨁◯◯◯ Very low** | **IMPORTANT** |
| **Metaphase II oocytes-NOR** | | | | | | | | | | | | |
| **1** | **randomised trials** | **not serious** | **not serious** | **not serious** | **serious^d^** | **none** | **277/343 (80.8%)** | **223/277 (80.5%)** | **RR 1.00 (0.93 to 1.08)** | **0 fewer per 1,000 (from 56 fewer to 64 more)** | **⨁⨁⨁◯ Moderate** | **IMPORTANT** |
| **Metaphase II oocytes-Random - NOR** | | | | | | | | | | | | |
| **1** | **randomised trials** | **not serious^a^** | **not serious** | **not serious** | **serious^e^** | **none** | **68** | **68** | **-** | **MD 2 lower (3.47 lower to 0.53 lower)** | **⨁⨁⨁◯ Moderate** | **IMPORTANT** |
| **Moderate, severe risk of OHSS-Random - NOR** | | | | | | | | | | | | |
| **1** | **randomised trials** | **not serious^a^** | **not serious** | **not serious** | **very serious^b^** | **none** | **1/68 (1.5%)** | **2/68 (2.9%)** | **RR 0.50 (0.05 to 5.39)** | **15 fewer per 1,000 (from 28 fewer to 129 more)** | **⨁⨁◯◯ Low** | **IMPORTANT** |
| **Miscarriage rate - NOR** | | | | | | | | | | | | |
| **2** | **randomised trials** | **not serious^a^** | **not serious** | **not serious** | **very serious^b^** | **none** | **6/44 (13.6%)** | **5/47 (10.6%)** | **RR 1.34 (0.44 to 4.03)** | **36 more per 1,000 (from 60 fewer to 322 more)** | **⨁⨁◯◯ Low** | **NOT IMPORTANT** |
| **Ectopic pregnancy rate-NOR** | | | | | | | | | | | | |
| **1** | **randomised trials** | **not serious^a^** | **not serious** | **not serious** | **very serious^b^** | **none** | **2/27 (7.4%)** | **2/31 (6.5%)** | **RR 1.15 (0.17 to 7.61)** | **10 more per 1,000 (from 54 fewer to 426 more)** | **⨁⨁◯◯ Low** | **NOT IMPORTANT** |

**CI: confidence interval; MD: mean difference; RR: risk ratio**

**Explanations**

a. Although included studies have a high risk of performance bias, the outcome is unlikely to be affected.

b. Downgraded by two levels due to imprecision: low event rate and effect could be both beneficial or harmful.

c. Downgraded by one level due to inconsistency: I^2^ is low and we consider it is because of the wide CI and small sample size. The point estimates are in different directions across included studies (weight>20%).

d. Downgraded by one level due to imprecision: effect could be both beneficial or harmful.

e. Downgraded by one level due to imprecision: low sample size.

2.1.4 Pretreatment with Testosterone

Question: Testosterone compared to Control for IVF assisted pregnancy patients (Normal Ovarian Responses)

| **Certainty assessment** | | | | | | | **№ of patients** | | **Effect** | | **Certainty** | **Importance** |
| --- | --- | --- | --- | --- | --- | --- | --- | --- | --- | --- | --- | --- |
| **№ of studies** | **Study design** | **Risk of bias** | **Inconsistency** | **Indirectness** | **Imprecision** | **Other considerations** | **Testosterone** | **Control** | **Relative (95% CI)** | **Absolute (95% CI)** |  |  |
| **Live birth rate-Random - NOR** | | | | | | | | | | | | |
| **1** | **randomised trials** | **not serious** | **not serious** | **not serious** | **very serious^a^** | **none** | **14/53 (26.4%)** | **17/50 (34.0%)** | **RR 0.78 (0.43 to 1.41)** | **75 fewer per 1,000 (from 194 fewer to 139 more)** | **⨁⨁◯◯ Low** | **CRITICAL** |
| **Implantation rate-NOR** | | | | | | | | | | | | |
| **1** | **randomised trials** | **not serious** | **not serious** | **not serious** | **serious^b^** | **none** | **20/74 (27.0%)** | **21/61 (34.4%)** | **RR 0.79 (0.47 to 1.31)** | **72 fewer per 1,000 (from 182 fewer to 107 more)** | **⨁⨁⨁◯ Moderate** | **CRITICAL** |
| **Endometrial thickness on hCG day(mm)-Random - NOR** | | | | | | | | | | | | |
| **1** | **randomised trials** | **not serious** | **not serious** | **not serious** | **serious^c^** | **none** | **53** | **50** | **-** | **MD 1 higher (0.14 higher to 1.86 higher)** | **⨁⨁⨁◯ Moderate** | **IMPORTANT** |

**CI: confidence interval; MD: mean difference; RR: risk ratio**

**Explanations**

a. Downgraded by two levels due to imprecision: low sample size and the effect could be both beneficial and harmful.

b. Downgraded by one level due to imprecision: the effect could be both beneficial and harmful.

c. Downgraded by one level due to imprecision: low sample size.

2.1.5 Pretreatment with Progestogen

Question: Progestogen compared to Control for IVF assisted pregnancy patients (Normal Ovarian Responses)

| **Certainty assessment** | | | | | | | **№ of patients** | | **Effect** | | **Certainty** | **Importance** |
| --- | --- | --- | --- | --- | --- | --- | --- | --- | --- | --- | --- | --- |
| **№ of studies** | **Study design** | **Risk of bias** | **Inconsistency** | **Indirectness** | **Imprecision** | **Other considerations** | **Progestogen** | **Control** | **Relative (95% CI)** | **Absolute (95% CI)** |  |  |
| **Live birth rate-NOR** | | | | | | | | | | | | |
| **1** | **randomised trials** | **not serious^a^** | **not serious** | **not serious** | **very serious^b^** | **none** | **5/20 (25.0%)** | **7/24 (29.2%)** | **RR 0.86 (0.32 to 2.29)** | **41 fewer per 1,000 (from 198 fewer to 376 more)** | **⨁⨁◯◯ Low** | **CRITICAL** |
| **Oocytes retrieved-NOR** | | | | | | | | | | | | |
| **1** | **randomised trials** | **not serious^a^** | **not serious** | **not serious** | **very serious^b^** | **none** | **23** | **24** | **-** | **MD 2.7 higher (0.98 lower to 6.38 higher)** | **⨁⨁◯◯ Low** | **IMPORTANT** |
| **Pregnancy rate-NOR** | | | | | | | | | | | | |
| **1** | **randomised trials** | **not serious^a^** | **not serious** | **not serious** | **very serious^b^** | **none** | **7/20 (35.0%)** | **12/24 (50.0%)** | **RR 0.70 (0.34 to 1.44)** | **150 fewer per 1,000 (from 330 fewer to 220 more)** | **⨁⨁◯◯ Low** | **IMPORTANT** |

**CI: confidence interval; MD: mean difference; RR: risk ratio**

**Explanations**

a. Although included studies have a high risk of performance bias, the outcome is unlikely to be affected.

b. Downgraded by two levels due to imprecision: low sample size and event rate, and the effect could be both beneficial and harmful.

## 2.2 Poor Ovarian Responses

2.2.1 Pretreatment with Oral Contraceptive Pill (OCP)

Question: OCP compared to Control for IVF assisted pregnancy patients (Poor Ovarian Responses)

| **Certainty assessment** | | | | | | | **№ of patients** | | **Effect** | | **Certainty** | **Importance** |
| --- | --- | --- | --- | --- | --- | --- | --- | --- | --- | --- | --- | --- |
| **№ of studies** | **Study design** | **Risk of bias** | **Inconsistency** | **Indirectness** | **Imprecision** | **Other considerations** | **OCP** | **Control** | **Relative (95% CI)** | **Absolute (95% CI)** |  |  |
| **Live birth rate - POR** | | | | | | | | | | | | |
| **2** | **randomised trials** | **not serious^a^** | **not serious** | **not serious** | **very serious^b^** | **none** | **20/67 (29.9%)** | **13/67 (19.4%)** | **RR 1.54 (0.84 to 2.83)** | **105 more per 1,000 (from 31 fewer to 355 more)** | **⨁⨁◯◯ Low** | **CRITICAL** |
| **Implantation rate, % - POR** | | | | | | | | | | | | |
| **2** | **randomised trials** | **not serious^a^** | **not serious** | **not serious** | **very serious^b^** | **none** | **29/200 (14.5%)** | **18/195 (9.2%)** | **RR 1.57 (0.90 to 2.74)** | **53 more per 1,000 (from 9 fewer to 161 more)** | **⨁⨁◯◯ Low** | **CRITICAL** |
| **Endometrial thickness (mm) - POR** | | | | | | | | | | | | |
| **2** | **randomised trials** | **not serious^a^** | **not serious** | **not serious** | **serious^c^** | **none** | **67** | **67** | **-** | **MD 0.27 lower (0.88 lower to 0.35 higher)** | **⨁⨁⨁◯ Moderate** | **IMPORTANT** |
| **Clinical pregnancy rate - POR** | | | | | | | | | | | | |
| **2** | **randomised trials** | **not serious^a^** | **not serious** | **not serious** | **very serious^b^** | **none** | **23/67 (34.3%)** | **15/67 (22.4%)** | **RR 1.53 (0.88 to 2.67)** | **119 more per 1,000 (from 27 fewer to 374 more)** | **⨁⨁◯◯ Low** | **IMPORTANT** |
| **Miscarriage rate - POR** | | | | | | | | | | | | |
| **2** | **randomised trials** | **not serious^a^** | **not serious** | **not serious** | **very serious^b^** | **none** | **3/23 (13.0%)** | **2/15 (13.3%)** | **RR 0.97 (0.18 to 5.26)** | **4 fewer per 1,000 (from 109 fewer to 568 more)** | **⨁⨁◯◯ Low** | **NOT IMPORTANT** |

**CI: confidence interval; MD: mean difference; RR: risk ratio**

**Explanations**

a. One study has a potential risk of selection bias. However, the effect is unlikely to be affected.

b. Downgraded by two levels due to imprecision: low event rate and effect could be both beneficial or harmful.

c. Downgraded by one level due to imprecision: effect could be both beneficial or harmful.

2.2.2 Pretreatment with GnRH-ant

Question: GnRH-ant compared to Control for IVF assisted pregnancy patients (Poor Ovarian Responses)

| **Certainty assessment** | | | | | | | **№ of patients** | | **Effect** | | **Certainty** | **Importance** |
| --- | --- | --- | --- | --- | --- | --- | --- | --- | --- | --- | --- | --- |
| **№ of studies** | **Study design** | **Risk of bias** | **Inconsistency** | **Indirectness** | **Imprecision** | **Other considerations** | **GnRH-ant** | **Control** | **Relative (95% CI)** | **Absolute (95% CI)** |  |  |
| **Implantaion rate, %-POR** | | | | | | | | | | | | |
| **2** | **randomised trials** | **not serious** | **not serious** | **not serious** | **very serious^a^** | **none** | **6/49 (12.2%)** | **3/51 (5.9%)** | **RR 2.06 (0.54 to 7.83)** | **62 more per 1,000 (from 27 fewer to 402 more)** | **⨁⨁◯◯ Low** | **CRITICAL** |
| **Endometrial thickness at trigger (mm)-Random - POR** | | | | | | | | | | | | |
| **4** | **randomised trials** | **not serious** | **not serious** | **not serious** | **serious^b^** | **none** | **189** | **189** | **-** | **MD 0.82 higher (0.5 higher to 1.14 higher)** | **⨁⨁⨁◯ Moderate** | **IMPORTANT** |
| **Metaphase II oocytes-Random - POR** | | | | | | | | | | | | |
| **4** | **randomised trials** | **not serious** | **serious^c^** | **not serious** | **serious^d^** | **none** | **189** | **189** | **-** | **MD 0.65 higher (0.46 lower to 1.75 higher)** | **⨁⨁◯◯ Low** | **IMPORTANT** |
| **Clinical pregnancy rate - POR** | | | | | | | | | | | | |
| **3** | **randomised trials** | **not serious** | **not serious** | **not serious** | **serious^b^** | **none** | **32/156 (20.5%)** | **11/138 (8.0%)** | **RR 2.68 (1.41 to 5.09)** | **134 more per 1,000 (from 33 more to 326 more)** | **⨁⨁⨁◯ Moderate** | **IMPORTANT** |
| **Miscarriage rate - POR** | | | | | | | | | | | | |
| **3** | **randomised trials** | **not serious** | **not serious** | **not serious** | **very serious^e^** | **none** | **6/88 (6.8%)** | **5/83 (6.0%)** | **RR 0.9 (0.3 to 2.7)** | **6 fewer per 1,000 (from 42 fewer to 102 more)** | **⨁⨁◯◯ Low** | **NOT IMPORTANT** |

**CI: confidence interval; MD: mean difference; RR: risk ratio**

**Explanations**

a. Downgraded by two levels due to imprecision: low sample size and the effect could be both beneficial and harmful.

b. Downgraded by one level due to imprecision: low sample size.

c. Downgraded by one level due to inconsistency: the I^2^ value is high (I^2^ > 80%). We explored the clinical, methdological and statistical characteristics of the studies, but the unexplained heterogeneity remained. This potentially reduces our certainty on the observed magnitude of effect.

d. Downgraded by one level due to imprecision: the effect could be both beneficial and harmful.

e. Downgraded by two levels due to imprecision: low sample size, event rate, and the effect could be both beneficial and harmful.

2.2.3 Pretreatment with Testosterone

Question: Testosterone compared to Control for IVF assisted pregnancy patients (Poor Ovarian Responses)

| **Certainty assessment** | | | | | | | **№ of patients** | | **Effect** | | **Certainty** | **Importance** |
| --- | --- | --- | --- | --- | --- | --- | --- | --- | --- | --- | --- | --- |
| **№ of studies** | **Study design** | **Risk of bias** | **Inconsistency** | **Indirectness** | **Imprecision** | **Other considerations** | **Testosterone** | **Control** | **Relative (95% CI)** | **Absolute (95% CI)** |  |  |
| **Live birth rate-Random - POR** | | | | | | | | | | | | |
| **1** | **randomised trials** | **not serious^a^** | **not serious** | **not serious** | **very serious^b^** | **none** | **15/55 (27.3%)** | **7/55 (12.7%)** | **RR 2.14 (0.95 to 4.84)** | **145 more per 1,000 (from 6 fewer to 489 more)** | **⨁⨁◯◯ Low** | **CRITICAL** |
| **Implantation rate-POR** | | | | | | | | | | | | |
| **1** | **randomised trials** | **not serious^a^** | **not serious** | **not serious** | **serious^c^** | **none** | **20/140 (14.3%)** | **8/138 (5.8%)** | **RR 2.46 (1.12 to 5.41)** | **85 more per 1,000 (from 7 more to 256 more)^d^** | **⨁⨁⨁◯ Moderate** | **CRITICAL** |
| **Endometrial thickness on hCG day(mm)-Random - POR** | | | | | | | | | | | | |
| **1** | **randomised trials** | **not serious^a^** | **not serious** | **not serious** | **very serious^b^** | **none** | **55** | **55** | **-** | **MD 0.1 lower (0.59 lower to 0.39 higher)** | **⨁⨁◯◯ Low** | **IMPORTANT** |
| **Clinical pregnancy rate - POR** | | | | | | | | | | | | |
| 3 | randomised trials | serious^e^ | not serious | not serious | not serious | none | 53/164 (32.3%) | 23/163 (14.1%) | **RR 2.26** (1.46 to 3.51) | **178 more per 1,000** (from 65 more to 354 more) | ⨁⨁⨁◯ Moderate | IMPORTANT |
| **Metaphase II oocytes-Random - POR** | | | | | | | | | | | | |
| **1** | **randomised trials** | **not serious** | **not serious** | **not serious** | **very serious^b^** | **none** | **70** | **70** | **-** | **MD 0.8 higher (0.04 lower to 1.64 higher)** | **⨁⨁◯◯ Low** | **IMPORTANT** |
| **Miscarriage rate-POR** | | | | | | | | | | | | |
| **1** | **randomised trials** | **not serious^a^** | **not serious** | **not serious** | **very serious^b^** | **none** | **2/17 (11.8%)** | **1/8 (12.5%)** | **RR 0.94 (0.10 to 8.92)** | **8 fewer per 1,000 (from 113 fewer to 990 more)** | **⨁⨁◯◯ Low** | **NOT IMPORTANT** |

**CI: confidence interval; MD: mean difference; RR: risk ratio**

**Explanations**

a. Although included studies have a high risk of performance bias, the outcome is unlikely to be affected.

b. Downgraded by two levels due to imprecision: low sample size and the effect could be both beneficial and harmful.

c. Downgraded by one level due to imprecision: low event rate.

d. Included study also evaluated the mean difference for this outcome, the absolute effect is MD 0 (0.07 lower to 0.07 higher).

e. Downgraded by one level due to high risk of attrition bias.

## 2.3 High Ovarian Responses

2.3.1 Pretreatment with GnRH-ant

Question: Should GnRH-ant be used for IVF cycle patients compared to GnRH-a? (High Ovarian Responses )

| **Certainty assessment** | | | | | | | **№ of patients** | | **Effect** | | **Certainty** | **Importance** |
| --- | --- | --- | --- | --- | --- | --- | --- | --- | --- | --- | --- | --- |
| **№ of studies** | **Study design** | **Risk of bias** | **Inconsistency** | **Indirectness** | **Imprecision** | **Other considerations** | **GnRH-ant** | **Control** | **Relative (95% CI)** | **Absolute (95% CI)** |  |  |
| **Implantaion rate, %- HOR, favor GnRH-ant** | | | | | | | | | | | | |
| **1** | **randomised trials** | **not serious^a^** | **not serious** | **not serious** | **very serious^b^** | **none** | **38** | **50** | **-** | **MD 8.7 higher (1.69 lower to 19.09 higher)** | **⨁⨁◯◯ Low** | **CRITICAL** |
| **Metaphase II oocytes-Random - HOR, favor GnRH-ant** | | | | | | | | | | | | |
| **1** | **randomised trials** | **not serious^a^** | **not serious** | **not serious** | **very serious^b^** | **none** | **38** | **50** | **-** | **MD 0.55 higher (3.04 lower to 4.14 higher)** | **⨁⨁◯◯ Low** | **IMPORTANT** |
| **Clinical pregnancy rate - HOR, favor GnRH-ant** | | | | | | | | | | | | |
| **1** | **randomised trials** | **not serious^a^** | **not serious** | **not serious** | **very serious^b^** | **none** | **7/38 (18.4%)** | **2/50 (4.0%)** | **RR 4.61 (1.01 to 20.93)** | **144 more per 1,000 (from 0 fewer to 797 more)** | **⨁⨁◯◯ Low** | **IMPORTANT** |
| **Moderate, severe risk of OHSS-Random - HOR, favor control** | | | | | | | | | | | | |
| **1** | **randomised trials** | **not serious^a^** | **not serious** | **not serious** | **very serious^c^** | **none** | **15/38 (39.5%)** | **18/50 (36.0%)** | **RR 1.10 (0.64 to 1.88)** | **36 more per 1,000 (from 130 fewer to 317 more)** | **⨁⨁◯◯ Low** | **IMPORTANT** |
| **Miscarriage rate - HOR, favor GnRH-ant** | | | | | | | | | | | | |
| **1** | **randomised trials** | **not serious^a^** | **not serious** | **not serious** | **very serious^c^** | **none** | **3/7 (42.9%)** | **1/2 (50.0%)** | **RR 0.86 (0.17 to 4.37)** | **70 fewer per 1,000 (from 415 fewer to 1,000 more)** | **⨁⨁◯◯ Low** | **NOT IMPORTANT** |

**CI: confidence interval; MD: mean difference; RR: risk ratio**

**Explanations**

a. Included studies have an unclear risk of bias (weight >20%). However, it is unlikely to affect the overall estimates.

b. Downgraded by two levels due to imprecision: low event rate and effect could be both beneficial or harmful.

c. Downgraded by two levels due to imprecision: low sample size, low event rate and effect could be both beneficial and harmful.

# PICO 3 Should fixed regimen or flexible regimen be preferred under GnRH-ant protocol?

Question: Fixed compared to Flexible for IVF assisted pregnancy patients(Normal Ovarian Responses)

| **Certainty assessment** | | | | | | | **№ of patients** | | **Effect** | | **Certainty** | **Importance** |
| --- | --- | --- | --- | --- | --- | --- | --- | --- | --- | --- | --- | --- |
| **№ of studies** | **Study design** | **Risk of bias** | **Inconsistency** | **Indirectness** | **Imprecision** | **Other considerations** | **Fixed** | **Flexible** | **Relative (95% CI)** | **Absolute (95% CI)** |  |  |
| **Cumulation Live birth rate-NOR, favor fixed** | | | | | | | | | | | | |
| **2** | **randomised trials** | **serious^a^** | **not serious** | **not serious** | **serious^b^** | **none** | **160/409 (39.1%)** | **151/409 (36.9%)** | **RR 1.04 (0.89 to 1.21)** | **15 more per 1,000 (from 41 fewer to 78 more)** | **⨁⨁◯◯ Low** | **CRITICAL** |
| **Moderate OHSS-NOR, favor flexible** | | | | | | | | | | | | |
| **1** | **randomised trials** | **not serious^c^** | **not serious** | **not serious** | **very serious^d^** | **none** | **5/100 (5.0%)** | **4/101 (4.0%)** | **RR 1.26 (0.35 to 4.57)** | **10 more per 1,000 (from 26 fewer to 141 more)** | **⨁⨁◯◯ Low** | **CRITICAL** |
| **Implantation rate-NOR, favor fixed** | | | | | | | | | | | | |
| **1** | **randomised trials** | **not serious** | **not serious** | **not serious** | **very serious^d^** | **none** | **30/91 (33.0%)** | **30/94 (31.9%)** | **RR 1.03 (0.68 to 1.57)** | **10 more per 1,000 (from 102 fewer to 182 more)^e^** | **⨁⨁◯◯ Low** | **CRITICAL** |
| **Clinical pregnancy rate-NOR, favor fixed** | | | | | | | | | | | | |
| **4** | **randomised trials** | **not serious^c^** | **not serious** | **not serious** | **serious^b^** | **none** | **76/239 (31.8%)** | **64/237 (27.0%)** | **RR 1.16 (0.89 to 1.51)** | **43 more per 1,000 (from 30 fewer to 138 more)** | **⨁⨁⨁◯ Moderate** | **IMPORTANT** |
| **Metaphase II oocytes-NOR, favor flexible** | | | | | | | | | | | | |
| **3** | **randomised trials** | **not serious^c^** | **not serious** | **not serious** | **serious^b^** | **none** | **223** | **231** | **-** | **MD 0.08 lower (0.77 lower to 0.6 higher)** | **⨁⨁⨁◯ Moderate** | **IMPORTANT** |
| **Endometrial thickness on the day of ET, mm-NOR, favor flexible** | | | | | | | | | | | | |
| **1** | **randomised trials** | **not serious^c^** | **not serious** | **not serious** | **very serious^f^** | **none** | **46** | **47** | **-** | **MD 0.3 lower (0.85 lower to 0.25 higher)** | **⨁⨁◯◯ Low** | **IMPORTANT** |
| **Early pregnancy loss-NOR, favor fixed** | | | | | | | | | | | | |
| **2** | **randomised trials** | **serious^a^** | **not serious** | **not serious** | **very serious^d^** | **none** | **2/143 (1.4%)** | **4/143 (2.8%)** | **RR 0.50 (0.09 to 2.65)** | **14 fewer per 1,000 (from 25 fewer to 46 more)** | **⨁◯◯◯ Very low** | **NOT IMPORTANT** |

**CI: confidence interval; MD: mean difference; RR: risk ratio**

**Explanations**

a. Included studies have the potential risk of selection bias (weight >20%)

b. Downgraded by one level due to imprecision: effect could be both benefit and harmful.

c. Although included studies have a high risk of performance bias, the outcome is unlikely to be affected.

d. Downgraded by two levels due to imprecision: low event rate and effect could be both beneficial and harmful.

e. Included study also reported effect as Mean Difference, with absolute effect 0, (0.06 lower to 0.06 higher).

f. Downgraded by two levels due to imprecision: small sample size and effect could be both beneficial and harmful.

# PICO 4 Is early initiation of GnRH-ant during stimulation recommended?

## 4.1 Normal Ovarian Responses

Question: Early administration compared to Late administration for IVF assisted pregnancy patients ? (Normal Ovarian Responses)

| **Certainty assessment** | | | | | | | **№ of patients** | | **Effect** | | **Certainty** | **Importance** |
| --- | --- | --- | --- | --- | --- | --- | --- | --- | --- | --- | --- | --- |
| **№ of studies** | **Study design** | **Risk of bias** | **Inconsistency** | **Indirectness** | **Imprecision** | **Other considerations** | **Early administration** | **Flexible administration** | **Relative (95% CI)** | **Absolute (95% CI)** |  |  |
| **Live births rate - NOR-favour C** | | | | | | | | | | | | |
| **1** | **randomised trials** | **not serious^a^** | **not serious** | **not serious** | **very serious^b^** | **none** | **66/308 (21.4%)** | **74/309 (23.9%)** | **RR 0.89 (0.67 to 1.20)** | **26 fewer per 1,000 (from 79 fewer to 48 more)** | **⨁⨁◯◯ Low** | **CRITICAL** |
| **Clinical pregnancy rate - NOR-favour I** | | | | | | | | | | | | |
| **1** | **randomised trials** | **serious^a^** | **not serious** | **not serious** | **very serious^b^** | **none** | **12/30 (40.0%)** | **9/30 (30.0%)** | **RR 1.33 (0.66 to 2.69)** | **99 more per 1,000 (from 102 fewer to 507 more)** | **⨁◯◯◯ Very Low** | **CRITICAL** |
| **Implantation rate - NOR-favour I** | | | | | | | | | | | | |
| **1** | **randomised trials** | **not serious^a^** | **not serious** | **not serious** | **very serious^c^** | **none** | **30** | **30** | **-** | **MD 1.11 higher (11.51 lower to 13.73 higher)** | **⨁⨁◯◯ Low** | **IMPORTANT** |
| **Fertilization rate(%) - NOR-favour C** | | | | | | | | | | | | |
| **1** | **randomised trials** | **not serious^a^** | **not serious** | **not serious** | **very serious^c^** | **none** | **30** | **30** | **-** | **MD 4.26 lower (744.34 lower to 735.82 higher)** | **⨁⨁◯◯ Low** | **IMPORTANT** |
| **Implantation rate - NOR-favour I** | | | | | | | | | | | | |
| **1** | **randomised trials** | **not serious^a^** | **not serious** | **not serious** | **very serious^b^** | **none** | **10/30 (33.3%)** | **9/30 (30.0%)** | **RR 1.11 (0.53 to 2.34)** | **33 more per 1,000 (from 141 fewer to 402 more)** | **⨁⨁◯◯ Low** | **CRITICAL** |

**CI: confidence interval; MD: mean difference; RR: risk ratio**

**Explanations**

a. Included studies have the potential risk of selection bias (weight >20%).

b. Downgraded by 2 levels due to imprecision: wide confidence interval and low event rate.

c. Downgraded by 2 levels due to imprecision: wide confidence interval and small sample size.

## 4.2 Poor Ovarian Responses

Question: Early administration compared to late administration for IVF assisted pregnancy patients (Poor Ovarian Responses)

| **Certainty assessment** | | | | | | | **№ of patients** | | **Effect** | | **Certainty** | **Importance** |
| --- | --- | --- | --- | --- | --- | --- | --- | --- | --- | --- | --- | --- |
| **№ of studies** | **Study design** | **Risk of bias** | **Inconsistency** | **Indirectness** | **Imprecision** | **Other considerations** | **Early administration** | **Flexible administration** | **Relative (95% CI)** | **Absolute (95% CI)** |  |  |
| **Implantation rate - POR-favour I** | | | | | | | | | | | | |
| **1** | **randomised trials** | **not serious^a^** | **not serious** | **not serious** | **serious^c^** | **none** | **58** | **58** | **-** | **MD 0.25 higher (0.13 higher to 0.37 higher)** | **⨁⨁⨁◯ Moderate** | **IMPORTANT** |
| **Oocytes retrieved - POR-favour I** | | | | | | | | | | | | |
| **1** | **randomised trials** | **not serious^a^** | **not serious** | **not serious** | **very serious^b^** | **none** | **58** | **58** | **-** | **MD 0.97 higher (0.01 lower to 1.95 higher)** | **⨁⨁◯◯ Low** | **IMPORTANT** |
| **Metaphase II oocytes-favour I** | | | | | | | | | | | | |
| **1** | **randomised trials** | **not serious^a^** | **not serious** | **not serious** | **serious^c^** | **none** | **58** | **58** | **-** | **MD 0.89 higher (0.02 higher to 1.76 higher)** | **⨁⨁⨁◯ Moderate** | **IMPORTANT** |
| **Top quality embryos-favour I** | | | | | | | | | | | | |
| **1** | **randomised trials** | **not serious^a^** | **not serious** | **not serious** | **very serious^b^** | **none** | **58** | **58** | **-** | **MD 0.31 higher (0.24 lower to 0.86 higher)** | **⨁⨁◯◯ Low** | **IMPORTANT** |

**CI:** confidence interval; **MD:** mean difference; **RR:** risk ratio

**Explanations**

a. Downgraded by 0.5 level due to ROB: there is a high risk of bias.

b. Downgraded by 2 levels due to imprecision: wide confidence interval and small sample size.

c. Downgraded by 1 level due to imprecision: small sample size.

## 4.3 High Ovarian Responses

Question: Early administration compared to late administration for IVF assisted pregnancy patients? (High Ovarian Responses)

| **Certainty assessment** | | | | | | | **№ of patients** | | **Effect** | | **Certainty** | **Importance** |
| --- | --- | --- | --- | --- | --- | --- | --- | --- | --- | --- | --- | --- |
| **№ of studies** | **Study design** | **Risk of bias** | **Inconsistency** | **Indirectness** | **Imprecision** | **Other considerations** | **Early administration** | **Late administration** | **Relative (95% CI)** | **Absolute (95% CI)** |  |  |
| **Live births rate-NOR, favor late** | | | | | | | | | | | | |
| **1** | **randomised trials** | **serious^a^** | **not serious** | **not serious** | **very serious^b^** | **none** | **5/12 (41.7%)** | **4/12 (33.3%)** | **RR 1.25 (0.44 to 3.55)** | **83 more per 1,000 (from 187 fewer to 850 more)** | **⨁◯◯◯ Very low** | **CRITICAL** |
| **Clinical pregnancy rate-POR+NOR+HOR, favor late** | | | | | | | | | | | | |
| **2** | **randomised trials** | **not serious^c^** | **not serious** | **not serious** | **very serious^d^** | **none** | **47/72 (65.3%)** | **40/74 (54.1%)** | **RR 1.08 (0.31 to 3.72)** | **43 more per 1,000 (from 373 fewer to 1,000 more)** | **⨁⨁◯◯ Low** | **CRITICAL** |
| **Endometrial thickness(mm)-POR+HOR, favor late** | | | | | | | | | | | | |
| **1** | **randomised trials** | **not serious** | **not serious** | **not serious** | **very serious^d^** | **none** | **60** | **62** | **-** | **MD 0.2 higher (0.49 lower to 0.89 higher)** | **⨁⨁◯◯ Low** | **NOT IMPORTANT** |
| **Oocytes retrieved** | | | | | | | | | | | | |
| **1** | **randomised trials** | **not serious** | **not serious** | **not serious** | **very serious^d^** | **none** | **60** | **62** | **-** | **MD 2.2 higher (0.21 lower to 4.61 higher)** | **⨁⨁◯◯ Low** | **IMPORTANT** |
| **Implantation rate,%-HOR favor early** | | | | | | | | | | | | |
| **1** | **randomised trials** | **serious^a^** | **not serious** | **not serious** | **serious^e^** | **none** | **12** | **12** | **-** | **MD 15.3 higher (15.03 higher to 15.57 higher)** | **⨁⨁◯◯ Low** | **IMPORTANT** |

**CI:** confidence interval; **MD:** mean difference; **RR:** risk ratio

**Explanations**

a. Included studies have the potential risk of selection bias (weight >20%).

b. Downgraded by two levels due to imprecision: low sample size, low event rate and effect could be both beneficial and harmful.

c. Included studies have the potential risk of selection bias (weight >20%). However, it did not affect the overall estimates.

d. Downgraded by two levels due to imprecision: low sample size and the effect could be both beneficial and harmful.

e. Downgraded by one level due to imprecision: very low sample size.

# PICO 5 Is the GnRHa add-on trigger recommended over the HCG trigger alone?

## 5.1 Add-on GnRHa trigger to HCG and HCG alone

5.1.1 Normal Ovarian Responses

Question: GnRHa+hCG compared to hCG for IVF assisted pregnancy patients (Normal Ovarian Responses)

| **Certainty assessment** | | | | | | | **№ of patients** | | **Effect** | | **Certainty** | **Importance** |
| --- | --- | --- | --- | --- | --- | --- | --- | --- | --- | --- | --- | --- |
| **№ of studies** | **Study design** | **Risk of bias** | **Inconsistency** | **Indirectness** | **Imprecision** | **Other considerations** | **GnRHa+hCG** | **hCG** | **Relative (95% CI)** | **Absolute (95% CI)** |  |  |
| **Cumulative live birth rate - NOR, favor I** | | | | | | | | | | | | |
| **1** | **randomised trials** | **not serious** | **not serious** | **not serious** | **very serious^a^** | **none** | **33/74 (44.6%)** | **24/75 (32.0%)** | **RR 1.39 (0.92 to 2.11)** | **125 more per 1,000 (from 26 fewer to 355 more)** | **⨁⨁◯◯ Low** | **CRITICAL** |
| **Live birth rate-NOR, favor I** | | | | | | | | | | | | |
| **3** | **randomised trials** | **not serious** | **not serious** | **not serious** | **serious^b^** | **none** | **88/231 (38.1%)** | **68/247 (27.5%)** | **RR 1.37 (0.97 to 1.95)** | **102 more per 1,000 (from 8 fewer to 262 more)** | **⨁⨁⨁◯ Moderate** | **CRITICAL** |
| **Implantation rate - NOR, favor I** | | | | | | | | | | | | |
| **7** | **randomised trials** | **serious^c^** | **not serious** | **not serious** | **very serious^d^** | **none** | **234/861 (27.2%)** | **176/828 (21.3%)** | **RR 1.25 (0.98 to 1.61)** | **53 more per 1,000 (from 4 fewer to 130 more)^e^** | **⨁◯◯◯ Very low** | **CRITICAL** |
| **Clinical pregnancy rate-NOR, favor I** | | | | | | | | | | | | |
| **8** | **randomised trials** | **not serious** | **not serious** | **not serious** | **serious^d^** | **none** | **245/587 (41.7%)** | **197/562 (35.1%)** | **RR 1.20 (1.03 to 1.39)** | **70 more per 1,000 (from 11 more to 137 more)** | **⨁⨁⨁◯ Moderate** | **CRITICAL** |
| **Metaphase II oocytes-NOR, favor I** | | | | | | | | | | | | |
| **8** | **randomised trials** | **not serious** | **serious^f^** | **not serious** | **not serious** | **none** | **619** | **607** | **-** | **MD 0.82 higher (0.2 higher to 1.44 higher)** | **⨁⨁⨁◯ Moderate** | **CRITICAL** |
| **Miscarriage rate-NOR, favor I** | | | | | | | | | | | | |
| **5** | **randomised trials** | **not serious** | **serious^f^** | **not serious** | **very serious^a^** | **none** | **21/233 (9.0%)** | **25/211 (11.8%)** | **RR 0.73 (0.37 to 1.42)** | **32 fewer per 1,000 (from 75 fewer to 50 more)** | **⨁◯◯◯ Very low** | **IMPORTANT** |

**CI: confidence interval; MD: mean difference; RR: risk ratio**

**Explanations**

a. Downgraded by two levels due to imprecision: low event rate and effect could be both beneficial and harmful.

b. Downgraded by one level due to imprecision: low event rate.

c. Included studies have the potential risk of selection bias (weight >20%).

d. Downgraded by one level due to imprecision: the effect could be both beneficial and harmful.

e. Another study evaluated the mean difference in implantation rate, and the MD is 2(95%CI 1.90-2.10).

f. Downgraded by one level due to inconsistency: we explored the clinical, methdological and statistical characteristics of the studies, but the unexplained heterogeneity remained. This potentially reduces our certainty on the observed magnitude of effect.

5.1.2 Poor Ovarian Responses

Question: GnRHa+hCG compared to hCG for IVF assisted pregnancy patients (Poor Ovarian Responses)

| **Certainty assessment** | | | | | | | **№ of patients** | | **Effect** | | **Certainty** | **Importance** |
| --- | --- | --- | --- | --- | --- | --- | --- | --- | --- | --- | --- | --- |
| **№ of studies** | **Study design** | **Risk of bias** | **Inconsistency** | **Indirectness** | **Imprecision** | **Other considerations** | **GnRHa+hCG** | **hCG** | **Relative (95% CI)** | **Absolute (95% CI)** |  |  |
| **Clinical pregnancy rate - POR** | | | | | | | | | | | | |
| **1** | **randomised trials** | **serious^b^** | **not serious** | **not serious** | **very serious^a^** | **none** | **13/41 (31.7%)** | **7/41 (17.1%)** | **RR 1.86 (0.83 to 4.18)** | **147 more per 1,000 (from 29 fewer to 543 more)** | **⨁◯◯◯ Very low** | **CRITICAL** |
| **Miscarriage rate - POR** | | | | | | | | | | | | |
| **1** | **randomised trials** | **serious^b^** | **not serious** | **not serious** | **very serious^a^** | **none** | **5/41 (12.2%)** | **9/41 (22.0%)** | **RR 0.56 (0.20 to 1.52)** | **97 fewer per 1,000 (from 176 fewer to 114 more)** | **⨁◯◯◯ Very low** | **IMPORTANT** |

**CI: confidence interval; MD: mean difference; RR: risk ratio**

**Explanations**

a. Downgraded by two levels due to imprecision: low event rate and effect could be both beneficial and harmful.

b. Included studies have the potential risk of selection bias (weight >20%).

## 5.2 GnRHa and HCG alone

5.2.1 Normal Ovarian Responses

Question: GnRHa compared to hCG for IVF assisted pregnancy patients (Poor Ovarian Responses)

| **Certainty assessment** | | | | | | | **№ of patients** | | **Effect** | | **Certainty** | **Importance** |
| --- | --- | --- | --- | --- | --- | --- | --- | --- | --- | --- | --- | --- |
| **№ of studies** | **Study design** | **Risk of bias** | **Inconsistency** | **Indirectness** | **Imprecision** | **Other considerations** | **GnRHa** | **hCG** | **Relative (95% CI)** | **Absolute (95% CI)** |  |  |
| **Implantation rate-NOR, favor HCG** | | | | | | | | | | | | |
| **2** | **randomised trials** | **not serious^c^** | **not serious** | **not serious** | **serious^d^** | **none** | **27/188 (14.4%)** | **65/210 (31.0%)** | **RR 0.31 (0.03 to 3.04)** | **214 fewer per 1,000 (from 300 fewer to 631 more)^e^** | **⨁⨁⨁◯ Moderate** | **CRITICAL** |
| **Clinical pregnancy rate-NOR, favor HCG** | | | | | | | | | | | | |
| **2** | **randomised trials** | **not serious^c^** | **not serious** | **not serious** | **serious^d^** | **none** | **26/134 (19.4%)** | **56/157 (35.7%)** | **RR 0.38 (0.07 to 2.18)** | **221 fewer per 1,000 (from 332 fewer to 421 more)** | **⨁⨁⨁◯ Moderate** | **CRITICAL** |
| **Metaphase II oocytes-NOR** | | | | | | | | | | | | |
| **2** | **randomised trials** | **serious^a^** | **not serious** | **not serious** | **very serious^b^** | **none** | **185** | **187** | **-** | **MD 0.35 higher (0.19 lower to 0.88 higher)** | **⨁◯◯◯ Very low** | **CRITICAL** |
| **Miscarriage rate-NOR, favor HCG** | | | | | | | | | | | | |
| **4** | **randomised trials** | **serious^a^** | **not serious** | **not serious** | **serious^d^** | **none** | **28/67 (41.8%)** | **12/97 (12.4%)** | **RR 4.42 (1.10 to 17.67)** | **423 more per 1,000 (from 12 more to 1,000 more)** | **⨁⨁◯◯ Low** | **IMPORTANT** |
| **Endometrial thickness (cm) day after hCG/GnRH-NOR, favor GnRHa** | | | | | | | | | | | | |
| **1** | **randomised trials** | **serious^a^** | **not serious** | **not serious** | **very serious^f^** | **none** | **84** | **95** | **-** | **MD 0.01 higher (0 to 0.02 higher)** | **⨁◯◯◯ Very low** | **NOT IMPORTANT** |

**Question: GnRHa compared to hCG for IVF assisted pregnancy patients (NOR)**

**CI: confidence interval; MD: mean difference; RR: risk ratio**

**Explanations**

a. One study has a potential risk of selection bias. However, the effect is unlikely to be affected.

b. Downgraded by two levels due to imprecision: low event rate and effect could be both beneficial or harmful.

c. Downgraded by one level due to imprecision: effect could be both beneficial or harmful.

# PICO 6 For individuals undergoing fresh embryo transfer with GnRHa trigger, is reinforced luteal support recommended?

Question: Enhanced luteal support compared to Standard luteal support for IVF cycle patients?

| **Certainty assessment** | | | | | | | **№ of patients** | | **Effect** | | **Certainty** | **Importance** |
| --- | --- | --- | --- | --- | --- | --- | --- | --- | --- | --- | --- | --- |
| **№ of studies** | **Study design** | **Risk of bias** | **Inconsistency** | **Indirectness** | **Imprecision** | **Other considerations** | **Enhanced luteal support** | **Standard luteal support** | **Relative (95% CI)** | **Absolute (95% CI)** |  |  |
| **Ongoing pregnancies: favour I** | | | | | | | | | | | | |
| **1** | **non-randomised studies** | **serious^a^** | **not serious** | **not serious** | **serious^b^** | **none** | **12/24 (50.0%)** | **23/91 (25.3%)** | **RR 1.98 (1.16 to 3.37)** | **248 more per 1,000 (from 40 more to 599 more)** | **⨁◯◯◯ Very low** | **CRITICAL** |
| **Early pregnancy losses: favour I** | | | | | | | | | | | | |
| **1** | **non-randomised studies** | **serious^a^** | **not serious** | **not serious** | **very serious^c^** | **none** | **3/24 (12.5%)** | **32/91 (35.2%)** | **RR 0.36 (0.12 to 1.06)** | **225 fewer per 1,000 (from 309 fewer to 21 more)** | **⨁◯◯◯ Very low** | **CRITICAL** |
| **Oocytes retrieved** | | | | | | | | | | | | |
| **1** | **non-randomised studies** | **serious^a^** | **not serious** | **not serious** | **very serious^d^** | **none** | **24** | **91** | **-** | **MD 1.7 lower (6.42 lower to 3.02 higher)** | **⨁◯◯◯ Very low** | **IMPORTANT** |

**CI: confidence interval; MD: mean difference; RR: risk ratio**

**Explanations**

a. Downgraded by 1 level due to ROB: A retrospective cohort study, non-randomized controlled study.

b. Downgraded by 1 level due to imprecision: low event rate.

c. Downgraded by 2 levels due to imprecision: wide confidence interval and low event rate.

d. Downgraded by 2 levels due to imprecision: wide confidence interval and small sample size.

# PICO 7 Fresh embryo transfer compared with frozen-all strategy for GnRH-ant protocol?

## 7.1 Normal Ovarian Responses

Question: Fresh embryo transfer compared to Frozen embryo transfer for IVF assisted pregnancy patients (Normal Ovarian Responses)

| **Certainty assessment** | | | | | | | **№ of patients** | | **Effect** | | **Certainty** | **Importance** |
| --- | --- | --- | --- | --- | --- | --- | --- | --- | --- | --- | --- | --- |
| **№ of studies** | **Study design** | **Risk of bias** | **Inconsistency** | **Indirectness** | **Imprecision** | **Other considerations** | **Fresh embryo transfer** | **Frozen embryo transfer** | **Relative (95% CI)** | **Absolute (95% CI)** |  |  |
| **Moderate, severe risk of OHSS-favor frozen** | | | | | | | | | | | | |
| **2** | **randomised trials** | **not serious^a^** | **not serious** | **not serious** | **serious^b^** | **none** | **31/1905 (1.6%)** | **11/1902 (0.6%)** | **RR 2.80 (1.41 to 5.56)** | **10 more per 1,000 (from 2 more to 26 more)** | **⨁⨁⨁◯ Moderate** | **CRITICAL** |
| **Implantation rate-favor frozen** | | | | | | | | | | | | |
| **2** | **randomised trials** | **not serious^a^** | **serious^e^** | **not serious** | **serious^d^** | **none** | **1263/2860 (44.2%)** | **1333/2804 (47.5%)** | **RR 0.90 (0.68 to 1.18)** | **48 fewer per 1,000 (from 152 fewer to 86 more)** | **⨁⨁◯◯ Low** | **CRITICAL** |
| **Clinical pregnancy rate-favor frozen** | | | | | | | | | | | | |
| **3** | **randomised trials** | **not serious^a^** | **serious^e^** | **not serious** | **serious^d^** | **none** | **1033/1941 (53.2%)** | **1113/1938 (57.4%)** | **RR 0.94 (0.73 to 1.21)** | **34 fewer per 1,000 (from 155 fewer to 121 more)** | **⨁⨁◯◯ Low** | **CRITICAL** |
| **Pre-eclampsia-favor fresh** | | | | | | | | | | | | |
| **3** | **randomised trials** | **not serious^a^** | **not serious** | **not serious** | **very serious^c^** | **none** | **28/1071 (2.6%)** | **46/1155 (4.0%)** | **RR 0.64 (0.37 to 1.11)** | **14 fewer per 1,000 (from 25 fewer to 4 more)** | **⨁⨁◯◯ Low** | **CRITICAL** |
| **Gestational hypertension-favor fresh** | | | | | | | | | | | | |
| **3** | **randomised trials** | **not serious^a^** | **not serious** | **not serious** | **very serious^c^** | **none** | **17/1071 (1.6%)** | **20/1155 (1.7%)** | **RR 0.96 (0.50 to 1.85)** | **1 fewer per 1,000 (from 9 fewer to 15 more)** | **⨁⨁◯◯ Low** | **IMPORTANT** |
| **Live birth rate-favor frozen** | | | | | | | | | | | | |
| **4** | **randomised trials** | **not serious^a^** | **serious^e^** | **not serious** | **not serious** | **none** | **964/2171 (44.4%)** | **1040/2161 (48.1%)** | **RR 0.92 (0.87 to 0.98)** | **39 fewer per 1,000 (from 63 fewer to 10 fewer)** | **⨁⨁⨁◯ Moderate** | **CRITICAL** |

**CI: confidence interval; MD: mean difference; RR: risk ratio**

**Explanations**

a. Downgraded by 0.5 level due to ROB: there are high risk of bias in blind domains

b. Downgraded by 1 levels due to imprecision: very low event rate.

c. Downgraded by 2 levels due to imprecision: wide confidence interval and low event rate.

d. Downgraded by 1 level due to imprecision: wide confidence interval.

e. Downgraded by 1 level due to indirectness: I^2^>80%.

### 7.1.1 Blastocyst stage - single embryo transfer

| **Certainty assessment** | | | | | | | **№ of patients** | | **Effect** | | **Certainty** | **Importance** |
| --- | --- | --- | --- | --- | --- | --- | --- | --- | --- | --- | --- | --- |
| **№ of studies** | **Study design** | **Risk of bias** | **Inconsistency** | **Indirectness** | **Imprecision** | **Other considerations** | **Fresh embryo transfer** | **Frozen embryo transfer-NOR-使用** | **Relative (95% CI)** | **Absolute (95% CI)** |  |  |
| **Moderate, severe risk of OHSS** | | | | | | | | | | | | |
| **1** | **randomised trials** | **not serious^a^** | **not serious** | **not serious** | **very serious^b^** | **none** | **9/825 (1.1%)** | **4/825 (0.5%)** | **RR 2.25 (0.70 to 7.28)** | **6 more per 1,000 (from 1 fewer to 30 more)** | **⨁⨁◯◯ Low** | **CRITICAL** |
| **Live birth rate** | | | | | | | | | | | | |
| **2** | **randomised trials** | **not serious^a^** | **serious^d^** | **not serious** | **serious^c^** | **none** | **407/1055 (38.6%)** | **500/1048 (47.7%)** | **RR 0.87 (0.65 to 1.17)** | **62 fewer per 1,000 (from 167 fewer to 81 more)** | **⨁⨁◯◯ Low** | **CRITICAL** |
| **Implantation rate** | | | | | | | | | | | | |
| **1** | **randomised trials** | **not serious^a^** | **not serious** | **not serious** | **not serious** | **none** | **406/833 (48.7%)** | **524/838 (62.5%)** | **RR 0.78 (0.71 to 0.85)** | **138 fewer per 1,000 (from 181 fewer to 94 fewer)** | **⨁⨁⨁⨁ High** | **CRITICAL** |
| **Clinical pregnancy rate** | | | | | | | | | | | | |
| **2** | **randomised trials** | **serious^e^** | **not serious** | **not serious** | **serious^c^** | **none** | **418/861 (48.5%)** | **527/861 (61.2%)** | **RR 0.86 (0.63 to 1.18)** | **86 fewer per 1,000 (from 226 fewer to 110 more)** | **⨁⨁◯◯ Low** | **CRITICAL** |
| **Pre-eclampsia** | | | | | | | | | | | | |
| **2** | **randomised trials** | **not serious^a^** | **not serious** | **not serious** | **very serious^b^** | **none** | **8/456 (1.8%)** | **20/569 (3.5%)** | **RR 0.54 (0.17 to 1.71)** | **16 fewer per 1,000 (from 29 fewer to 25 more)** | **⨁⨁◯◯ Low** | **CRITICAL** |
| **Gestational hypertension** | | | | | | | | | | | | |
| **2** | **randomised trials** | **not serious^a^** | **not serious** | **not serious** | **very serious^b^** | **none** | **10/456 (2.2%)** | **15/569 (2.6%)** | **RR 0.82 (0.37 to 1.82)** | **5 fewer per 1,000 (from 17 fewer to 22 more)** | **⨁⨁◯◯ Low** | **IMPORTANT** |

**CI: confidence interval; MD: mean difference; RR: risk ratio**

**Explanations**

a. Downgraded by 0.5 level due to ROB: there are high risk of bias in blind domains

b. Downgraded by 2 levels due to imprecision: wide confidence interval and low event rate.

c. Downgraded by 1 level due to imprecision: wide confidence interval.

d. Downgraded by one level due to ROB and indirectness: downgraded 0.5 level due to there were high risk of bias in blind domains; downgraded 0.5 level due to heterogeneity.

e. Downgraded by 1 level due to ROB: there are high risk of bias in blind domains, large sample size and direction inconsistency.

### 7.1.2 Cleavage stage - multiple embryo transfer.

| **Certainty assessment** | | | | | | | **№ of patients** | | **Effect** | | **Certainty** | **Importance** |
| --- | --- | --- | --- | --- | --- | --- | --- | --- | --- | --- | --- | --- |
| **№ of studies** | **Study design** | **Risk of bias** | **Inconsistency** | **Indirectness** | **Imprecision** | **Other considerations** | **Fresh embryo transfer** | **Frozen embryo transfer** | **Relative (95% CI)** | **Absolute (95% CI)** |  |  |
| **Moderate, severe risk of OHSS** | | | | | | | | | | | | |
| **1** | **randomised trials** | **not serious^a^** | **not serious** | **not serious** | **serious^b^** | **none** | **22/1080 (2.0%)** | **7/1077 (0.6%)** | **RR 3.13 (1.34 to 7.31)** | **14 more per 1,000 (from 2 more to 41 more)** | **⨁⨁⨁◯ Moderate** | **CRITICAL** |
| **Live birth rate-** | | | | | | | | | | | | |
| **1** | **randomised trials** | **not serious^a^** | **not serious** | **not serious** | **serious^d^** | **none** | **542/1080 (50.2%)** | **525/1077 (48.7%)** | **RR 1.03 (0.95 to 1.12)** | **15 more per 1,000 (from 24 fewer to 58 more)** | **⨁⨁⨁◯ Moderate** | **CRITICAL** |
| **Implantation rate** | | | | | | | | | | | | |
| **1** | **randomised trials** | **not serious^a^** | **not serious** | **not serious** | **serious^d^** | **none** | **857/2027 (42.3%)** | **809/1966 (41.1%)** | **RR 1.03 (0.95 to 1.11)** | **12 more per 1,000 (from 21 fewer to 45 more)** | **⨁⨁⨁◯ Moderate** | **CRITICAL** |
| **Clinical pregnancy rate-D3 vs. D5** | | | | | | | | | | | | |
| **1** | **randomised trials** | **not serious^a^** | **not serious** | **not serious** | **serious^d^** | **none** | **615/1080 (56.9%)** | **586/1077 (54.4%)** | **RR 1.05 (0.97 to 1.13)** | **27 more per 1,000 (from 16 fewer to 71 more)** | **⨁⨁⨁◯ Moderate** | **CRITICAL** |
| **Pre-eclampsia** | | | | | | | | | | | | |
| **1** | **randomised trials** | **not serious^a^** | **not serious** | **not serious** | **very serious^c^** | **none** | **20/615 (3.3%)** | **26/586 (4.4%)** | **RR 0.73 (0.41 to 1.30)** | **12 fewer per 1,000 (from 26 fewer to 13 more)** | **⨁⨁◯◯ Low** | **CRITICAL** |
| **Gestational hypertension** | | | | | | | | | | | | |
| **1** | **randomised trials** | **not serious^a^** | **not serious** | **not serious** | **very serious^c^** | **none** | **7/615 (1.1%)** | **5/586 (0.9%)** | **RR 1.33 (0.43 to 4.18)** | **3 more per 1,000 (from 5 fewer to 27 more)** | **⨁⨁◯◯ Low** | **IMPORTANT** |

**CI: confidence interval; RR: risk ratio**

**Explanations**

a. Downgraded by 0.5 level due to ROB: there are high risk of bias in blind domains

b. Downgraded by 1 levels due to imprecision: very low event rate.

c. Downgraded by 2 levels due to imprecision: wide confidence interval and low event rate.

d. Downgraded by 1 level due to imprecision: wide confidence interval.

## 7.2 High Ovarian Responses

Question: Should GnRH-ant be used for IVF cycle patients compared to GnRH-a? (High Ovarian Responses )

| **Certainty assessment** | | | | | | | **№ of patients** | | **Effect** | | **Certainty** | **Importance** |
| --- | --- | --- | --- | --- | --- | --- | --- | --- | --- | --- | --- | --- |
| **№ of studies** | **Study design** | **Risk of bias** | **Inconsistency** | **Indirectness** | **Imprecision** | **Other considerations** | **Fresh embryo transfer** | **Frozen embryo transfer** | **Relative (95% CI)** | **Absolute (95% CI)** |  |  |
| **Moderate, severe risk of OHSS-favor frozen** | | | | | | | | | | | | |
| **3** | **randomised trials** | **not serious^a^** | **serious^b^** | **not serious** | **very serious^c^** | **none** | **70/986 (7.1%)** | **17/971 (1.8%)** | **RR 3.45 (0.80 to 14.80)** | **43 more per 1,000 (from 4 fewer to 242 more)** | **⨁◯◯◯ Very low** | **CRITICAL** |
| **Live birth rate-favor frozen** | | | | | | | | | | | | |
| **3** | **randomised trials** | **not serious^a^** | **not serious** | **not serious** | **not serious** | **none** | **393/985 (39.9%)** | **443/968 (45.8%)** | **RR 0.87 (0.78 to 0.96)** | **59 fewer per 1,000 (from 101 fewer to 18 fewer)** | **⨁⨁⨁⨁ High** | **CRITICAL** |
| **Clinical pregnancy rate-favor frozen** | | | | | | | | | | | | |
| **3** | **randomised trials** | **not serious^a^** | **not serious** | **not serious** | **serious^d^** | **none** | **520/986 (52.7%)** | **534/971 (55.0%)** | **RR 0.96 (0.88 to 1.04)** | **22 fewer per 1,000 (from 66 fewer to 22 more)** | **⨁⨁⨁◯ Moderate** | **CRITICAL** |

**CI: confidence interval; RR: risk ratio**

**Explanations**

a. Downgraded by 0.5 level due to ROB: there are high risk of bias in blind domains

b. Downgraded by one level due to ROB and indirectness: downgraded 0.5 level due to there were high risk of bias in blind domains; downgraded 0.5 level due to heterogeneity.

c. Downgraded by 2 levels due to imprecision: wide confidence interval and low event rate.

d. Downgraded by 1 level due to imprecision: wide confidence interval.

### 7.2.1 Blastocyst stage - single embryo transfer

| **Certainty assessment** | | | | | | | **№ of patients** | | **Effect** | | **Certainty** | **Importance** |
| --- | --- | --- | --- | --- | --- | --- | --- | --- | --- | --- | --- | --- |
| **№ of studies** | **Study design** | **Risk of bias** | **Inconsistency** | **Indirectness** | **Imprecision** | **Other considerations** | **Fresh embryo transfer** | **Frozen embryo transfer** | **Relative (95% CI)** | **Absolute (95% CI)** |  |  |
| **Moderate, severe risk of OHSS** | | | | | | | | | | | | |
| **1** | **randomised trials** | **not serious** | **not serious** | **not serious** | **serious^b^** | **none** | **9/105 (8.6%)** | **0/104 (0.0%)** | **RR 18.82 (1.11 to 319.24)** | **0 fewer per 1,000 (from 0 fewer to 0 fewer)** | **⨁⨁⨁◯ Moderate** | **CRITICAL** |
| **Live birth rate** | | | | | | | | | | | | |
| **1** | **randomised trials** | **not serious** | **not serious** | **not serious** | **very serious^a^** | **none** | **41/104 (39.4%)** | **42/101 (41.6%)** | **RR 0.95 (0.68 to 1.32)** | **21 fewer per 1,000 (from 133 fewer to 133 more)** | **⨁⨁◯◯ Low** | **CRITICAL** |
| **Clinical pregnancy rate** | | | | | | | | | | | | |
| **1** | **randomised trials** | **not serious** | **not serious** | **not serious** | **very serious^a^** | **none** | **51/105 (48.6%)** | **57/104 (54.8%)** | **RR 0.89 (0.68 to 1.15)** | **60 fewer per 1,000 (from 175 fewer to 82 more)** | **⨁⨁◯◯ Low** | **CRITICAL** |

**CI: confidence interval; RR: risk ratio**

**Explanations**

a. Downgraded by 2 levels due to imprecision: wide confidence interval and low event rate.

b. Downgraded by 1 levels due to imprecision: very low event rate.

### 7.2.2 Cleavage stage - multiple embryo transfer.

| **Certainty assessment** | | | | | | | **№ of patients** | | **Effect** | | **Certainty** | **Importance** |
| --- | --- | --- | --- | --- | --- | --- | --- | --- | --- | --- | --- | --- |
| **№ of studies** | **Study design** | **Risk of bias** | **Inconsistency** | **Indirectness** | **Imprecision** | **Other considerations** | **Fresh embryo transfer** | **Frozen embryo transfer** | **Relative (95% CI)** | **Absolute (95% CI)** |  |  |
| **Moderate, severe risk of OHSS** | | | | | | | | | | | | |
| **2** | **randomised trials** | **not serious^a^** | **serious^c^** | **not serious** | **very serious^b^** | **none** | **61/881 (6.9%)** | **17/867 (2.0%)** | **RR 2.43 (0.48 to 12.34)** | **28 more per 1,000 (from 10 fewer to 222 more)** | **⨁◯◯◯ Very low** | **CRITICAL** |
| **Live birth rate** | | | | | | | | | | | | |
| **2** | **randomised trials** | **not serious^a^** | **not serious** | **not serious** | **not serious** | **none** | **352/881 (40.0%)** | **401/867 (46.3%)** | **RR 0.86 (0.77 to 0.96)** | **65 fewer per 1,000 (from 106 fewer to 19 fewer)** | **⨁⨁⨁⨁ High** | **CRITICAL** |
| **Clinical pregnancy rate** | | | | | | | | | | | | |
| **2** | **randomised trials** | **not serious^a^** | **not serious** | **not serious** | **serious^d^** | **none** | **469/881 (53.2%)** | **477/867 (55.0%)** | **RR 0.96 (0.88 to 1.05)** | **22 fewer per 1,000 (from 66 fewer to 28 more)** | **⨁⨁⨁◯ Moderate** | **CRITICAL** |
| **Gestational hypertension** | | | | | | | | | | | | |
| **1** | **randomised trials** | **not serious^a^** | **not serious** | **not serious** | **very serious^b^** | **none** | **5/427 (1.2%)** | **10/434 (2.3%)** | **RR 0.51 (0.18 to 1.47)** | **11 fewer per 1,000 (from 19 fewer to 11 more)** | **⨁⨁◯◯ Low** | **IMPORTANT** |
| **Preterm birth** | | | | | | | | | | | | |
| **2** | **randomised trials** | **not serious^a^** | **not serious** | **not serious** | **very serious^b^** | **none** | **74/459 (16.1%)** | **88/467 (18.8%)** | **RR 0.86 (0.65 to 1.13)** | **26 fewer per 1,000 (from 66 fewer to 24 more)** | **⨁⨁◯◯ Low** | **CRITICAL** |

**CI: confidence interval; RR: risk ratio**

**Explanations**

a. Downgraded by 0.5 level due to ROB: there are high risk of bias in blind domains

b. Downgraded by 2 levels due to imprecision: wide confidence interval and low event rate.

c. Downgraded by 1 level due to inconsistency: the I^2^ value is high (I^2^ > 80%). We explored the clinical, methdological and statistical characteristics of the studies, but the unexplained heterogeneity remained. This potentially reduces our certainty on the observed magnitude of effect.

d. Downgraded by 1 level due to imprecision: wide confidence interval.

# PICO 8 Which is the preferred timing to perform the embryo transfer: Day 3 or Day 5?

| **Certainty assessment** | | | | | | | **№ of patients** | | **Effect** | | **Certainty** | **Importance** |
| --- | --- | --- | --- | --- | --- | --- | --- | --- | --- | --- | --- | --- |
| **№ of studies** | **Study design** | **Risk of bias** | **Inconsistency** | **Indirectness** | **Imprecision** | **Other considerations** | **Cleavage embryo transfer** | **Blastocyst embryo transfer** | **Relative (95% CI)** | **Absolute (95% CI)** |  |  |
| **Clinical pregnancy rate** | | | | | | | | | | | | |
| **1** | **randomised trials** | **not serious** | **not serious** | **not serious** | **serious^a^** | **none** | **41/176 (23.3%)** | **58/175 (33.1%)** | **RR 0.70 (0.50 to 0.99)** | **99 fewer per 1,000 (from 166 fewer to 3 fewer)** | **⨁⨁⨁◯ Moderate** | **CRITICAL** |
| **Ectopic pregnancy** | | | | | | | | | | | | |
| **1** | **randomised trials** | **not serious** | **not serious** | **not serious** | **very serious^b^** | **none** | **1/59 (1.7%)** | **1/73 (1.4%)** | **RR 1.24 (0.08 to 19.36)** | **3 more per 1,000 (from 13 fewer to 252 more)** | **⨁⨁◯◯ Low** | **IMPORTANT** |
| **Multiple births** | | | | | | | | | | | | |
| **1** | **randomised trials** | **not serious** | **not serious** | **not serious** | **very seriousb** | **none** | **2/41 (4.9%)** | **0/58 (0.0%)** | **RR 7.02 (0.35 to 142.56)** | **0 fewer per 1,000 (from 0 fewer to 0 fewer)** | **⨁⨁◯◯ Low** | **NOT IMPORTANT** |

**CI: confidence interval; MD: mean difference; RR: risk ratio**

**Explanations**

a. Downgraded by one level due to imprecision: low event rate.

b. Downgraded by two level due to imprecision: wide confidence interval and low event rate.
