# Supplementary material for: Recommendations for the management of GnRH‐ant for ovarian stimulation in the assistant reproductive process: Benefits and harms for different ovarian response population
Source: Clin Public Health Guidel. 2025 May 21;2(3):e70022. doi: 10.1002/gin2.70022 (PMC13563367; doi:10.1002/gin2.70022)
Supplement: Supplementary file 5 [file GIN2-2-e70022-s006.docx]

Appendix 1. Clinical Specialty

| **Name** | **Institution** | **Geographical location** | **Content expertise** | **Role in the GDG** |
| --- | --- | --- | --- | --- |
| Li Rong | Peking University Third Hospital | Beijing | Reproductive Medicine | President |
| Yang Rui | Peking University Third Hospital | Beijing | Reproductive Medicine | Member |
| Cao Yun-xia | The First Affiliated Hospital of Anhui Medical University | Hefei, Anhui | Reproductive Medicine | Member |
| Chen Zi-jiang | Reproductive Hospital Affiliated to Shandong University | Jinan, Shandong | Obstetrics and gynecology, reproductive medicine | Member |
| Guan Yi-chun | The Third Affiliated Hospital of Zhengzhou University | Zhengzhou, Henan | Reproductive Medicine | Member |
| Gong Fei | CITIC Xiangya Reproductive and Genetic Hospital | Changsha, Hunan | Reproductive Center | Member |
| Hao Gui-min | Hebei Medical University Second Hospital | Shijiazhuang, Hebei | Reproductive Medicine | Member |
| Huang He-feng | Fudan University Obstetrics and Gynecology Hospital | Shanghai | Gynecological Endocrinology and Reproductive Medicine | Member |
| Jin Li | Fudan University Obstetrics and Gynecology Hospital | Shanghai | Gynecological Endocrinology and Reproductive Medicine | Member |
| Liu Feng-hua | Guangdong Maternal and Child Health Hospital | Guangzhou，  Guangdong | Reproductive Health and Infertility | Member |
| Liu Jia-yin | Jiangsu Provincial People’s Hospital | Nanjing, Jiangsu | Clinical Reproductive Medicine | Member |
| Liang Xiao-yan | Sun Yat-sen University The Sixth Affiliated Hospital of Xinjiang Medical University | Guangzhou, Guangdong | Reproductive Medicine | Member |
| La Xiao-lin | The First Affiliated Hospital of Xinjiang Medical University | Urumqi, Xinjiang Uygur Autonomous Region | Reproductive medicine, reproductive genetics and stem cell research | Member |
| Sun Yun | Renji Hospital Affiliated to Shanghai Jiao Tong University | Shanghai | Reproductive Medicine | Member |
| Wang Xiao-hong | Tangdu Air Force Medical University Hospital | Xi’an, Shaanxi | Reproductive Medicine | Member |
| Xu Yan-wen | The First Affiliated Hospital of Sun Yat-sen University | Guangzhou, Guangdong | Reproductive Medicine | Member |
| Yan Jun-hao | Reproductive Hospital Affiliated to Shandong University | Jinan,  Shandong | Reproductive Medicine | Member |
| Zhang Cui-lian | Henan Provincial People’s Hospital | Zhengzhou, Henan | Reproductive Medicine | Member |
| Qiao Jie | Peking University Third Hospital | Beijing | Obstetrics and Gynecology | Member |
