# Supplementary material for: Recommendations for the management of GnRH‐ant for ovarian stimulation in the assistant reproductive process: Benefits and harms for different ovarian response population
Source: Clin Public Health Guidel. 2025 May 21;2(3):e70022. doi: 10.1002/gin2.70022 (PMC13563367; doi:10.1002/gin2.70022)
Supplement: Supplementary file 6 [file GIN2-2-e70022-s007.docx]

**Appendix 2 Clinical Questions and PICOs**

**PICO 1. whether the GnRH-ant protocol is recommended over the GnRH agonist (GnRH-a) protocols?**

| Population | All IVF-assisted pregnancy patients in the general population | | | |
| --- | --- | --- | --- | --- |
| Intervention | COS stimulation antagonists regimen (any relevant medications, dosages, treatment durations) | | | |
| Comparator | COS stimulation agonists regimen (any relevant medications, dosages, treatment durations) | | | |
| Outcome | Critical  Live birth rate (including cumulative live birth rate, birth defect incidence);  Incidence of moderate to severe ovarian hyperstimulation syndrome (OHSS);Clinical pregnancy rate | | Important  Embryo implantation rate; Egg quality index; Endometrial receptivity | Low importance  Miscarriage;  Ectopic pregnancy |
| Subgroup | Population with normal ovarian response (NOR), poor ovarian response (POR), and high ovarian response (HOR) (as defined in the attachment); Cetrorelix acetate group and Ganirelix acetate group. | | | |
| Notes |  | | | |
| Prioritization scores for outcomes:  PICO1   Clinical pregnancy rate  Miscarriage  Ectopic pregnancy  Endometrial receptivity  Egg quality index  Cumulative live birth rate  Live birth rate  Embryo implantation rate  Incidence of moderate to severe ovarian hyperstimulation syndrome (OHSS)  Cumulative clinical pregnancy rate   | | Outcome rank \| Mean value; Live birth rate \| 8.53; Cumulative live birth rate \| 8.47; Incidence of moderate to severe ovarian hyperstimulation syndrome (OHSS) \| 7.40; Clinical pregnancy rate \| 7.13; Embryo implantation rate \| 6.87; Egg quality index \| 6.67; Cumulative clinical pregnancy rate \| 6.40; Endometrial receptivity \| 6.07; Egg quality index \| 5.73; Ectopic pregnancy \| 4.27 | | |

**PICO 2: For people undergoing COS with GnRH antagonist treatment in IVF, is pretreatment recommended before implementing the antagonist protocol?**

| Population | All IVF-assisted pregnancy patients in the general population | | | |
| --- | --- | --- | --- | --- |
| Intervention | COS stimulation antagonist regimen + pretreatment (a. estrogen, b. progesterone, c. oral contraceptives, d. antagonist pretreatment) | | | |
| Comparator | COS stimulation antagonist regimen (i.e. without pretreatment) | | | |
| Outcome | Critical  Live birth rate (including cumulative live birth rate, incidence of birth defects);  Embryo implantation rate | Important  Endometrial receptivity;  Egg quality index;  Clinical pregnancy rate;  Incidence of moderate OHSS | | Low importance  Miscarriage;  Ectopic pregnancy |
| Subgroup | Population with normal ovarian response NOR, poor ovarian response POR, high ovarian response HOR | | | |
| Notes |  | | | |
| Prioritization scores for outcomes:  PICO 2    Live birth rate  Cumulative live birth rate  Cumulative clinical pregnancy rate  Clinical pregnancy rate  Incidence of moderate to severe ovarian hyperstimulation syndrome (OHSS)  Endometrial receptivity  Egg quality index  Miscarriage  Ectopic pregnancy  Embryo implantation rate   | | | Outcome rank \| Mean value; Live birth rate \| 8.20; Cumulative live birth rate \| 8.13; Embryo implantation rate \| 7.13; Endometrial receptivity \| 6.73; Egg quality index \| 6.60; Clinical pregnancy rate \| 6.53; Cumulative clinical pregnancy rate \| 6.33; Incidence of moderate to severe ovarian hyperstimulation syndrome (OHSS) \| 6.20; Miscarriage \| 5.80; Ectopic pregnancy \| 4.53 | |

**PICO 3. Should fixed regimen or flexible regimen be preferred under GnRH-ant protocol?**

| Population | All IVF-assisted pregnancy patients in the general population | | | |
| --- | --- | --- | --- | --- |
| Intervention | Fixed regimen of COS stimulation antagonists (dose, treatment duration not limited, choice of antagonist drug not limited) | | | |
| Comparator | Flexible regimen of COS stimulation antagonists (dose, treatment duration not limited, choice of antagonist drug not limited) | | | |
| Outcome | Critical  Live birth rate (including cumulative live birth rate, incidence of birth defects); Incidence of moderate OHSS; Embryo implantation rate | Important  Clinical pregnancy rate; Egg quality index; Endometrial receptivity | | Low importance  Miscarriage; Ectopic pregnancy; LH surge escape rate; Average number of retrieved oocytes; Overall embryo cryopreservation rate |
| Subgroup | NOR, POR, HOR | | | |
| Notes |  | | | |
| Prioritization scores for outcomes:  PICO 3    Live birth rate  Cumulative live birth rate  Cumulative clinical pregnancy rate  Clinical pregnancy rate  Incidence of moderate to severe ovarian hyperstimulation syndrome (OHSS)  Endometrial receptivity  Egg quality index  Miscarriage  Ectopic pregnancy  Embryo implantation rate   | | | Outcome rank \| Mean value; Live birth rate \| 8.20; Cumulative live birth rate \| 8.13; Incidence of moderate OHSS \| 7.20; Embryo implantation rate \| 7.13; Clinical pregnancy rate \| 6.93; Egg quality index \| 6.73; Cumulative clinical pregnancy rate \| 6.40; Endometrial receptivity \| 6.33; Miscarriage \| 5.60; Ectopic pregnancy \| 4.67 | |

**PICO 4. Is early initiation of GnRH-ant during stimulation recommended?**

| Population | All IVF-assisted pregnancy patients | | |
| --- | --- | --- | --- |
| Intervention | Early initiation of COS stimulation antagonists (any regimen)*  *Definition of early initiation: follicle diameter less than 12 millimeters; estradiol </= 200 picograms per milliliter (pg/ml); and antagonist initiation prior to day 5 of stimulation (excluding day 5) | | |
| Comparator | Delayed initiation of COS stimulation antagonists (any regimen) | | |
| Outcome | Critical  Live birth rate (including cumulative rate, incidence of birth defects); Clinical pregnancy rate | Important  Egg quality;  Embryo quality;  Embryo implantation rate | Low importance  Hormonal fluctuation amplitude; Number of retrieved eggs; Endometrial receptivity; Egg quality index; Miscarriage; Ectopic pregnancy; Early LH surge |
| Subgroup | None | | |
| Notes |  | | |
| Prioritization scores for outcomes:  PICO 4    Early LH surge  Egg quality  Egg quality index  Endometrial receptivity  Number of retrieved eggs  Embryo implantation rate  Embryo quality  Miscarriage  Ectopic pregnancy  Clinical pregnancy rate  Cumulative live birth rate  Live birth rate  Hormonal fluctuation amplitude  Cumulative clinical pregnancy rate   | | | Outcome rank \| Mean value; Early LH surge \| 8.21; Live birth rate \| 7.71; Cumulative live birth rate \| 7.50; Clinical pregnancy rate \| 7.00; Cumulative clinical pregnancy rate \| 6.93; Egg quality \| 6.86; Embryo quality \| 6.79; Embryo implantation rate \| 6.50; Hormonal fluctuation amplitude \| 6.50; Number of retrieved eggs \| 6.36; Endometrial receptivity \| 5.93; Egg quality index \| 5.71; Miscarriage \| 5.50; Ectopic pregnancy \| 4.71 |

**PICO 5. Is the GnRHa add-on trigger recommended over the HCG trigger alone?**

| Population | All IVF-assisted pregnancy patients | | | |
| --- | --- | --- | --- | --- |
| Intervention | 1.COS stimulation antagonist regimen with GnRH agonist trigger (unlimited dosage) as a single agent  2.COS stimulation antagonist regimen with GnRH agonist trigger (unlimited dosage) in combination with HCG (adequate dose vs small dose) | | | |
| Comparator | COS stimulation antagonist regimen with HCG trigger (unlimited dosage, any type of HCG) | | | |
| Outcome | Critical  Live birth rate (including cumulative rate, incidence of birth defects); Incidence of moderate to severe OHSS; Embryo implantation rate; Clinical pregnancy rate; Egg quality index | Important  Miscarriage | | Low importance  Ectopic pregnancy; Endometrial receptivity |
| Subgroup | Population with normal ovarian response NOR, poor ovarian response POR, and high ovarian response HOR (as defined in the attachment) | | | |
| Notes |  | | | |
| Prioritization scores for outcomes:  PICO 5    Ectopic pregnancy  Miscarriage  Endometrial receptivity  Egg quality index  Embryo implantation rate  Clinical pregnancy rate  Cumulative live birth rate  Live birth rate  Incidence of moderate to severe ovarian hyperstimulation syndrome (OHSS)  Cumulative clinical pregnancy rate   | | | Outcome rank \| Mean value; Live birth rate \| 8.40; Incidence of moderate OHSS \| 8.07; Embryo implantation rate \| 7.93; Endometrial receptivity \| 7.87; Cumulative live birth rate \| 7.73; Clinical pregnancy rate \| 7.40; Egg quality index \| 7.40; Cumulative clinical pregnancy rate \| 6.53; Miscarriage \| 6.53; Ectopic pregnancy \| 5.40 | |

**PICO 6. For individuals undergoing fresh embryo transfer with GnRHa trigger, is reinforced luteal support recommended?**

| Population | Ovarian hyper-response patients undergoing fresh embryo transfer with treatment of GnRH agonist trigger in antagonist regimen | | | |
| --- | --- | --- | --- | --- |
| Intervention | After GnRH agonist trigger in antagonist regimen, reinforcement of luteal phase support (Possible scenarios: 1. Addition of small dose of HCG or other estrogen; 2. Protocol without HCG active drug, i.e., increasing the dose of estrogen and progesterone. Note the grouping when handling data.) | | | |
| Comparator | No reinforcement of luteal phase support after GnRH agonist trigger in antagonist regimen | | | |
| Outcome | Critical  Live birth rate (including cumulative rate, incidence of birth defects);  Embryo implantation rate;  Incidence of moderate to severe OHSS; Clinical pregnancy rate; Miscarriage | Important  Egg quality index | | Low importance  Ectopic pregnancy Endometrial receptivity |
| Subgroup | None | | | |
| Notes |  | | | |
| Prioritization scores for outcomes:  PICO 6    Ectopic pregnancy  Miscarriage  Endometrial receptivity  Egg quality index  Embryo implantation rate  Clinical pregnancy rate  Cumulative live birth rate  Live birth rate  Incidence of moderate to severe ovarian hyperstimulation syndrome (OHSS)  Cumulative clinical pregnancy rate   | | | Outcome rank \| Mean value; Live birth rate \| 8.57; Embryo implantation rate \| 7.93; Endometrial receptivity \| 7.64; Cumulative live birth rate \| 7.57; Incidence of moderate OHSS \| 7.50; Clinical pregnancy rate \| 7.43; Miscarriage \| 7.36; Cumulative clinical pregnancy rate \| 6.64; Egg quality index \| 6.00; Ectopic pregnancy \| 5.14 | |

**PICO 7: Under GnRH-ant protocol, which strategy is preferable between fresh and frozen embryo transfer?**

| Population | IVF-assisted pregnancy patients undergoing treatment with COS stimulation antagonist regimen | | | |
| --- | --- | --- | --- | --- |
| Intervention | Fresh embryo transfer | | | |
| Comparator | Frozen embryo transfer | | | |
| Outcome | Critical  Incidence of moderate to severe OHSS; Live birth rate (including cumulative rate, incidence of birth defects); Embryo implantation rate; Clinical pregnancy rate; Preeclampsia; | Important  Hypertension in pregnancy | | Low importance  Preterm birth Large for gestational age infants Miscarriage Egg quality index Placental abruption Ectopic pregnancy Endometrial receptivity |
| Subgroup | Blastocyst stage (D5-6) embryo transfer, Cleavage stage (D2-3) embryo transfer; single embryo transfer, multiple embryo transfer. | | | |
| Notes |  | | | |
| Prioritization scores for outcomes:  PICO 7    Preterm birth  Placental abruption  Hypertension in pregnancy  Preeclampsia  Egg quality index  Endometrial receptivity  Ectopic pregnancy  Miscarriage  Embryo implantation rate  Cumulative live birth rate  Clinical pregnancy rate  Live birth rate  Large for gestational age infants  Cumulative clinical pregnancy rate  Incidence of moderate to severe ovarian hyperstimulation syndrome (OHSS)   | | | Outcome rank \| Mean value; Incidence of moderate OHSS \| 7.93; Live birth rate \| 7.8; Embryo implantation rate \| 7.67; Clinical pregnancy rate \| 7.33; Cumulative live birth rate \| 7.27; Preeclampsia \| 7.07; Endometrial receptivity \| 7; Hypertension in pregnancy \| 6.87; Preterm birth \| 6.87; Large for gestational age infants \| 6.73; Cumulative clinical pregnancy rate \| 6.67; Miscarriage \| 6.4; Egg quality index \| 6.07; Placental abruption \| 6; Ectopic pregnancy \| 5.73 | |

**PICO 8. Which is the preferred timing to perform the embryo transfer: Day 3 or Day 5?**

| Population | IVF-assisted pregnancy patients undergoing treatment with COS stimulation antagonist regimen | | | |
| --- | --- | --- | --- | --- |
| Intervention | Day 3 embryo transfer | | | |
| Comparator | Day 5 embryo transfer | | | |
| Outcome | Critical  Live birth rate (including cumulative rate, incidence of birth defects);  Embryo implantation rate;  Clinical pregnancy rate;  Incidence of moderate to severe OHSS | Important  Ectopic pregnancy | | Low importance Miscarriage; Egg quality index; Multiple pregnancy rate; Endometrial receptivity; |
| Subgroup | Single embryo transfer, multiple embryo transfer. | | | |
| Notes |  | | | |
| Prioritization scores for outcomes:  PICO 8    Embryo implantation rate  Endometrial receptivity  Ectopic pregnancy  Miscarriage  Egg quality index  Cumulative live birth rate  Clinical pregnancy rate  Live birth rate  Incidence of moderate to severe ovarian hyperstimulation syndrome (OHSS)  Cumulative clinical pregnancy rate   | | | Outcome rank \| Mean value; Live birth rate \| 8.27; Embryo implantation rate \| 8.07; Clinical pregnancy rate \| 7.67; Cumulative live birth rate \| 7.53; Incidence of moderate OHSS \| 7.07; Endometrial receptivity \| 7.00; Cumulative clinical pregnancy rate \| 6.80; Ectopic pregnancy \| 6.40; Miscarriage \| 6.27; Egg quality index \| 5.80 | |
