# Supplementary material for: Recommendations for the management of GnRH‐ant for ovarian stimulation in the assistant reproductive process: Benefits and harms for different ovarian response population
Source: Clin Public Health Guidel. 2025 May 21;2(3):e70022. doi: 10.1002/gin2.70022 (PMC13563367; doi:10.1002/gin2.70022)
Supplement: Supplementary file 7 [file GIN2-2-e70022-s001.doc]

# Table of contents

Table of contents	1

PICO 1. Whether the GnRH-ant protocol is recommended over the GnRH agonist (GnRH-a) protocols?	1

FigureS1.1 GnRH - ant vs GnRH - a: live birth rate	1

FigureS1.2 GnRH - ant vs GnRH - a: cumulative live birth rate	1

FigureS1.3 GnRH - ant vs GnRH - a: moderate to severe OHSS	2

FigureS1.4 GnRH - ant vs GnRH - a: clinical pregnancy rate	4

FigureS1.5 GnRH - ant vs GnRH - a: implantation rate	4

FigureS1.6 GnRH - ant vs GnRH - a: metaphase II oocytes	5

FigureS1.7 GnRH - ant vs GnRH - a: endometrial thickness	6

FigureS1.8 GnRH - ant vs GnRH - a: miscarriage rate	7

PICO 2. Among patients undergoing IVF assisted reproduction with COS using GnRH-ant, is it recommended to implement pretreatments?	1

2.1 Pretreatment with Oral Contraceptive Pill (OCP)	1

FigureS2.1.1 OCP pretreatment vs No pretreatment: metaphase II oocytes	1

FigureS2.1.2 OCP pretreatment vs No pretreatment: moderate risk of OHSS	1

FigureS2.1.3 OCP pretreatment vs no pretreatment: live birth rate	2

FigureS2.1.4 OCP pretreatment vs No pretreatment: cumulative live birth rate	2

FigureS2.1.5 OCP pretreatment vs No pretreatment: endometrial thickness	3

FigureS2.1.6 OCP pretreatment vs No pretreatment: clinical pregnancy rate	3

FigureS2.1.7 OCP pretreatment vs No pretreatment: miscarriage rate	4

2.2 Pretreatment with Estrogen	4

FigureS2.2.1 Estrogen pretreatment vs No pretreatment: metaphase II oocytes	4

FigureS2.2.2 Estrogen pretreatment vs No pretreatment: live birth rate	5

FigureS2.2.3 Estrogen pretreatment vs No pretreatment: cumulative live birth rate	5

FigureS2.2.4 Estrogen pretreatment vs No pretreatment: Implantation rate	6

FigureS2.2.5 Estrogen pretreatment vs No pretreatment: clinical pregnancy rate	6

FigureS2.2.6 Estrogen pretreatment vs No pretreatment: miscarriage rate	6

FigureS2.2.7 Estrogen pretreatment vs No pretreatment: endometrial thickness	7

2.3 Pretreatment with GnRH-ant	7

FigureS2.3.1 GnRH-ant pretreatment vs No pretreatment: implantation rate	7

FigureS2.3.2 GnRH-ant pretreatment vs No pretreatment: moderate, severe risk of OHSS	8

FigureS2.3.3 GnRH-ant pretreatment vs No pretreatment: live birth rate	8

FigureS2.3.4 GnRH-ant pretreatment vs No pretreatment: clinical pregnancy rate	9

FigureS2.3.5 GnRH-ant pretreatment vs No pretreatment: metaphase II oocytes	9

FigureS2.3.6 GnRH-ant pretreatment vs No pretreatment: miscarriage rate	10

FigureS2.3.7 GnRH-ant pretreatment vs No pretreatment: ectopic pregnancy rate	10

2.4 Pretreatment with Testosterone	10

FigureS2.4.1 Testosterone pretreatment vs No pretreatment: endometrial thickness	11

FigureS2.4.2 Testosterone pretreatment vs No pretreatment: live birth rate	11

FigureS2.4.3 Testosterone pretreatment vs No pretreatment: implantation rate	12

2.5 Pretreatment with Progestogen	12

FigureS2.5.1 Progestogen pretreatment vs No pretreatment: oocytes retrieved	12

FigureS2.5.2 Progestogen pretreatment vs No pretreatment: live birth rate	13

FigureS2.5.3 Progestogen pretreatment vs No pretreatment: clinical pregnancy rate	13

PICO 3. Should fixed regimen or flexible regimen be preferred under GnRH-ant protocol?	1

FigureS3.1 Fixed group vs Flexible group: cumulative live birth rate	1

FigureS3.2 Fixed group vs Flexible group: implantation rate	1

FigureS3.3 Fixed group vs Flexible group: moderate risk of OHSS	1

FigureS3.4 Flexible group vs Fixed group: endometrial thickness	2

PICO 4. Is early initiation of GnRH-ant during stimulation recommended?	3

FigureS4.1 Early administration vs Late administration: clinical pregnancy rate	3

FigureS4.2 Early administration vs Late administration: implantation rateⅠ	3

FigureS4.3 Early administration vs Late administration: implantation rate Ⅱ	4

FigureS4.4 Early administration vs Late administration: live birth rate	4

FigureS4.5 Early administration vs Late administration: embryo implantation rate	4

FigureS4.6 Early administration vs Late administration: metaphase II oocytes	4

FigureS4.7 Early administration vs Late administration: high-quality embryos	5

FigureS4.8 Early administration vs Late administration: endometrial thickness	5

PICO 5. Is the GnRHa add-on trigger recommended over the HCG trigger alone?	1

5.1 Add-on GnRHa trigger to HCG and HCG alone	1

FigureS5.1.1 GnRHa + hCG vs hCG: live birth rate	1

FigureS5.1.2 GnRHa + hCG vs hCG: cumulative live birth rate	1

FigureS5.1.3 GnRHa + hCG vs hCG: implantation rate	2

FigureS5.1.4 GnRHa + hCG vs hCG: clinical pregnancy rates	2

FigureS5.1.5 GnRHa + hCG vs hCG: MII oocytes	3

FigureS5.1.6 GnRHa + hCG vs hCG: miscarriage rates	3

FigureS5.1.7 GnRHa + hCG vs hCG: endometrial thickness	4

5.2 GnRHa and HCG alone	4

FigureS5.2.1 GnRHa vs hCG: MII oocytes	4

FigureS5.2.2 GnRHa vs hCG: endometrial thickness	5

FigureS5.2.3 GnRHa vs hCG: implantation rate	5

FigureS5.2.4 GnRHa vs hCG: clinical pregnancy rate	5

FigureS5.2.5 GnRHa vs hCG: miscarriage rate	6

PICO 6. For individuals undergoing fresh embryo transfer with GnRHa trigger, is reinforced luteal support recommended?	1

FigureS6.1 Enhanced luteal support vs Standard luteal support: ongoing pregnancies	1

FigureS6.2 Enhanced luteal support vs Standard luteal support: early pregnancy losses	1

FigureS6.3 S Enhanced luteal support vs Standard luteal support: oocytes retrieved	1

PICO 7. Fresh embryo transfer compared with frozen-all strategy for GnRH-ant protocol?	1

7.1 High Ovarian Response	1

FigureS7.1.1 Fresh embryo transfer vs Frozen embryo transfer: moderate risk of OHSS	1

FigureS7.1.2 Fresh embryo transfer vs Frozen embryo transfer: live birth rate	1

FigureS7.1.3 Fresh embryo transfer vs Frozen embryo transfer: clinical pregnancy rate	2

FigureS7.1.4 Fresh embryo transfer vs Frozen embryo transfer: preeclampsia	2

FigureS7.1.5 Fresh embryo transfer vs Frozen embryo transfer: gestational hypertension	2

7.2 Normal Ovarian Response	2

FigureS7.2.1 Fresh embryo transfer vs Frozen embryo transfer: preeclampsia	3

FigureS7.2.2 Fresh embryo transfer vs Frozen embryo transfer: gestational hypertension	3

FigureS7.2.3 Fresh embryo transfer vs Frozen embryo transfer: moderate risk of OHSS	4

FigureS7.2.4 Fresh embryo transfer vs Frozen embryo transfer: live birth rate	4

FigureS7.2.5 Fresh embryo transfer vs Frozen embryo transfer: implantation rate	5

FigureS7.2.6 Fresh embryo transfer vs Frozen embryo transfer: clinical pregnancy rate	5

PICO 8. Which is the preferred timing to perform the embryo transfer: Day 3 or Day 5?	1

FigureS8.1 Cleavage embryo transfer vs Blastocyst embryo transfer: risk of ectopic pregnancy	1

FigureS8.2 Cleavage embryo transfer vs Blastocyst embryo transfer: clinical pregnancy rate	1

FigureS8.3 Cleavage embryo transfer vs Blastocyst embryo transfer: multiple births	1

# PICO 1. Whether the GnRH-ant protocol is recommended over the GnRH agonist (GnRH-a) protocols?

## FigureS1.1 GnRH - ant vs GnRH - a: live birth rate

## FigureS1.2 GnRH - ant vs GnRH - a: cumulative live birth rate

## FigureS1.3 GnRH - ant vs GnRH - a: moderate to severe OHSS

## FigureS1.4 GnRH - ant vs GnRH - a: clinical pregnancy rate

## FigureS1.5 GnRH - ant vs GnRH - a: implantation rate

## FigureS1.6 GnRH - ant vs GnRH - a: metaphase II oocytes

## FigureS1.7 GnRH - ant vs GnRH - a: endometrial thickness

## FigureS1.8 GnRH - ant vs GnRH - a: miscarriage rate

# PICO 2. Among patients undergoing IVF assisted reproduction with COS using GnRH-ant, is it recommended to implement pretreatments?

## 2.1 Pretreatment with Oral Contraceptive Pill (OCP)

### FigureS2.1.1 OCP pretreatment vs No pretreatment: metaphase II oocytes

### FigureS2.1.2 OCP pretreatment vs No pretreatment: moderate risk of OHSS

### FigureS2.1.3 OCP pretreatment vs no pretreatment: live birth rate

### FigureS2.1.4 OCP pretreatment vs No pretreatment: cumulative live birth rate

### FigureS2.1.5 OCP pretreatment vs No pretreatment: endometrial thickness

### FigureS2.1.6 OCP pretreatment vs No pretreatment: clinical pregnancy rate

### FigureS2.1.7 OCP pretreatment vs No pretreatment: miscarriage rate

## 2.2 Pretreatment with Estrogen

### FigureS2.2.1 Estrogen pretreatment vs No pretreatment: metaphase II oocytes

### FigureS2.2.2 Estrogen pretreatment vs No pretreatment: live birth rate

### FigureS2.2.3 Estrogen pretreatment vs No pretreatment: cumulative live birth rate

### FigureS2.2.4 Estrogen pretreatment vs No pretreatment: Implantation rate

### FigureS2.2.5 Estrogen pretreatment vs No pretreatment: clinical pregnancy rate

### FigureS2.2.6 Estrogen pretreatment vs No pretreatment: miscarriage rate

### FigureS2.2.7 Estrogen pretreatment vs No pretreatment: endometrial thickness

## 2.3 Pretreatment with GnRH-ant

FigureS2.3.1 GnRH-ant pretreatment vs No pretreatment: implantation rate

FigureS2.3.2 GnRH-ant pretreatment vs No pretreatment: moderate to severe risk of OHSS

FigureS2.3.3 GnRH-ant pretreatment vs No pretreatment: live birth rate

FigureS2.3.4 GnRH-ant pretreatment vs No pretreatment: clinical pregnancy rate

FigureS2.3.5 GnRH-ant pretreatment vs No pretreatment: metaphase II oocytes

FigureS2.3.6 GnRH-ant pretreatment vs No pretreatment: miscarriage rate

FigureS2.3.7 GnRH-ant pretreatment vs No pretreatment: ectopic pregnancy rate

## 2.4 Pretreatment with Testosterone

FigureS2.4.1 Testosterone pretreatment vs No pretreatment: endometrial thickness

FigureS2.4.2 Testosterone pretreatment vs No pretreatment: live birth rate

FigureS2.4.3 Testosterone pretreatment vs No pretreatment: implantation rate

## 2.5 Pretreatment with Progestogen

FigureS2.5.1 Progestogen pretreatment vs No pretreatment: oocytes retrieved

FigureS2.5.2 Progestogen pretreatment vs No pretreatment: live birth rate

FigureS2.5.3 Progestogen pretreatment vs No pretreatment: clinical pregnancy rate

# PICO 3. Should fixed regimen or flexible regimen be preferred under GnRH-ant protocol?

## FigureS3.1 Fixed group vs Flexible group: cumulative live birth rate

## FigureS3.2 Fixed group vs Flexible group: implantation rate

## FigureS3.3 Fixed group vs Flexible group: moderate risk of OHSS

## FigureS3.4 Flexible group vs Fixed group: endometrial thickness

# PICO 4. Is early initiation of GnRH-ant during stimulation recommended?

## FigureS4.1 Early administration vs Late administration: clinical pregnancy rate

## FigureS4.2 Early administration vs Late administration: implantation rateⅠ

## FigureS4.3 Early administration vs Late administration: implantation rate Ⅱ

## FigureS4.4 Early administration vs Late administration: live birth rate

## FigureS4.5 Early administration vs Late administration: embryo implantation rate

## FigureS4.6 Early administration vs Late administration: metaphase II oocytes

## FigureS4.7 Early administration vs Late administration: high-quality embryos

## FigureS4.8 Early administration vs Late administration: endometrial thickness

# PICO 5. Is the GnRHa add-on trigger recommended over the HCG trigger alone?

## 5.1 Add-on GnRHa trigger to HCG and HCG alone

FigureS5.1.1 GnRHa + hCG vs hCG: live birth rate

FigureS5.1.2 GnRHa + hCG vs hCG: cumulative live birth rate

FigureS5.1.3 GnRHa + hCG vs hCG: implantation rate

FigureS5.1.4 GnRHa + hCG vs hCG: clinical pregnancy rates

FigureS5.1.5 GnRHa + hCG vs hCG: MII oocytes

FigureS5.1.6 GnRHa + hCG vs hCG: miscarriage rates

FigureS5.1.7 GnRHa + hCG vs hCG: endometrial thickness

## 5.2 GnRHa and HCG alone

FigureS5.2.1 GnRHa vs hCG: MII oocytes

FigureS5.2.2 GnRHa vs hCG: endometrial thickness

FigureS5.2.3 GnRHa vs hCG: implantation rate

FigureS5.2.4 GnRHa vs hCG: clinical pregnancy rate

FigureS5.2.5 GnRHa vs hCG: miscarriage rate

# PICO 6. For individuals undergoing fresh embryo transfer with GnRHa trigger, is reinforced luteal support recommended?

## FigureS6.1 Enhanced luteal support vs Standard luteal support: ongoing pregnancies

## FigureS6.2 Enhanced luteal support vs Standard luteal support: early pregnancy losses

## FigureS6.3 S Enhanced luteal support vs Standard luteal support: oocytes retrieved

# PICO 7. Fresh embryo transfer compared with frozen-all strategy for GnRH-ant protocol?

## 7.1 High Ovarian Response

FigureS7.1.1 Fresh embryo transfer vs Frozen embryo transfer: moderate risk of OHSS

FigureS7.1.2 Fresh embryo transfer vs Frozen embryo transfer: live birth rate

FigureS7.1.3 Fresh embryo transfer vs Frozen embryo transfer: clinical pregnancy rate

FigureS7.1.4 Fresh embryo transfer vs Frozen embryo transfer: preeclampsia

FigureS7.1.5 Fresh embryo transfer vs Frozen embryo transfer: gestational hypertension

## 7.2 Normal Ovarian Response

FigureS7.2.1 Fresh embryo transfer vs Frozen embryo transfer: preeclampsia

FigureS7.2.2 Fresh embryo transfer vs Frozen embryo transfer: gestational hypertension

FigureS7.2.3 Fresh embryo transfer vs Frozen embryo transfer: moderate risk of OHSS

FigureS7.2.4 Fresh embryo transfer vs Frozen embryo transfer: live birth rate

FigureS7.2.5 Fresh embryo transfer vs Frozen embryo transfer: implantation rate

FigureS7.2.6 Fresh embryo transfer vs Frozen embryo transfer: clinical pregnancy rate

# PICO 8. Which is the preferred timing to perform the embryo transfer: Day 3 or Day 5?

## FigureS8.1 Cleavage embryo transfer vs Blastocyst embryo transfer: risk of ectopic pregnancy

## FigureS8.2 Cleavage embryo transfer vs Blastocyst embryo transfer: clinical pregnancy rate

## FigureS8.3 Cleavage embryo transfer vs Blastocyst embryo transfer: multiple births
